# Red light mediated energy transfer catalysis *via* stable organic radicals

**DOI:** 10.1039/d6sc05431e

**Published:** 2026-09-01

**Authors:** Tzu-Hsuan Feng, Ana Paula Nita Vizcardo, Andrew Brian Pun

**Affiliations:** a Department of Chemistry and Biochemistry, University of California San Diego 92093 La Jolla CA USA abpun@ucsd.edu; b Program in Materials Science and Engineering, University of California San Diego 92093 La Jolla CA USA

## Abstract

Photocatalysis is a versatile tool for chemical synthesis but has largely relied on high energy ultraviolet (UV) or blue light excitation. Recently, efforts have been made to conduct photocatalysis with lower-energy (red light) excitation. It offers key advantages, such as improved selectivity with fewer side reactions, greater biocompatibility, and deeper light penetration through reaction media. However, current red light photocatalysts often require rare and expensive metals, have limited reaction scope, or are difficult to prepare. Herein, we report a stable organic radical, TTM-3PCz, as a red-light-absorbing energy transfer photocatalyst. The radical consists of a tris(2,4,6-trichlorophenyl)methyl moiety that is readily synthesized from inexpensive precursors. TTM-3PCz can be optically excited to a doublet state because of its open-shell character. This excited doublet state directly populates the reactive triplet excited state of various substrates *via* Dexter-type doublet-to-triplet energy transfer. We show that TTM-3PCz promotes a broad range of reactions, including photooxidation, cyclization, [2 + 2] cycloaddition, and isomerization. Its performance is on par with traditional rare-metal containing photocatalysts excited with high-energy light. We demonstrate stable organic radicals as a powerful new class of air-tolerant, rare-metal-free photocatalysts for red light mediated transformations.

## Abstract 2

We report a stable organic radical as a metal-free red light absorbing photocatalyst that can perform various transformations *via* a doublet-to-triplet energy transfer mechanism.

## Graphical abstract

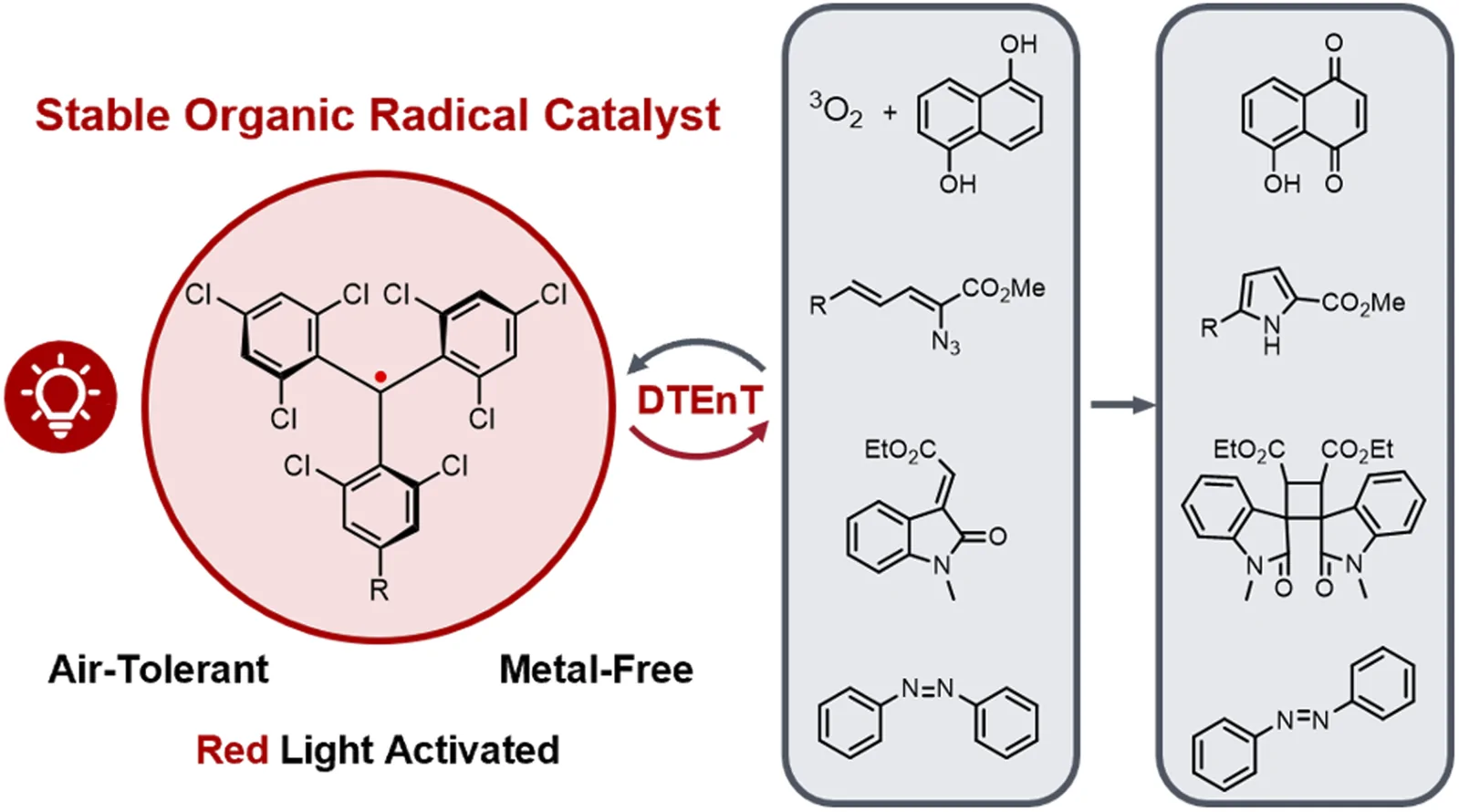

## Introduction

Photocatalysis utilizes energy from light to perform chemical transformation with high efficiency and selectivity under mild conditions. It has been extensively studied in past decades and has received widespread attention and application in modern synthetic organic chemistry.^1–7^ Photocatalysis *via* low-energy red light excitation is an emerging field that has begun to receive widespread attention.^8–11^ Red light photocatalysis possesses several advantages compared to conventionally employed ultraviolet (UV) or blue light excitation. These benefits include (i) fewer operational and health hazards; for example, long exposure to high-energy photons such as blue light may cause damage to tissues in the eye.^12,13^ (ii) Deeper penetration through various media.^14–17^ (iii) Fewer undesired side reactions and enhanced selectivity.^18–22^ Organic substrates commonly absorb higher-energy blue and UV light. Thus, red light excitation can prevent reagents, catalysts, and products from degrading.^23,24^ These advantages give red light photocatalysis great potential for application in fields as varied as biotherapeutics,^25–27^ bioimaging,^28–30^ polymer synthesis,^31–33^ and large-scale synthesis.^34,35^

Despite its great potential, the development of red light photocatalysts (PCs) remains rare (Scheme 1). Methylene blue^36^ and other red-light-absorbing organic dyes^37,38^ have long been used, but their reaction scopes are limited. In recent years, novel catalysts were explored to expand the reaction scope of red light PCs. Gianetti and coworkers showed dimethoxyquinacridinium (DMQA^+^) to be a powerful red light PC (Scheme 1A).^39–41^ DMQA^+^ can perform a wide range of demanding photoreductions and photooxidations,^39^ as well as chromoselective C(sp^2^)–X bond activation.^19^ Despite this, DMQA^+^ synthesis can be challenging, as starting materials are not readily accessible.^39,42–44^ In addition to organic dyes, heavy-metal-containing polypyridyl complexes have been explored, achieving red-shifted absorption by incorporating red light-absorbing organic dyes^45,46^ or extended π-conjugated systems onto the ligands.^47–49^ (Scheme 1A). Rovis and coworkers developed Os-based PCs that exploit a normally spin-forbidden S_0_ → T_1_ transition to absorb red light.^22,34^ Furthermore, it was recently demonstrated that Ru(bpy)_3_^2+^ can also successfully carry out a variety of reactions under red light excitation *via* a spin-forbidden S_0_ → T_1_ transition.^50^ These results suggest a growing effort toward designing strong red-light-absorbing photocatalysts based on metal complexes. While these heavy metal complexes can carry out a wide range of reactions, their synthesis and purification can be challenging. Reliance on rare and expensive metals like iridium, ruthenium, and osmium further hinders the widespread adoption of these catalysts, and the use of osmium raises additional toxicity concerns. Overall, these examples highlight the importance of exploring new classes of red-light photocatalysts, particularly metal-free scaffolds.

**Scheme 1:**
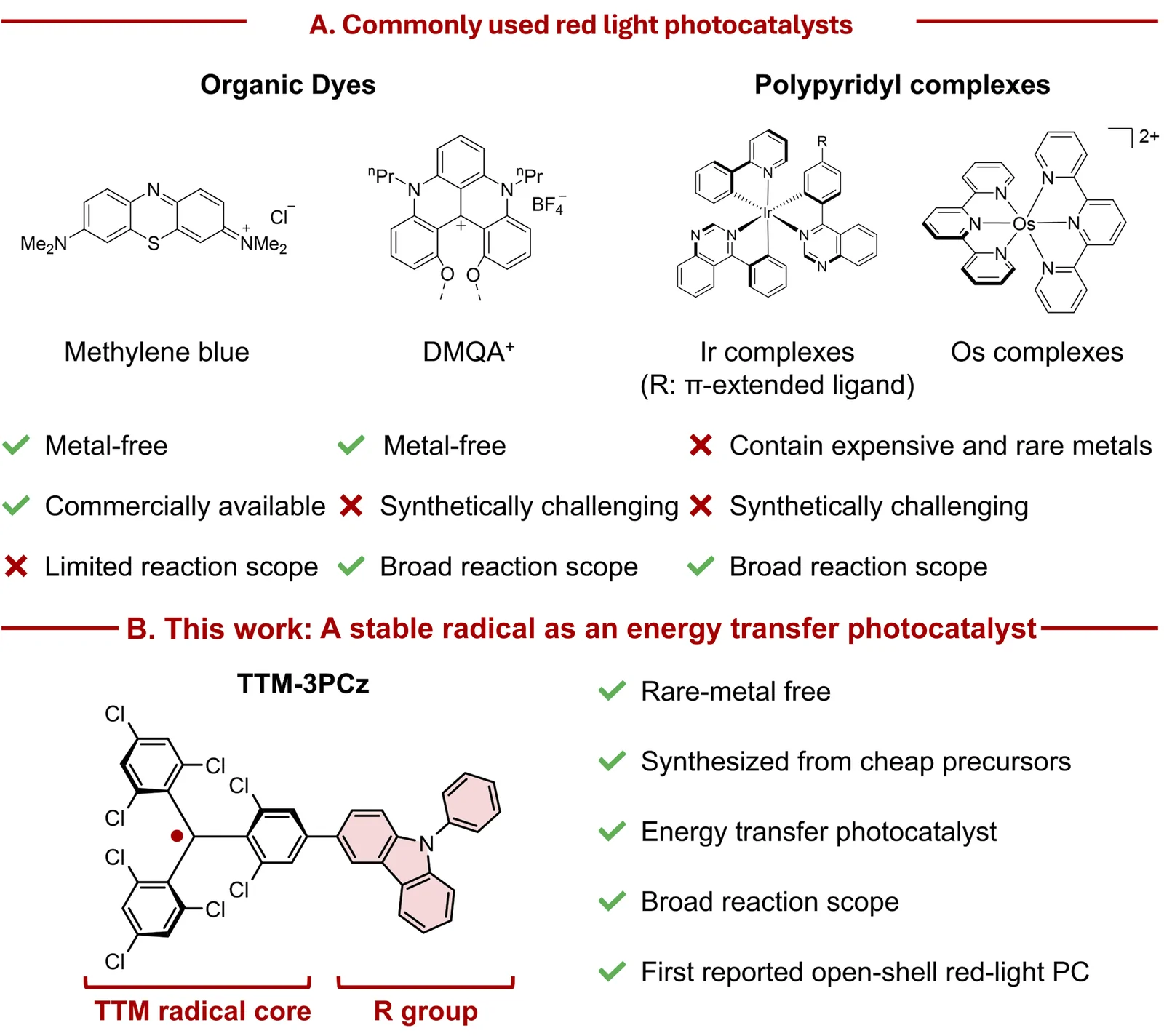
Red light photocatalysts.

Most red light photocatalysis reported to date has been performed *via* a single electron transfer (SET) mechanism, where the excited photocatalyst formally reduces or oxidizes a substrate. This pathway generates radical cation/anion intermediates and often requires sacrificial reagents to complete the catalytic cycle.^51,52^ By contrast, red light photocatalysis powered through energy transfer (EnT) remains scarce despite its conceptual simplicity. In EnT catalysis, a photoexcited PC transfers its excitation energy to a substrate, thereby populating a reactive excited state that can undergo a desired chemical transformation.^53–55^ In this study, we sought to develop metal-free, economical, and synthetically accessible PCs for EnT photocatalysis under low-energy red light excitation.

Herein, we report the use of a stable tris(2,4,6-trichlorophenyl)methyl (TTM)-based radical, TTM-3PCz, as a new photocatalyst for red light mediated EnT photocatalysis (Scheme 1B). TTM radicals are highly stable due to (i) electronic delocalization through three neighboring aromatic rings attached to the central radical site and (ii) bulky *para*- and *ortho*-substituted chlorine atoms which create a twisted propeller structure that sterically shields the radical site.^56–59^ The optoelectronic properties of these radicals can readily be tuned by changing the R group substituted on the TTM core. Owing to their stability and tunable optical properties, TTM radicals have received widespread attention for use in OLEDs^56,60^ and spintronics.^58,61^ However, despite the surge of interest in TTM-based radicals as functional materials, their potential in other applications has remained underexplored. In this work, we utilize the spin-doublet character of TTM radicals to perform doublet-to-triplet energy transfer, thereby bypassing the energetic loss typically associated with intersystem crossing (ISC) in photocatalysis. This feature allows TTM radicals to promote a broad range of EnT reactions using only low-energy red light excitation. The open-shell character responsible for the attractive optoelectronic properties of TTM can be harnessed to drive catalytic transformations, establishing photocatalysis as a new application for this class of compounds.

## Results and discussion

### Design strategy: doublet radicals as energy transfer photocatalysts

In traditional EnT catalysis, the catalytically active state of a closed-shell photocatalyst is the excited triplet state (^3^PC*). This state is accessed when UV or blue light first excites the photocatalyst to its singlet excited state (^1^PC*), followed by intersystem crossing (ISC) to populate ^3^PC* (Fig. 1A, path a). The photocatalyst and the substrate then undergo triplet-to-triplet energy transfer (TTEnT, Fig. 1B), populating the substrate to its triplet excited state (^3^Sub*) and returning the photocatalyst to the ground state. Theoretically, the photon energy required for this process only needs to be higher than the energy gap between the ground state (S_0_) and triplet excited state (T_1_) of the substrate. In practice, there are numerous energetic loss pathways, notably when the photocatalyst undergoes ISC (^1^PC* to ^3^PC*, Fig. 1A). Recently, direct spin-forbidden S_0_ to T_1_ excitation was observed for osmium- and ruthenium-based complexes (Fig. 1A, path b).^62^ However, since an S_0_ to T_1_ transition is spin-forbidden, the transition is typically weak. For Ru(bpy)_3_^2+^, the extinction coefficient of the S_0_ to S_1_ transition is 300 times larger than that of S_0_ to T_1_, resulting in much slower photocatalytic rates under red light excitation, with reaction times typically spanning 24–96 hours.^63^

**Fig. 1:**
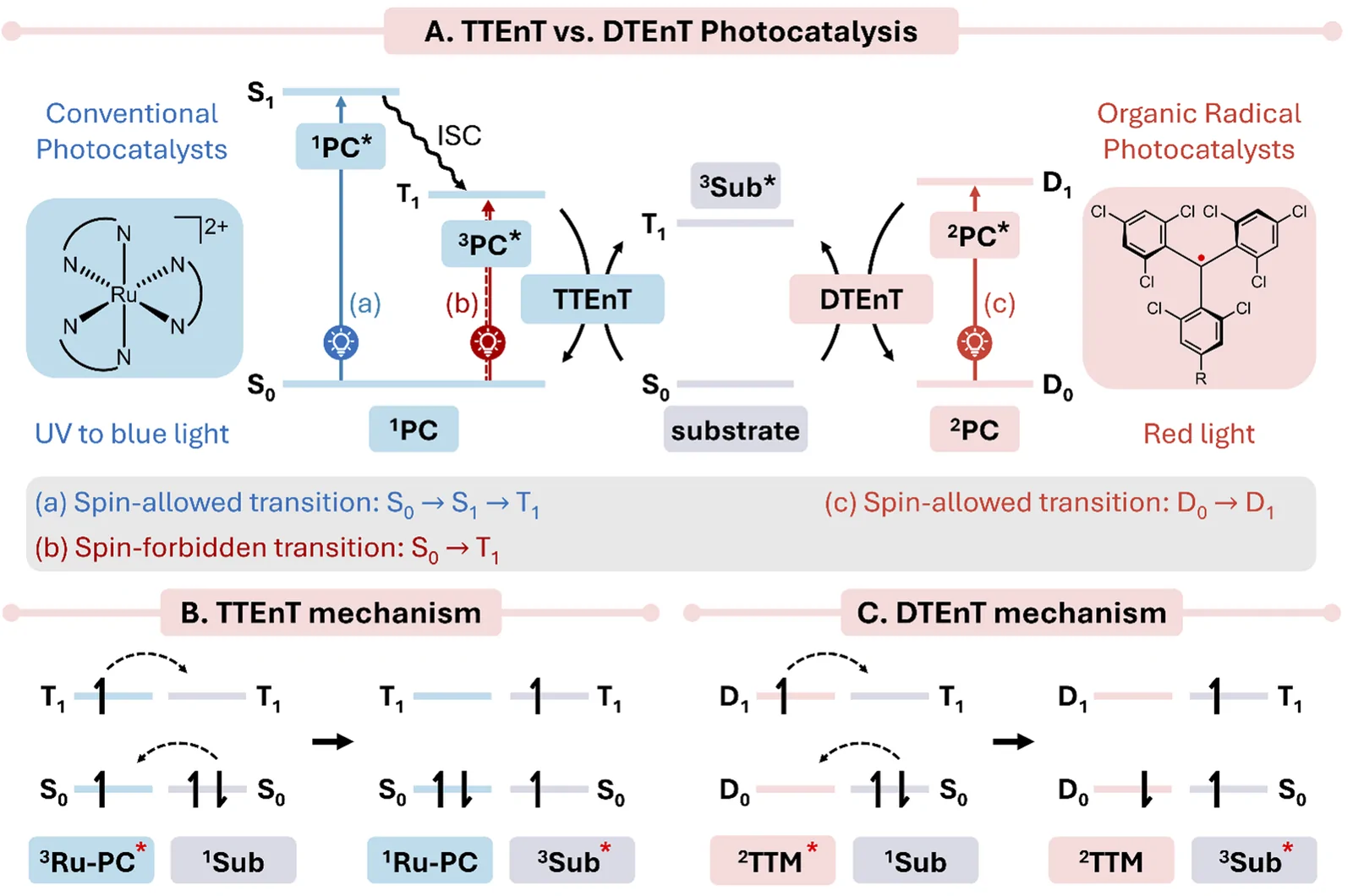
(A) Comparison of energy transfer (EnT) catalysis using a conventional ruthenium-based photocatalyst *via* triplet-to-triplet energy transfer (TTEnT) and this work, employing a TTM organic radical *via* doublet-to-triplet energy transfer (DTEnT). (B) Mechanism of Dexter-type TTEnT and (C) DTEnT.

Therefore, our strategy exploits the open-shell character of TTM radicals to circumvent ISC *via* a process called doublet-to-triplet energy transfer (DTEnT, Fig. 1C). Because TTM radicals are spin doublets in both their ground state (D_0_) and excited state (D_1_), the D_0_ to D_1_ transition is spin-allowed without requiring ISC (Fig. 1A, path c). Through Dexter-type energy transfer, D_1_ energy is then transferred to the substrate, populating its triplet excited state. In previous studies, intermolecular and intramolecular DTEnT between TTM radicals and organic chromophores has been applied in triplet–triplet annihilation upconversion (TTA-UC).^64–66^ Intramolecular DTEnT between a metal center and chromophore ligands and intermolecular DTEnT between a metal complex and triplet annihilators have been shown to extend the excited-state lifetime of inorganic complexes, resulting in improved TTA-UC or catalytic performance.^67–69^ We are curious whether this underexplored DTEnT pathway can be harnessed to allow TTM radicals to act solely as a photocatalyst, directly driving chemical transformations with red light.

Notably, the doublet excited state energy of TTM radicals is roughly 1.9 to 2.0 eV,^70^ similar to the triplet excited state energy of Ru-based photocatalysts.^71^ Therefore, TTM radicals should be able to serve as photocatalysts in many of the same EnT reactions that required Ru-based PCs (Fig. 1A).

### Synthesis of the TTM radical

In this study, we chose a TTM radical with an *N*-arylated carbazole moiety (TTM-3PCz) as a stable radical PC due to its ease of synthesis, strong absorption of red light, and outstanding catalytic performance in our preliminary screens. The synthesis of TTM-3PCz was adapted from Li and coworkers.^60^ The route begins with a Friedel–Crafts alkylation between chloroform and 1,3,5-trichlorobenzene, followed by Suzuki–Miyaura cross-coupling to form the protonated precursor (HTTM-3PCz). Deprotonation and oxidation of this species generate the final radical (TTM-3PCz). The three-step synthesis proceeds in an overall 15% yield. While the yield is low overall, the precursors used in the synthesis are cheap, and the palladium catalyst required in the second step is used in minimal loading. We also note that in most TTM radical syntheses, the radicalization step often does not have full conversion, and the remaining protonated starting material is present after the reaction.^57^ Therefore, we utilized HPLC to purify TTM-3PCz from its protonated counterpart. While the overall yield and purification requirements leave room for improvement, the use of cheap and commercially available precursors makes the radical material accessible. Furthermore, our photocatalyst is stable under ambient conditions with no observable photodegradation over the course of 14 days (solid TTM-3PCz was stored in a vial exposed to fume hood lighting, SI Section 3, Fig. S5 and S6). Synthetic details and photophysical properties of TTM-3PCz are included in the SI (Section S3).

### Singlet oxygen mediated photooxidation

A well-established use for red light mediated photocatalysis is to sensitize ground-state oxygen (^3^O_2_) to its reactive singlet excited state 
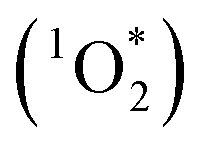
 to perform photooxidations.^72^ We first confirmed that TTM-3PCz is an effective EnT PC by performing the oxygen mediated oxidation of 1,5-dihydroxynaphthalene (1,5-DHN) to juglone. 1,5-DHN was dissolved in aerated acetonitrile and irradiated with deep red (660 nm) light for 19 hours. 1,5-DHN was converted to juglone with 38% isolated yield (Fig. 2). It is worth noting that oxygen concentration in acetonitrile in an ambient atmosphere typically lies in the range of 1–2 mM.^73^ Since TTM-3PCz has a short doublet excited state lifetime (21.2 ns),^60^ at these relatively low oxygen concentrations (∼2 mM), only a small population of excited state species (TTM-3PCz*) will undergo DTEnT with O_2_ before TTM-3PCz intrinsically relaxes to the ground state. To support this rationale, we conducted the reaction at higher O_2_ concentration, where the reaction mixture was sparged with O_2_ for at least 30 minutes and kept under an O_2_ balloon during red light irradiation. As expected, a boost of yield up to 84% was observed. This shows that increased oxygen concentration enhances the probability of TTM-3PCz DTEnT with O_2_. The connection between doublet excited-state lifetime and DTEnT to oxygen was also reported by Ayitou and coworkers. DTEnT to oxygen was observed with TTM radicals only when derivatives were synthesized that had long lifetimes (97.06 ns).^65^ When TTMs with shorter lifetimes (21 ns) were used, no appreciable DTEnT was observed, explaining the low yield of juglone formation in air. While photoexcited persistent radicals generated *in situ* have been previously used,^74^ our DHN oxidation reaction is the first proof-of-concept of red light photocatalysis *via* DTEnT using stable organic radicals as photocatalysts. To explore the tolerance of TTM-3PCz to the oxidation reaction conditions, we isolated the catalyst after the reaction by flash chromatography (see Section S8, Table S6). After 19 hours of red light irradiation in air, over 90% of the initial TTM-3PCz was recovered, demonstrating the stability of this material. With this positive result in hand, we explored the use of TTM-3PCz in various other EnT photocatalytic reactions.

**Fig. 2:**
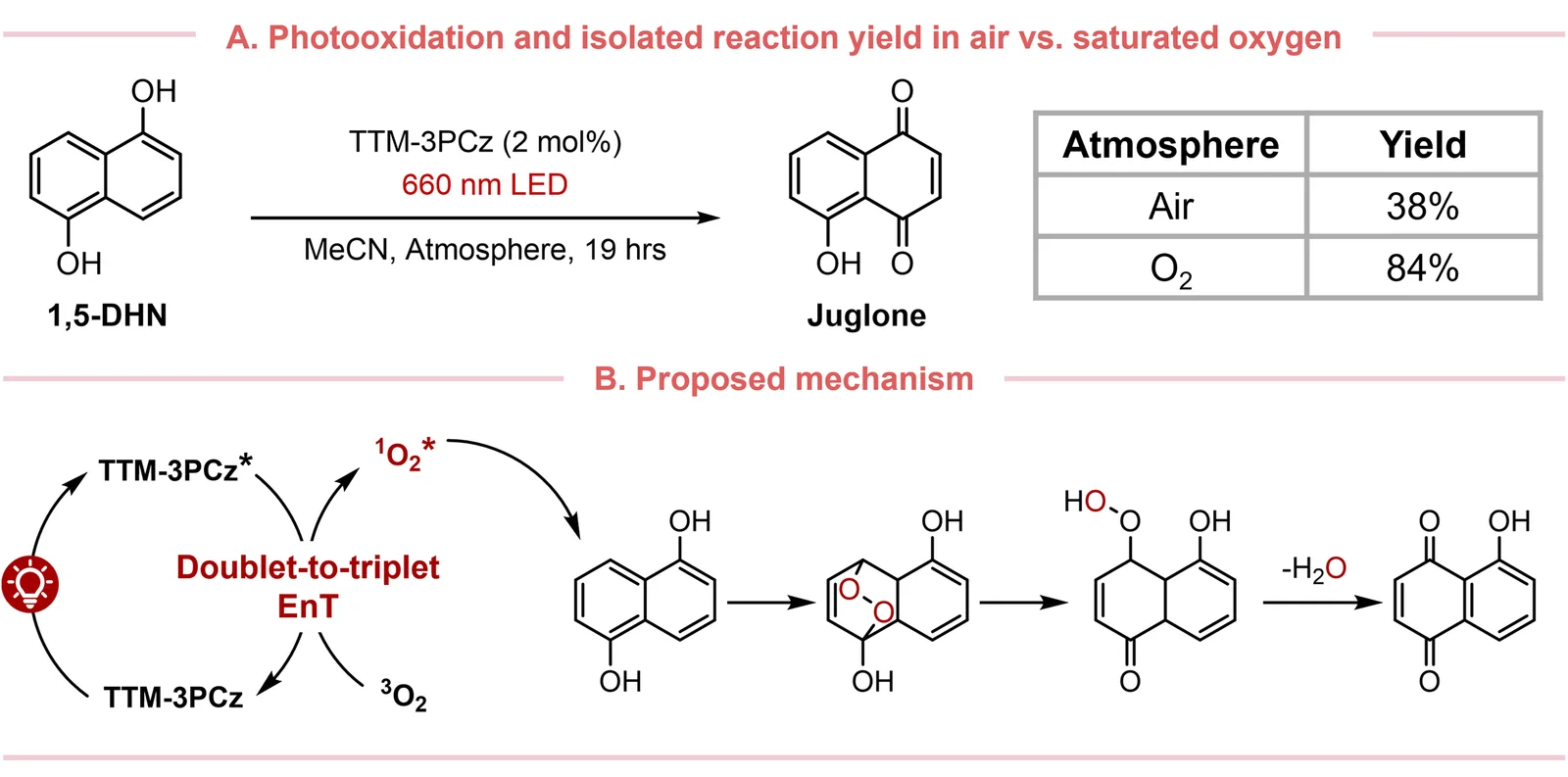
(A) Red light mediated oxidation of 1,5-DHN to juglone *via* singlet oxygen including isolated reaction yield in air *vs.* an oxygen atmosphere. Reactions were conducted using 0.12 mmol of 1,5-DHN and 2 mol% of TTM-3PCz in anhydrous MeCN (11 mM) and irradiated with a 660 nm LED (100 W) in a photoreactor for 19 h. Air and oxygen gas were respectively purged into reaction mixtures for at least 30 min. The reaction was performed in an ambient environment. (B) Proposed mechanism for this transformation.

### Photocatalytic dienyl azide cyclization

We next investigated the EnT catalyzed transformation of dienyl azides into pyrroles first reported by Yoon and coworkers with ruthenium or iridium PCs under blue light irradiation.^75^ The excited state triplet energy of a phenyl substituted dienyl azide 1 was calculated to be 1.56 eV,^76^ making it suitable to accept energy from TTM-3PCz *via* DTEnT. We performed this reaction under deep red (660 nm) light excitation, replacing the ruthenium PC with TTM-3PCz. Under our optimized conditions (Fig. 3 entry 1) pyrrole 2 was formed with 93% yield after only 1 hour. Notably, the yield improved when the catalyst loading was increased from 0.01 to 0.1 mol% (Fig. 3 entries 2 and 1 respectively). However, yield decreased when catalyst concentration was ten and fifty times higher than the optimized conditions (Fig. 3 entries 3 and 4). We suspect that when TTM-3PCz concentration is sufficiently high, red light will mostly be absorbed near the surface of the reaction vessel, hindering its penetration through the whole reaction media. Consequently, a portion of TTM-3PCz will be left non-activated and unable to participate in energy transfer events. Control experiments demonstrated that both the TTM-3PCz catalyst and light were required to drive the reaction (Fig. 3 entries 5 and 6). The product yield remained virtually unchanged when the reaction mixture was purged with air (Fig. 3 entry 7), indicating minimal oxygen quenching and an air tolerant reaction. Stern–Volmer quenching experiments revealed that the energy transfer rate between substrate 1 and TTM-3PCz is 6.6 × 10^8^ M^−1^ s^−1^ (see Fig. S12 and S13). This value is comparable to typical oxygen quenching rate constants (∼10^9^ M^−1^ s^−1^). Given these similar rates, because the concentration of substrate 1 (89 mM) is much higher than the concentration of oxygen in air-saturated tetrahydrofuran (∼2 mM),^77^ DTEnT is favored to occur between TTM-3PCz and substrate 1.

**Fig. 3:**
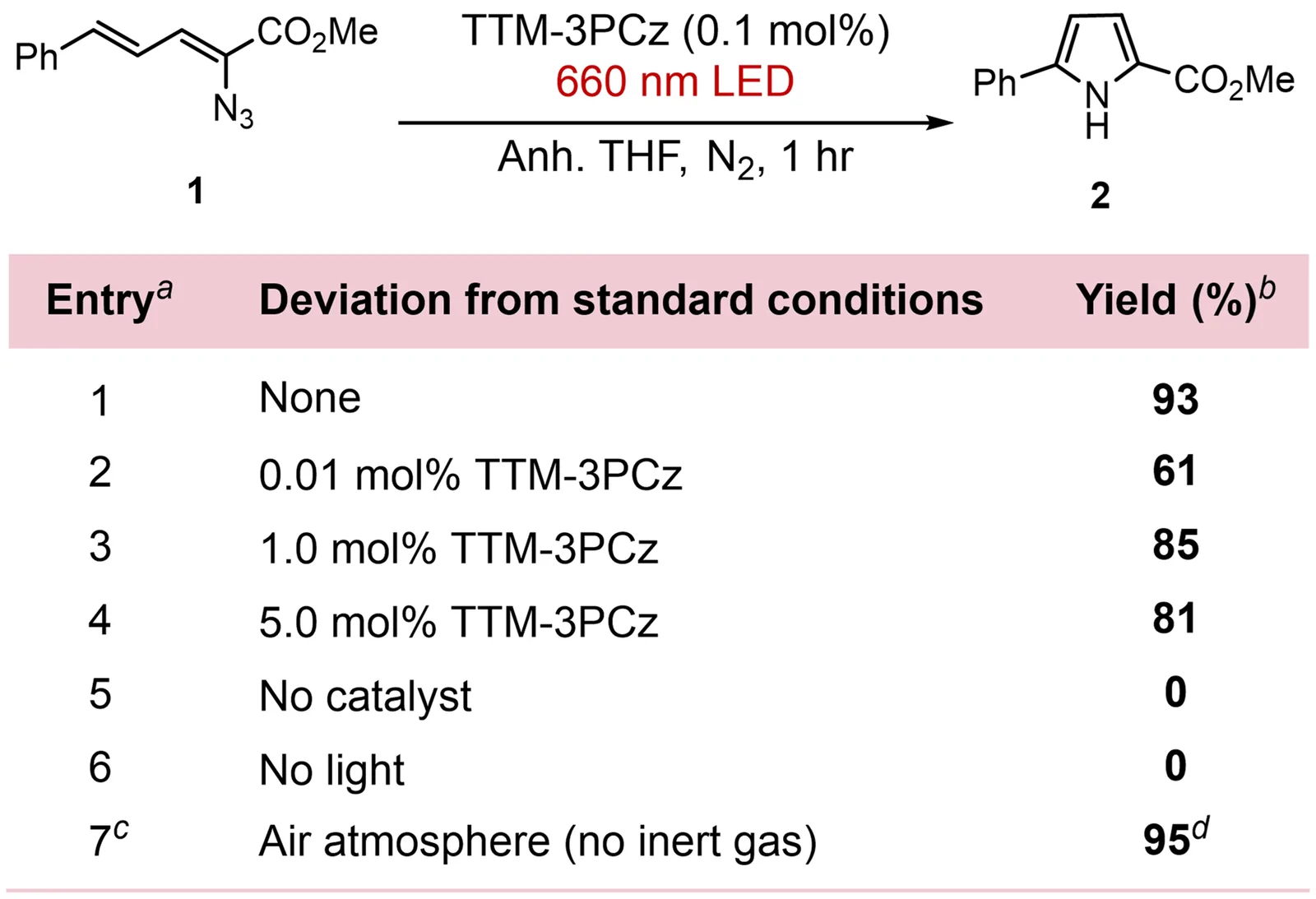
Optimization of red light mediated dienyl azide to pyrrole cyclization. ^*a*^Unless otherwise noted, all reactions were conducted using 0.044 mmol of 1 in anhydrous THF (89 mM) and irradiated with a 660 nm LED (100 W) in a photoreactor for 1 h. Reaction mixtures were prepared in a nitrogen glovebox. ^*b*^Yields were determined by ^1^H NMR analysis of the crude reaction mixtures using 1,3,5-dimethoxybenzene as an internal standard. ^*c*^0.22 mmol of 1 was dissolved in anhydrous THF (100 mM) and purged with air for 30 min. The reaction was performed in an ambient environment. ^*d*^Isolated yield.

We next explored the substrate scope of the red light mediated dienyl azide to pyrrole transformation (Fig. 4). With aryl substituents, over 90% isolated yield is obtained within one hour of red light irradiation. Both electron-donating and electron-withdrawing groups (Fig. 4 entries 2 and 3, respectively) were compatible with the reaction. While higher catalyst loading and irradiation time were required, good yield (71% isolated) was also observed when using an aliphatic dienyl azide starting material.

**Fig. 4:**
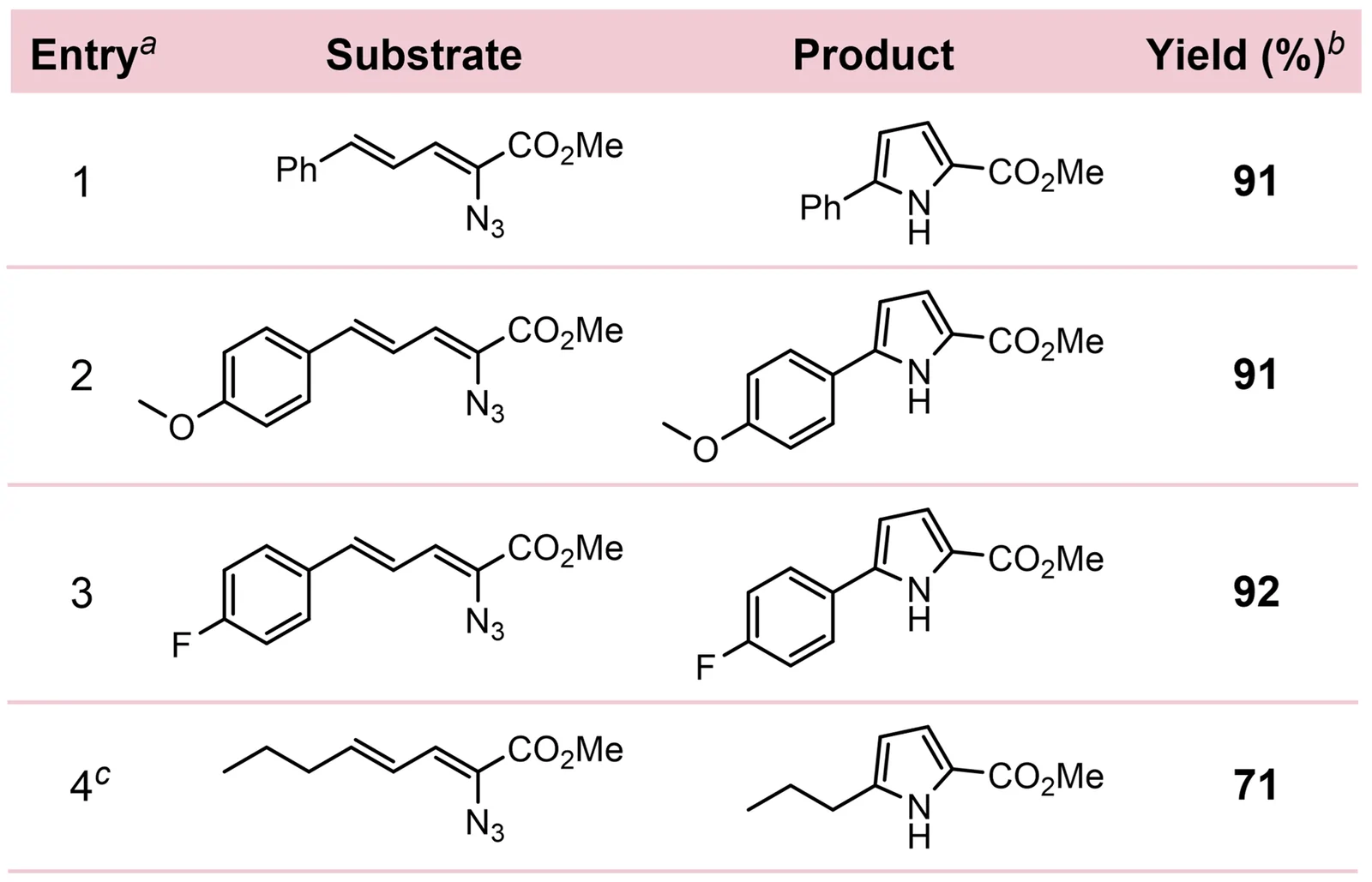
Scope of red light mediated dienyl azide to pyrrole cyclization. ^*a*^Unless otherwise noted, reaction mixtures were prepared in a nitrogen glovebox using 50 mg (∼0.2 mmol, 1 equiv.) of substrates, TTM-3PCz (0.1 mol%) and anhydrous THF (100 mM). Reactions were irradiated with a 660 nm LED (100 W) for 1 h in a photoreactor. ^*b*^Yields shown are the average of isolated yields from three trials. ^*c*^TTM-3PCz (1 mol%) was used and reactions were irradiated with a 660 nm LED (100 W) for 6 h.

### Photocatalytic [2 + 2] cycloaddition

To further demonstrate the generality of stable organic radicals as EnT catalysts, we investigated photocatalytic intermolecular [2 + 2] cycloadditions. This type of transformation is known to proceed *via* an EnT mechanism,^78,79^ but has predominantly been performed under UV-light or blue light irradiation.^80–82^ We selected the [2 + 2] cycloaddition of 3-ylideneoxindoles as our target reaction. Xiao and coworkers showed that this reaction can proceed with white light irradiation and Ru(bpy_3_)^2+^ as a PC.^83^ As before, given the similar excited-state energies of TTM-3PCz and Ru(bpy_3_)^2+^, we expected that TTM-3PCz could replace Ru(bpy_3_)^2+^ as an EnT PC to catalyze a [2 + 2] cycloaddition under red light excitation. The estimated triplet energy of 3-ylideneoxindoles (1.77 eV)^76^ is also below that of TTM-3PCz (1.80 eV), supporting the ability of TTM-3PCz to drive the reaction. After optimization, the cycloaddition was successfully performed with 72% yield after only one hour of deep red light excitation and 1 mol% TTM-3PCz catalyst loading (Fig. 5 entry 1). Like the dienyl azide reaction presented above, we observed a decrease in yield as catalyst loading exceeds 1 mol% (Fig. 5 entries 2 and 3). This reinforces our speculation that high concentration of TTM-3PCz will block light penetration into reaction media. As shown in Fig. 5 entry 4, extending the excitation time to 6 hours resulted in a slight increase in yield. As we scale the reaction to 0.2 mmol starting material (Fig. 5 entry 5), 81% isolated yield was observed after 6 hours of irradiation. This is comparable to the yields reported by Xiao and coworkers; however, their conditions required 13 hours of white-light irradiation.^83^ Control experiments (Fig. 5 entries 6 and 7) suggested both the TTM-3PCz catalyst and deep red light are required for the [2 + 2] cycloaddition. As with our previous reactions, performing the cycloaddition in air did not lead to reduced yield (Fig. 5 entry 8). This demonstrates that our red light PC system is broadly air tolerant.

**Fig. 5:**
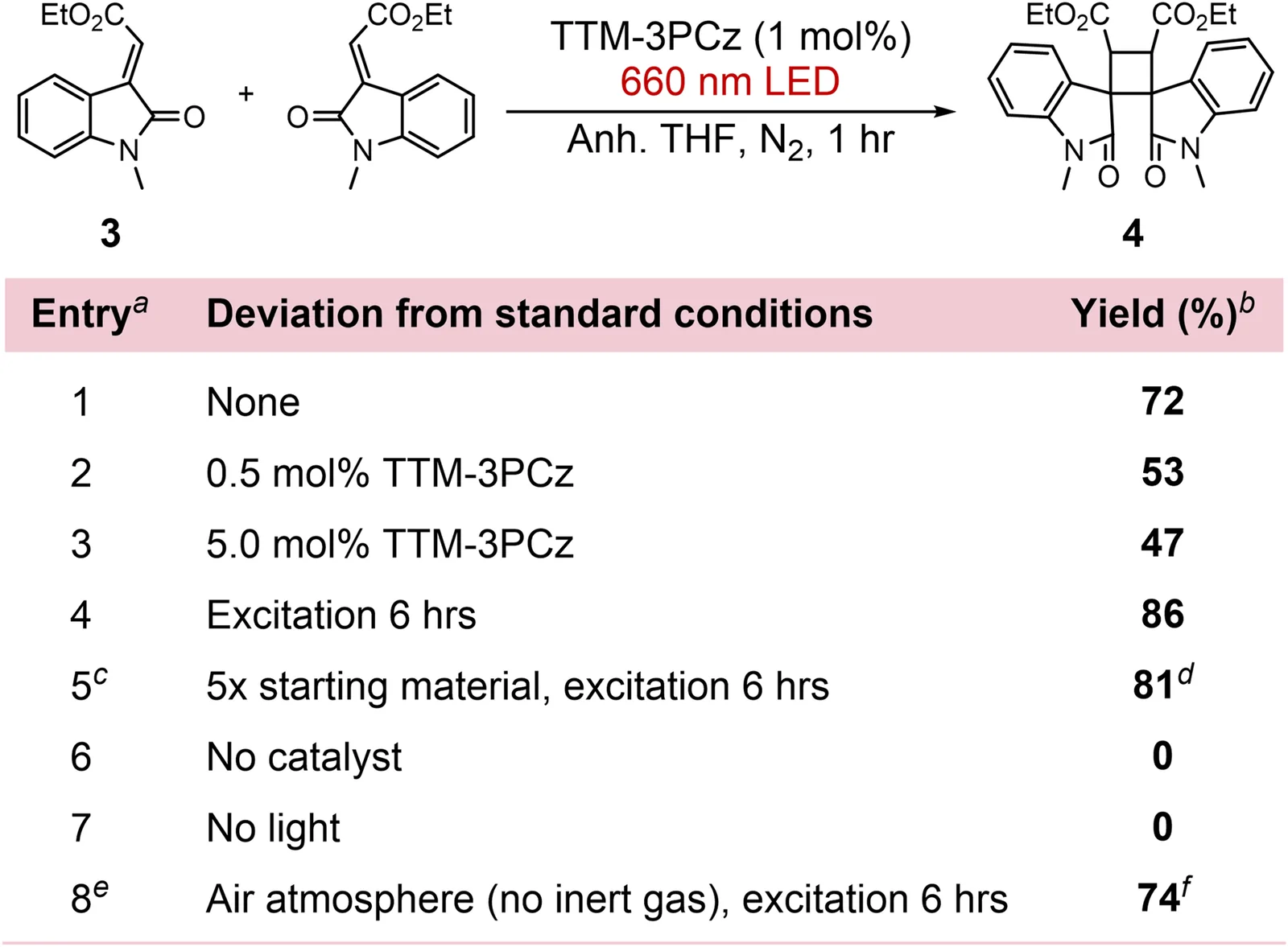
Optimization studies for red light mediated [2 + 2] cycloaddition. ^*a*^Unless otherwise noted, all reactions were conducted using 0.045 mmol of 3 in anhydrous THF (100 mM) and irradiated with a 660 nm LED (100 W) in a photoreactor for 1 h. Reaction mixtures were prepared in a nitrogen glovebox. ^*b*^Yields were determined by ^1^H NMR using 1,3,5-dimethoxybenzene as an internal standard. ^*c*^0.22 mmol of 3 was used. ^*d*^Yield shown is the average of isolated yields from three trials. ^*e*^0.22 mmol of 3 was dissolved in anhydrous THF that was purged with air for 30 min. The reaction was performed in an ambient environment. ^*f*^Isolated yield.

Furthermore, we examined the stability of TTM-3PCz under the [2 + 2] cyclization reaction conditions for both 1 hour and 6 hours of red-light irradiation (see Section S8, Table S6). While some degradation was observed with prolonged reaction time, over 80% of TTM-3PCz was recovered overall, suggesting good chemical tolerance of TTM-3PCz in our reaction system.

### Comparison of catalytic performance between TTM-3PCz and methylene blue

To further highlight the advantage of doublet photocatalysts in minimizing excited-state energy loss, we compared the red-light photocatalytic performance of TTM-3PCz and methylene blue, a commercially available red light photocatalyst. Methylene blue exhibits strong absorption in the red-light region similar to TTM-3PCz (Fig. 6A). Its singlet excited state (S_1_) is similar in energy to the D_1_ state of TTM-3PCz (Fig. 6B, 1.9 eV). However, this S_1_ state is short-lived and efficiently undergoes intersystem crossing to a low-lying, long-lived T_1_ state (Fig. 6B, 1.5 eV), which serves as the catalytically active state for EnT catalysis. The T_1_ state of methylene blue is sufficiently high in energy to populate the low-lying S_1_ of oxygen (Fig. 6B, *E*(S_1_) = 0.97 eV). As expected, methylene blue effectively sensitizes ground state oxygen to generate singlet oxygen and perform the oxidation of 1,5-DHN. In air, methylene blue produces a higher yield of juglone compared to TTM-3PCz, while under oxygen-saturated conditions, the two catalysts give comparable yields (Fig. 6C). The T_1_ energy of methylene blue is near the energetic threshold for sensitizing dienyl azide cyclization of compound 1 (Fig. 6B, *E*(T_1_) = 1.56 eV) and insufficient to promote [2 + 2] cycloaddition of compound 3 (Fig. 6B, *E*(T_1_) = 1.77 eV). In agreement with this analysis, methylene blue converted dienyl azide to the corresponding pyrrole in lower yield than TTM-3PCz, while no [2 + 2] cycloaddition product was observed (Fig. 6C). Together, these results demonstrate that DTEnT allows TTM-3PCz to engage directly in energy transfer from its doublet excited state, thereby avoiding energy loss from intersystem crossing and enabling photocatalysis under lower-photon energy irradiation.

**Fig. 6:**
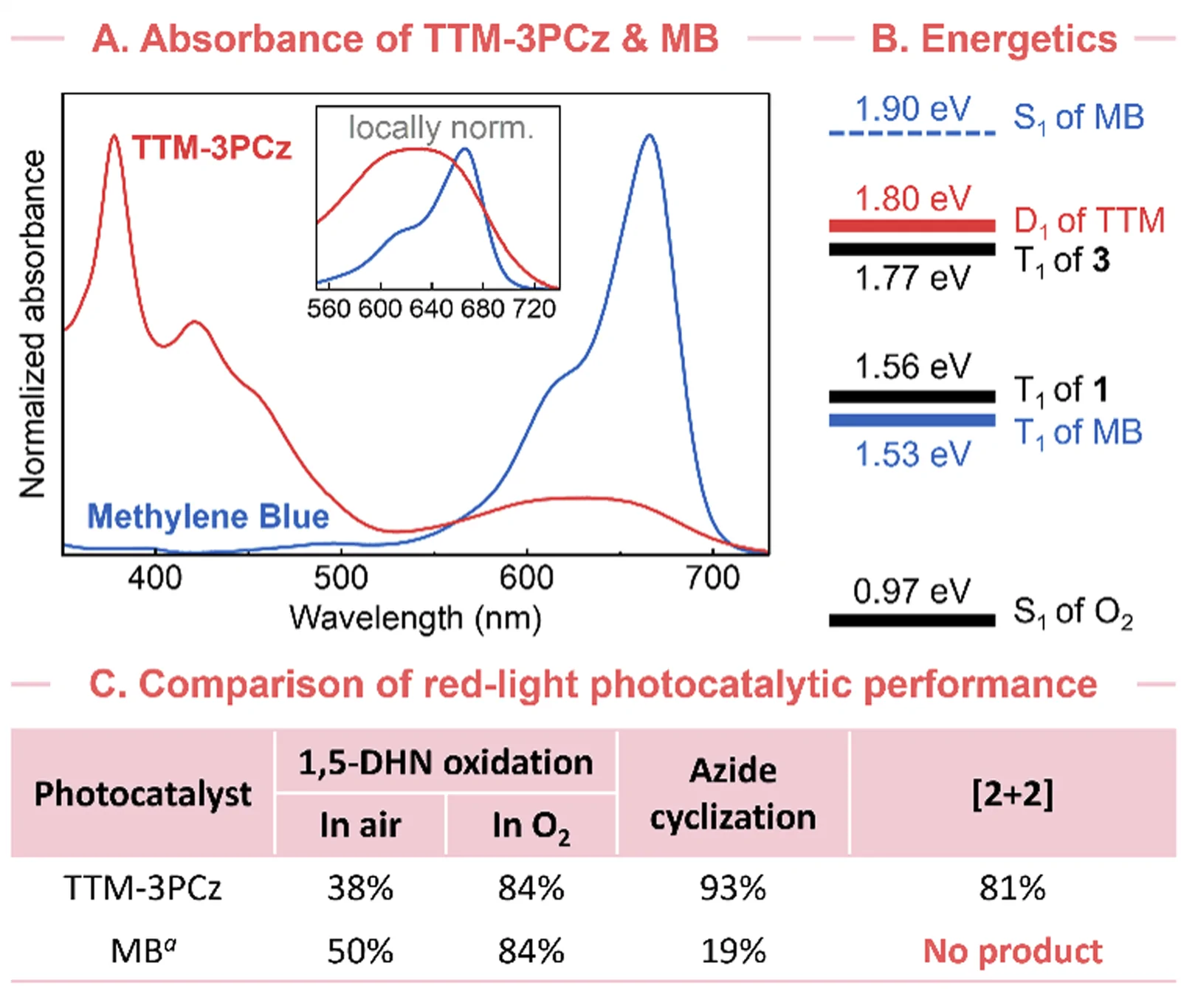
Comparison of the catalytic performance of TTM-3PCz and methylene blue (MB) under red light (660 nm) irradiation. (A) Normalized absorption spectrum of TTM-3PCz (red) and methylene blue (blue). Inset: locally normalized spectra in the 550–740 nm range. (B) Energy-level diagram of TTM-3PCz, methylene blue, singlet oxygen, compound 3 (3-ylideneoxindole, the starting material in the [2 + 2] cycloaddition) and compound 1 (phenyl dienyl azide, the starting material in the pyrrole formation reaction). The doublet excited-state (D_1_) energy of TTM-3PCz was estimated from the intercept of absorption and emission spectra. Excited-state energies of methylene blue, compound 1, and compound 3 were calculated using opensource EnTdecker.^76^ The excited-state energy of singlet oxygen was obtained from the literature.^84^ (C) Comparison of red-light-driven 1,5-DHN oxidation, dienyl azide cyclization and [2 + 2] cycloaddition using TTM-3PCz or methylene blue as the photocatalyst. Reactions were conducted under identical conditions; see SI Section S9 for details.

### Photocatalyzed azobenzene *Z* → *E* isomerization

Another common transformation performed *via* energy transfer photocatalysis is double bond isomerization.^85^ Light provides the energy to overcome the rotational barrier around the double bond, leading to a geometry change. We explored the photoisomerization of azobenzene with our radical red light PC. The geometry of azobenzene can change reversibly through exposure to different wavelengths of light. Thus, this class of molecules is often employed as photoswitches and smart photoresponsive materials for light-triggered drug release and energy storage, to name a few.^86^ Typically, azobenzene exists in the *trans* (*E*)-configuration as it is 12 kcal mol^−1^ more stable than the *cis* (*Z*)-configuration. Upon exposure to UV-light (320–365 nm), the *E*-form isomerizes to the metastable *Z*-form. While the latter will eventually return to the thermodynamically favored *E*-form, this process can be accelerated by heat or direct excitation with visible light (∼450 nm). Recent endeavors to red shift the excitation wavelength for this azobenzene *Z* → *E* isomerization have promising biological applications.^87,88^ Hence, we wanted to explore whether TTM-3PCz can serve as a sensitizer to promote azobenzene *Z* → *E* isomerization under deep red light irradiation (Fig. 7A). To demonstrate this, a mixed solution containing 30 µM azobenzene and 3 µM TTM-3PCz was prepared in deaerated toluene, with the absorption spectra shown in Fig. 7B. *E*-azobenzene shows strong absorption between 310 and 320 nm (π–π*) and weak absorption between 420 and 440 nm (n–π*).^89,90^ After 10–30 minutes exposure to a 340 nm LED, *E*-azobenzene was converted to the *Z*-form, with absorption bands at 280 nm (π–π*) and 440 nm (*n*–π*). Upon red light irradiation in the presence of our red light PC, the *Z*-form was converted back to *E*-azobenzene within 30 minutes (Fig. 7B), demonstrating effective isomerization with low-energy light by employing our TTM-3PCz catalyst. In addition, this isomerization is reversible. With six rounds of cycling, reversible isomerization of azobenzene was observed (Fig. 7C), showing that our photocatalyst is stable even under high energy UV-irradiation.

**Fig. 7:**
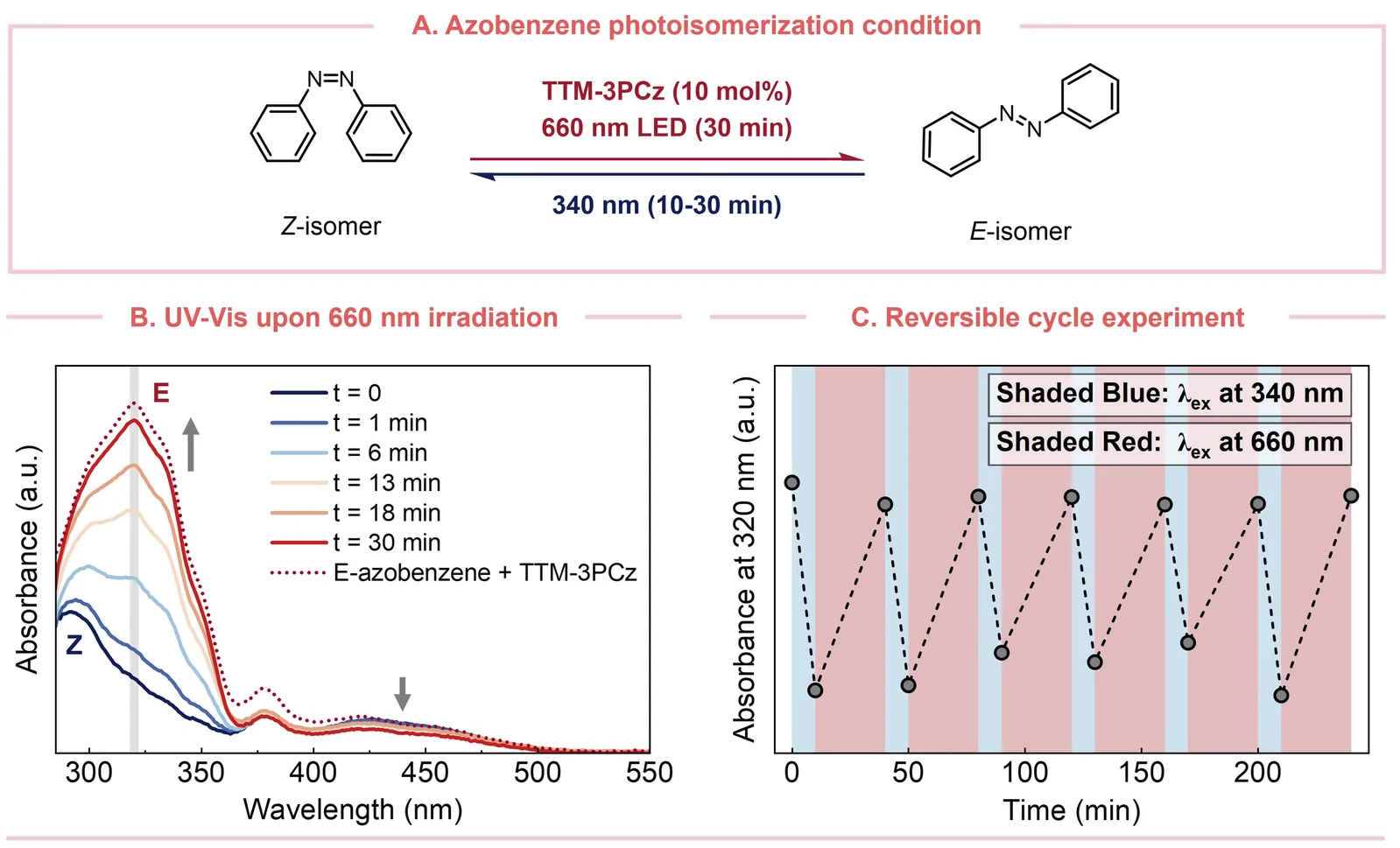
Azobenzene *Z* → *E* photoisomerization. (A) Reaction conditions for *Z* ↔ *E* photoisomerization. (B) Absorption spectrum of a mixture of *E*-azobenzene (30 µM) and TTM-3PCz (3 µM) in deaerated toluene (dotted red line) and changes in the spectra upon irradiation with 340 nm light (darkest navy line, *t* = 0, isomerized to *Z*-azobenzene) and upon irradiation with 660 nm light (blue to red gradient, *t* = 1/6/13/18/30 min, isomerized back to *E*-azobenzene). A sealed quartz cuvette with a 2 mm optical path was used to conduct UV-Vis measurements. See the SI for detailed measurement parameters. (C) Reversible *Z* ↔ *E* azobenzene isomerization by alternating between 340 nm (blue shaded region, 10 min) and 660 nm (red shaded region, 30 min) excitation.

## Conclusions

In conclusion, we have demonstrated a stable organic radical, TTM-3PCz, that can be employed as a red light absorbing energy transfer photocatalyst. By taking advantage of the doublet character of TTM-3PCz, spin-allowed doublet-to-triplet energy transfer was achieved, thereby bypassing the energy loss from intersystem crossing that is usually required in closed-shell photocatalysts. This enabled numerous EnT photocatalysis reactions to be performed, shifting away from high-energy UV/blue light to low-energy red light excitation. This finding opens a new avenue for stable organic radicals, moving beyond their application in OLEDs and spintronics and establishing photocatalysis as an additional domain where their unique electronic structure can be utilized. TTM-3PCz is free of rare-metals, synthetically accessible with cheap precursors, and can perform photocatalysis effectively under an ambient atmosphere. These advantages make it applicable to diverse reaction systems with operational simplicity. To the best of our knowledge, this study is the first example of red light photocatalysis that directly exploits the doublet character of stable organic radicals to perform desired chemical transformations *via* DTEnT. Moving forward, owing to the enhanced penetration of low-energy light, our strategy paves the way for *in vivo* biomedical applications and practical scale-up applications of organic radical photocatalysts.

## Author contributions

Tzu-Hsuan Feng: conceptualization, methodology, validation, investigation, writing – original draft, writing – review & editing. Ana Paula Nita Vizcardo: investigation. Andrew Brian Pun: conceptualization, methodology, project administration, supervision, writing – review & editing.

## Conflicts of interest

The authors declare no competing financial interest.

## Supplementary Material

SC-017-D6SC05431E-s001

## Data Availability

The data supporting this article have been included as part of the supplementary information (SI). Supplementary information: experimental procedures, catalyst synthesis and characterization, optical measurements, reaction optimization tables, and copies of ^1^H and ^13^C NMR spectra. The authors have cited additional references within the SI.^91,92^ See DOI: https://doi.org/10.1039/d6sc05431e.
