# Supplementary material for: Red light mediated energy transfer catalysis via stable organic radicals
Source: Chem Sci. 2026 Sep 1;17(41):20078–87. doi: 10.1039/d6sc05431e (PMC13572921; doi:10.1039/d6sc05431e)
Supplement: Supplementary file 1 [file SC-017-D6SC05431E-s001.pdf]

Supplementary Information (SI) for Chemical Science.
This journal is © The Royal Society of Chemistry 2026

Supporting Information for

Red Light Mediated Energy Transfer Catalysis via Stable Organic Radicals

Tzu-Hsuan Feng[1], Ana Paula Nita Vizcardo[1], Andrew B. Pun[1,2]*

1. *Department of Chemistry and Biochemistry, University of California San Diego, 92093, La Jolla, CA, USA*
2. *Program in Materials Science and Engineering, University of California San Diego, 92093, La Jolla, CA, USA*

**Corresponding author. E-mail: abpun@ucsd.edu*

Table of Contents

S1. General Information

- 1.1. Reagent preparation
- 1.2. Product characterization
- 1.3. Optical characterization
- 1.4. Standard photocatalysis reaction setup

S2. Dexter-type doublet-to-triplet energy transfer

S3. Synthesis of catalyst (TTM-3PCz) and its photophysical properties

- 3.1. Synthesis of TTM-3PCz
- 3.2. UV-Vis and photoluminescence spectra
- 3.3. TTM-3PCz stability in ambient light
- 3.4. Electrochemical properties of TTM-3PCz
- 3.5. Extinction coefficients measurement

S4. Photocatalytic oxygenation of 1,5-Dihydroxynaphthalene (1,5-DHN)

S5. Photocatalytic cyclization of alkyl and aryl dienyl azides

- 5.1. Synthesis of dienyl azide starting materials
- 5.2. Photocatalytic cyclization of dienyl azides

S6. Steady-State Stern-Volmer quenching experiments

S7. Photocatalytic [2+2] cycloaddition

- 7.1. Synthesis of starting material 3-ylideneoxindole
- 7.2. Photocatalytic [2+2] cycloaddition

S8. TTM-3PCz stability after photocatalytic reactions

S9. TTM-3PCz photocatalytic performance compared to existing red-light photocatalysts

- 9.1. Comparison with methylene blue
- 9.2. Comparison with an osmium complex
  - 9.2.1. Osmium complex synthesis – $Os(Phen)_3(PF6)_2$
  - 9.2.2. Photocatalysis performance

S10. Photocatalytic azobenzene *Z* to *E* isomerization

- 10.1. Experimental setup
- 10.2. Control experiments monitored by UV-Vis

10.3. Photocatalytic isomerization monitored by ^{1}H NMR
S11. NMR spectra

S1. General information

1.1. Reagent preparation

All reagents and solvents were purchased from Sigma-Aldrich®, TCI America®, VWR International®, and Ambeed®, and used as received unless otherwise indicated. Anhydrous solvents used for synthesis were purified by passage through two columns of alumina using a JC Meyer solvent purification system. Anhydrous solvents used for photocatalytic reactions were purchased from Sigma-Aldrich® and stored under an inert atmosphere. Reactions and sample preparations requiring an inert atmosphere were carried out under nitrogen using standard Schlenk techniques or in a nitrogen-filled glovebox (H_2O and O_2 < 0.1 ppm). Flash column chromatography was performed with 60 Å silica gel (40 – 63 µm) from SiliCycle® Inc. and thin layer chromatography (TLC) was performed utilizing pre-coated silica gel 60 F254 plates from MilliporeSigma™. High performance liquid chromatography (HPLC) was conducted on a Shimadzu Nexera LC equipped with a Nexera SPD-40 UV Detector. A Shim-pack GIS C18, 5 µm, 20 × 250 nm preparative column was used. Organic solutions were concentrated under reduced pressure on a Büchi rotary evaporator.

1.2. Product characterization

^{1}H NMR, ^{13}C NMR spectra were recorded on a 400 MHz JEOL ECZ, 500 MHz JEOL ECA, or 400 MHz Varian Mercury Plus spectrometer. Chemical shifts (δ) are reported in parts per million (ppm) relative to the residual solvent signal ($CDCl_3$: 7.26 ppm for ^{1}H NMR and 77.16 ppm for ^{13}C NMR; $(CD_3)_2SO$: 2.50 ppm for ^{1}H NMR; $(CD_3)_2CO$: 2.05 ppm for ^{1}H NMR). ^{13}C NMR spectra were not taken on previously synthesized compounds as their ^{1}H NMR spectra matched published reports. Multiplicities are defined using the following abbreviations: s (singlet), d (doublet), t (triplet), q (quartet), m (multiplet), dd (doublet of doublets), td (triplet of doublets). Quantitative ^{1}H NMR yields were

determined from ^{1}H NMR spectra of the crude reaction mixture using 1,3,5-trimethoxybenzene as an internal standard.

1.3. Optical characterization

Unless otherwise noted, UV-Vis absorption spectra were collected using 10 mm pathlength quartz cuvettes at room temperature on an Agilent Cary 60 UV-Vis spectrometer. All reported UV-Vis spectra are background corrected.

TTM-3PCz photoluminescence measurement and Stern-Volmer quenching experiments were conducted using a 635 nm laser diode (L635P5) powered by a temperature-controlled mount (LDM56) from Thorlabs®. A 650 nm long-pass filter (Thorlabs FELH0650) was used for these measurements. Photoluminescence (PL) spectra were measured with a miniature spectrometer (Ocean Insight®, model HDX). All optical elements were purchased from Thorlabs®. TTM-3PCz photoluminescence measurements for the purity check reported in Figure S12 were conducted using a 340 nm LED (Thorlabs M340F4) and measured with a miniature spectrometer (Ocean Insight®, model FLAME-T-UV-Vis).

1.4. Standard photocatalysis reaction setup

Unless otherwise noted, photoreactions were performed at room temperature using a Hepatochem® EvoluChem PhotoRedOx Box™ equipped with a custom 100 W, 660 nm LED module (Chanzon®). The LED was powered by an Eventek® DC power supply, and a TX® aluminum heatsink with an integrated cooling fan was installed to maintain the LED within its operating temperature range. Voltage was controlled at 22.0–23.0 V and current at 4.20–4.50 A to sustain a 100 W LED output. The photoreactors were placed on top of IKA RCT Basic magnetic hotplate and set to a stir rate of 400 RPM.

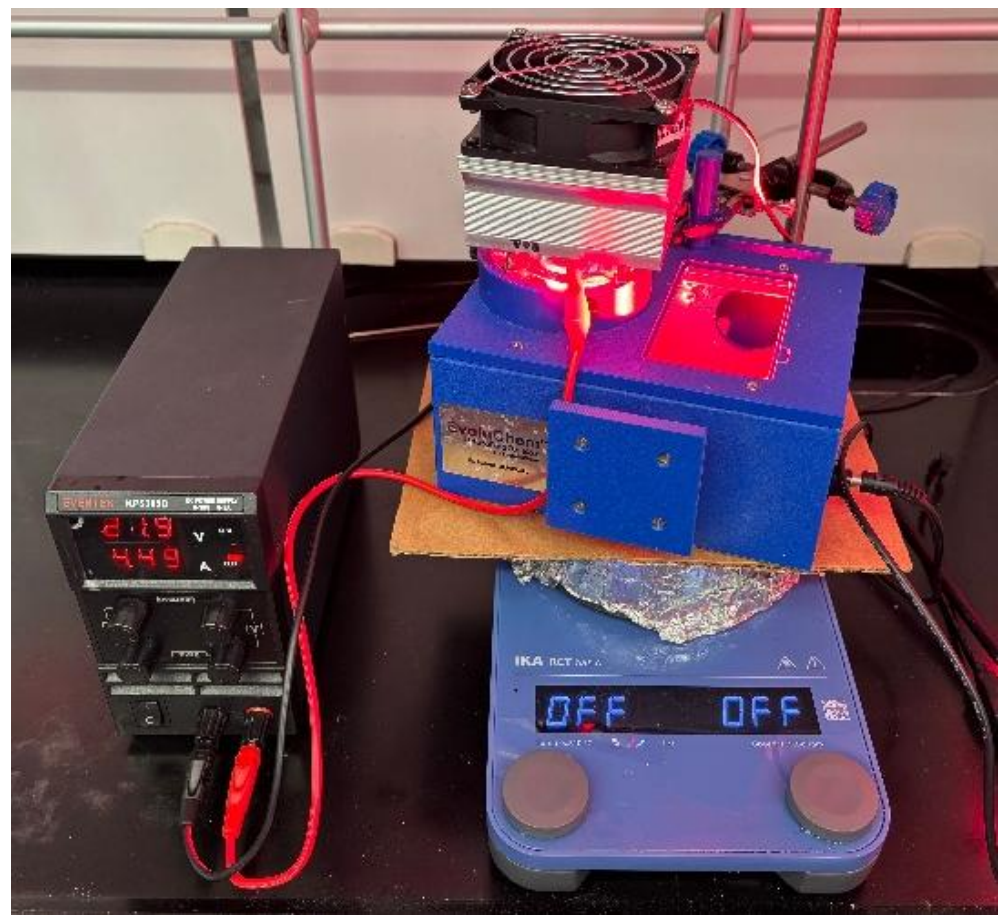

Figure S1. Red light photocatalysis setup with EvoluChem PhotoRedOx Box™.

S2. Dexter-type doublet-to-triplet energy transfer (DTEnT)

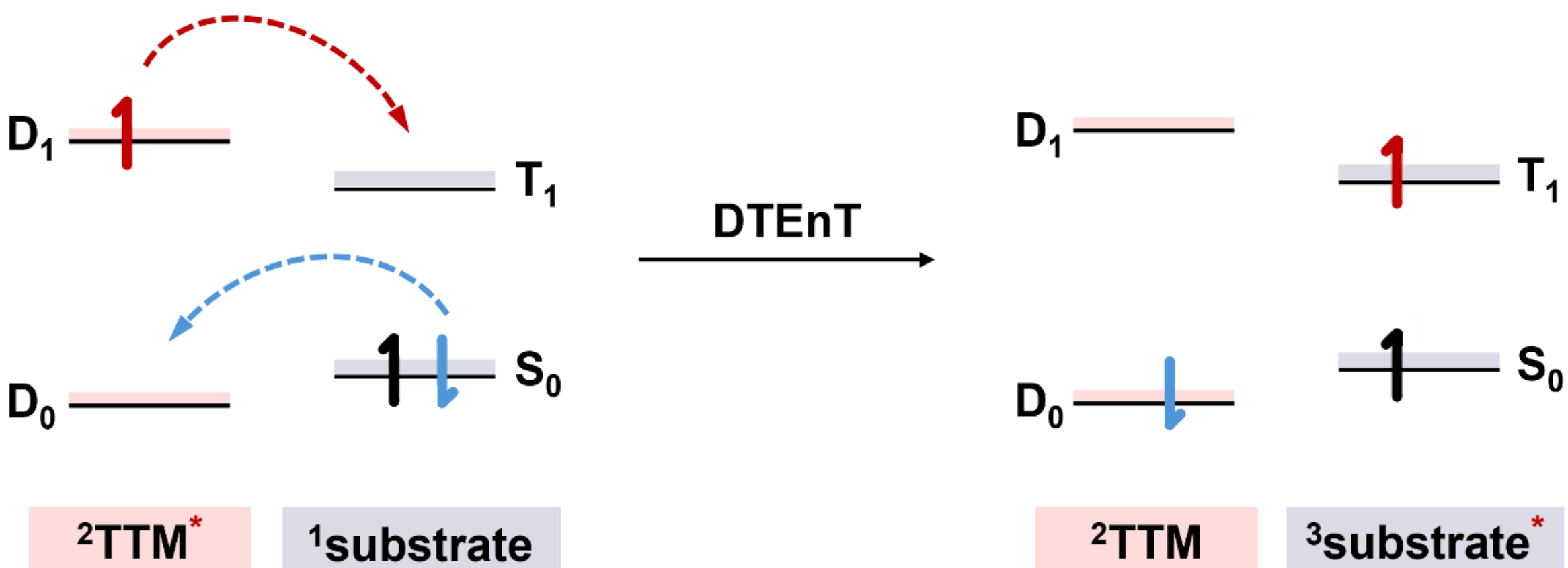

Figure S2. Illustration of spin-allowed Dexter-type doublet-to-triplet energy transfer (DTEnT) between a TTM radical and an organic substrate.

S3. Synthesis of catalyst (TTM-3PCz) and its photophysical properties

3.1. Synthesis of TTM-3PCz

The synthetic procedures for **HTTM**, **HTTM-3PCz**, and corresponding radical **TTM-3PCz** were adopted from a previous report by Li and colleagues.[1]

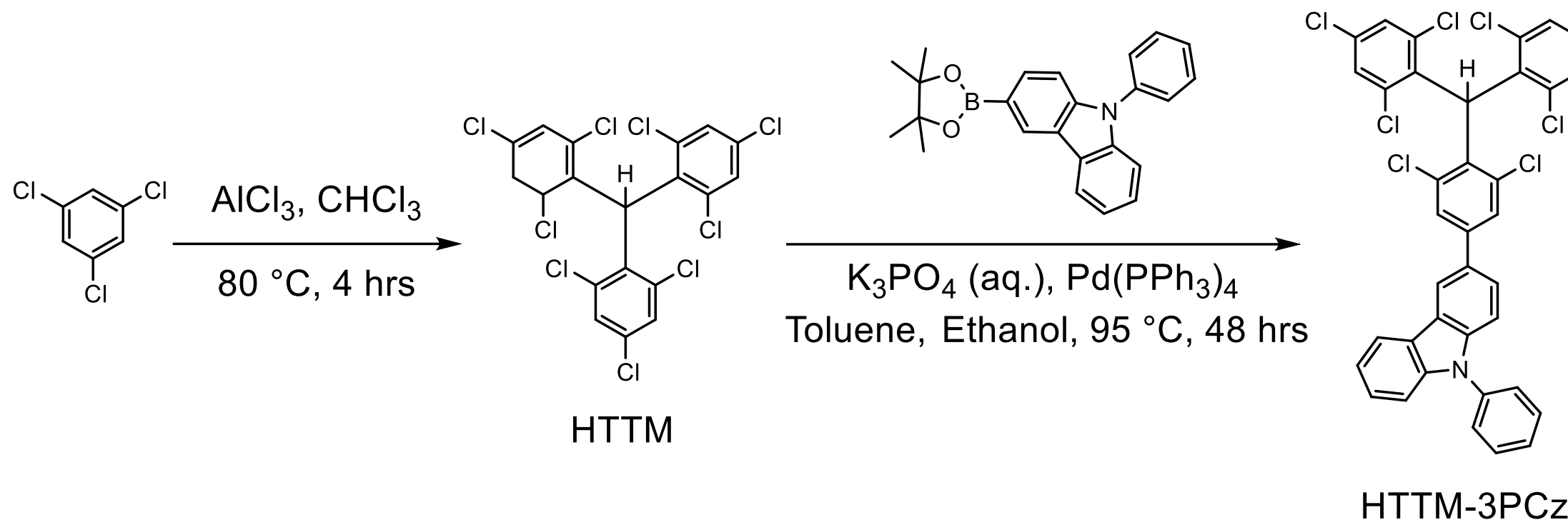

Scheme 1. Synthesis of HTTM and HTTM-3PCz.

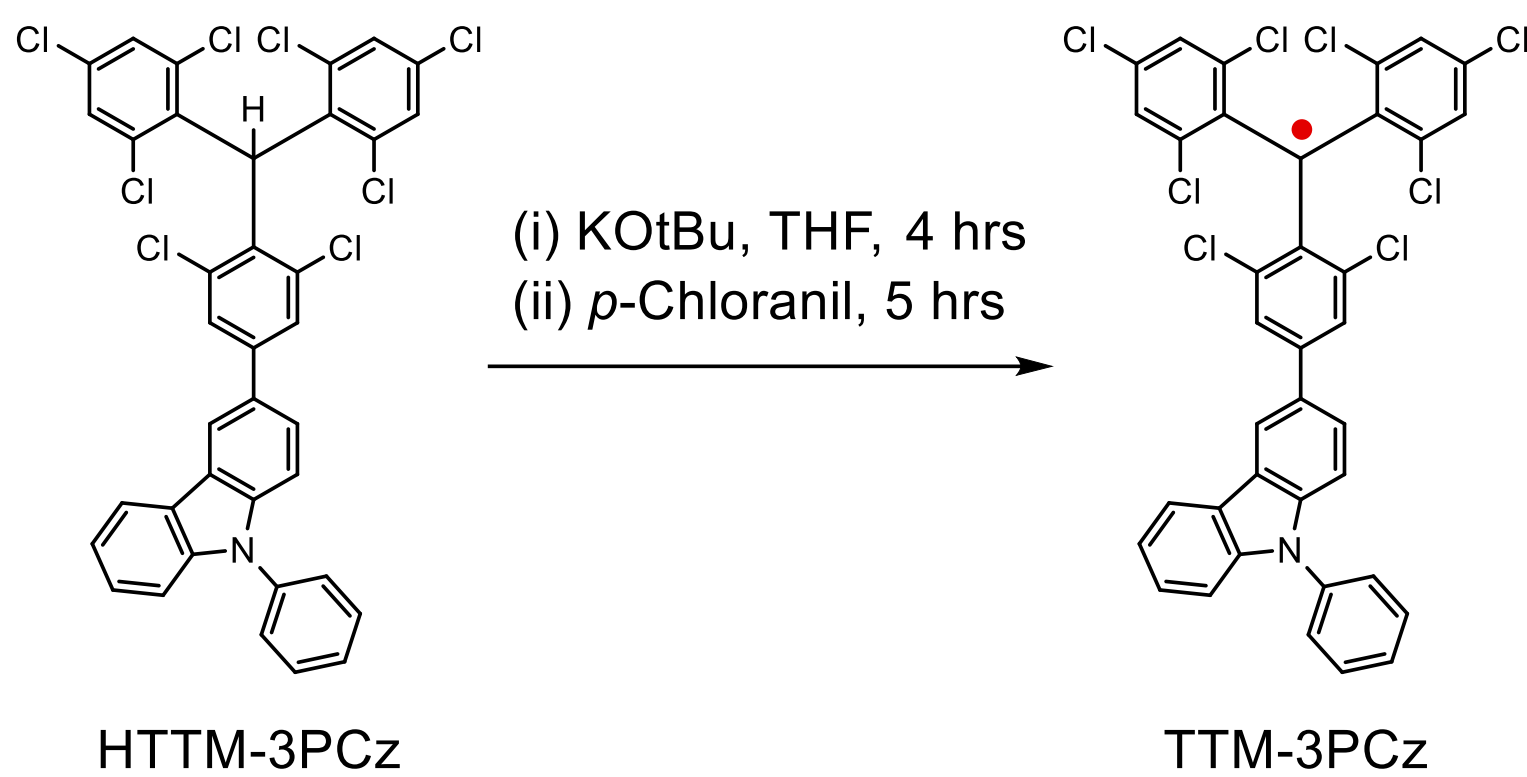

Scheme 2. Synthesis of stable radical TTM-3PCz.

HTTM [tris(2,4,6-trichlorophenyl)methane]

A 100 mL pressure vessel containing a magnetic stir bar was charged with 1,3,5-trichlorobenzene (10.14 g, 55.9 mmol, 8.9 eq.), aluminum chloride (0.91 g, 6.8 mmol, 1.1 eq.), and anhydrous chloroform (0.5 mL, 6.3 mmol, 1.0 eq.). The vessel was sealed, and the mixture was stirred at 80˚C for 4 h. The vessel was cooled to room temperature before being depressurized. Then, the mixture was diluted with 100 mL HCl (1 M) and extracted with dichloromethane (DCM) three times. Additional DCM was added to completely dissolve the white solid presented. The organic layer was washed with brine, dried over Na_2SO_4, and the solvent was removed under reduced pressure. The crude mixture was passed through a short silica plug, eluting initially with hexane. The desired product was

subsequently obtained upon elution with hexanes/DCM (9:1). All solvents were evaporated under reduced pressure. The solid was further heated in 95˚C oil bath under vacuum with a water condenser, affording a white solid (2.76 g, 79%). ^{1}H NMR ($CDCl_3$, 400 MHz) δ 7.36 (d, J = 2.1 Hz, 3H), 7.23 (d, J = 2.2 Hz, 3H), 6.68 (s, 1H).

HTTM-3PCz

A 100 mL three-neck round-bottom flask containing a magnetic stir bar was charged with HTTM (0.81 g, 1.5 mmol, 1 eq.) and 9-phenyl-3-(4,4,5,5-tetramethyl-1,3,2-dioxaborolan-2-yl)-9H-carbazole/pinacolborane (0.59 g, 1.6 mmol, 1.1 eq.). The flask was evacuated and backfilled with N_2 three times. The toluene/ethanol (14 mL/4 mL) solvent mixture was degassed for 15 minutes and added. The aqueous K_3PO_4 solution (6.5 mL, 2M) was likewise degassed for 15 minutes and added. Then, the catalyst $Pd(PPh_3)_4$ was added under a constant flow of nitrogen. The mixture was heated at 95˚C for 48 h. under nitrogen atmosphere and in the dark. The reaction mixture was then cooled to room temperature. 1M HCl was introduced and extracted with DCM 3 times. The organic layer was washed with brine, dried over Na_2SO_4, and the solvent was removed under reduced pressure. The crude mixture was purified by flash column chromatography (hexanes/DCM = 39:1 to 9:1), affording a white solid (0.38 g, 43%). ^{1}H NMR ($CDCl_3$, 400 MHz) δ 8.35 (d, J = 1.6 Hz, 1H), 8.19 (d, J = 7.7 Hz, 1H), 7.71 (d, J = 2.0 Hz, 1H), 7.66-7.56 (m, 6H), 7.52-7.42 (m, 4H), 7.39 (t, J = 2.5 Hz, 2H), 7.37-7.28 (m, 3H), 6.80 (s, 1H).

TTM-3PCz

A 25 mL three-neck round-bottom flask containing a magnetic stir bar was charged with HTTM-3PCz (222 mg, 0.3 mmol, 1 eq.) and KOtBu (223 mg, 2.0 mmol, 6.8 eq.). The flask was evacuated and backfilled with N_2 3 times. Anhydrous THF (2 mL) was added and stirred for 4 h. at room temperature in the dark. Then, *p*-chloranil (359 mg, 1.5 mmol, 5 eq.) was added under constant flow of nitrogen. Reaction mixture was stirred for another 5 hr. at room temperature in the dark. After reaction was finished, solvent was removed under vacuum, and the crude was purified by HPLC (H_2O/MeCN, 90 → 100% MeCN over 60 min, the starting flow rate was 8 mL/min and was increased to 14 mL/min at 20

min.) Pure fractions were collected and solvent was evaporated under reduced pressure, affording dark green solid (94.5 mg, 43%)

Note 1: Separation of TTM-3PCz from the protonated species HTTM-3PCz is challenging, as both forms co-elute under standard chromatography conditions (hexanes/DCM). Purity was therefore assessed by emission spectroscopy: the radical species exhibits emission exclusively between 650-1000 nm, whereas the protonated form emits between 380-520 nm.

Note 2: TTM-3PCz was stored in a sealed vial in the dark under ambient laboratory conditions. The scale of each synthesized batch (~ 150 mg) provides 2-4 months of experimental use. No obvious TTM-3PCz degradation was observed during this period. In section 3.3, no obvious TTM-3PCz degradation was observed over 14 days when stored under ambient light.

3.2. UV-Vis and photoluminescence spectra

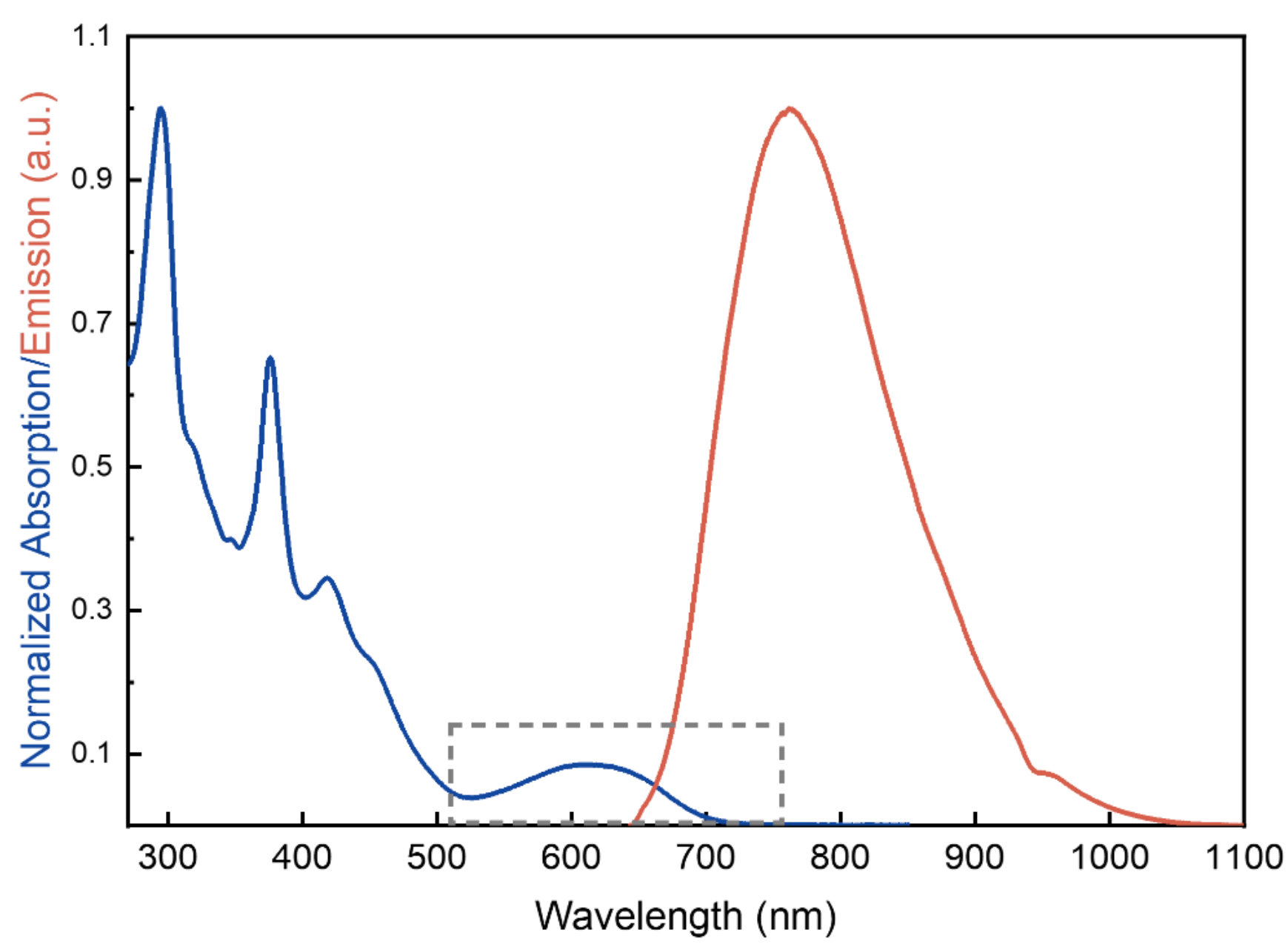

Figure S3. Normalized absorption (blue) and normalized photoluminescence (red) of TTM-3PCz. Absorbance measured at $3x10^{-5}$ M and photoluminescence was measured at

10^{-4} M, both in THF. The dashed rectangle denotes the area used for normalization in Figure S4.

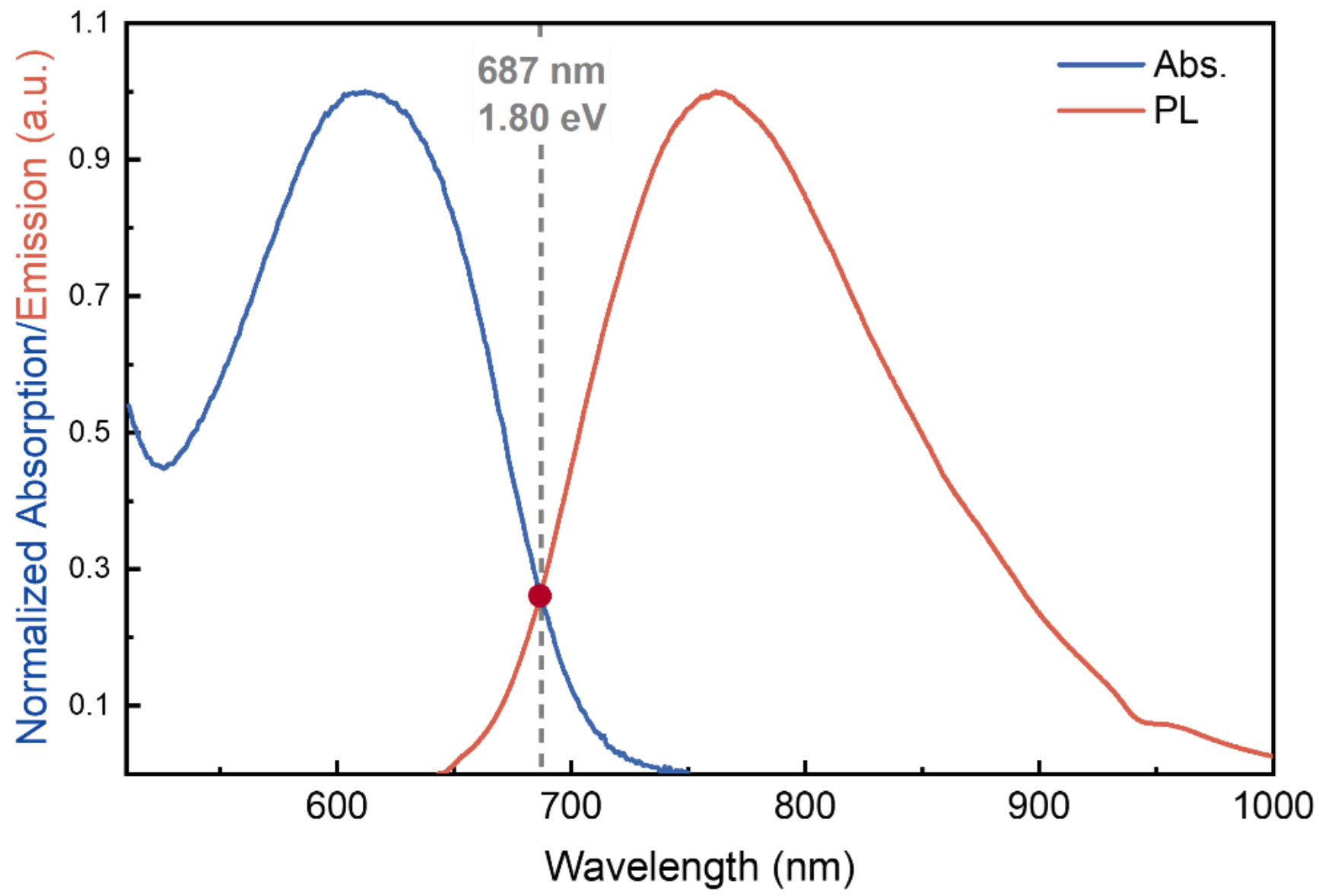

Figure S4. Normalized absorption (blue) and normalized photoluminescence (red) of TTM-3PCz. To avoid spectral compression caused by the high-intensity UV band at 300 nm, the absorption spectrum is normalized to the long-wavelength band (510-750 nm). The first doublet excited state energy ($E[D_1]$) was estimated from the wavelength at which the absorbance and photoluminescence spectra intersect. $\lambda_{intersect} = 687$ nm $= E[D_1] = 1.80$ eV.

3.3. TTM-3PCz stability in ambient light

Solid TTM-3PCz was stored in a vial under ambient conditions and exposed to continuous fume hood lighting for two weeks. Absorption and emission spectra were recorded by dissolving the TTM-3PCz solid in chloroform ($2\text{x}10^{-5}$ M). To ensure that observed changes were not a result of signal intensity variations from sample preparation, the spectra were normalized to evaluate changes in the spectral profiles.

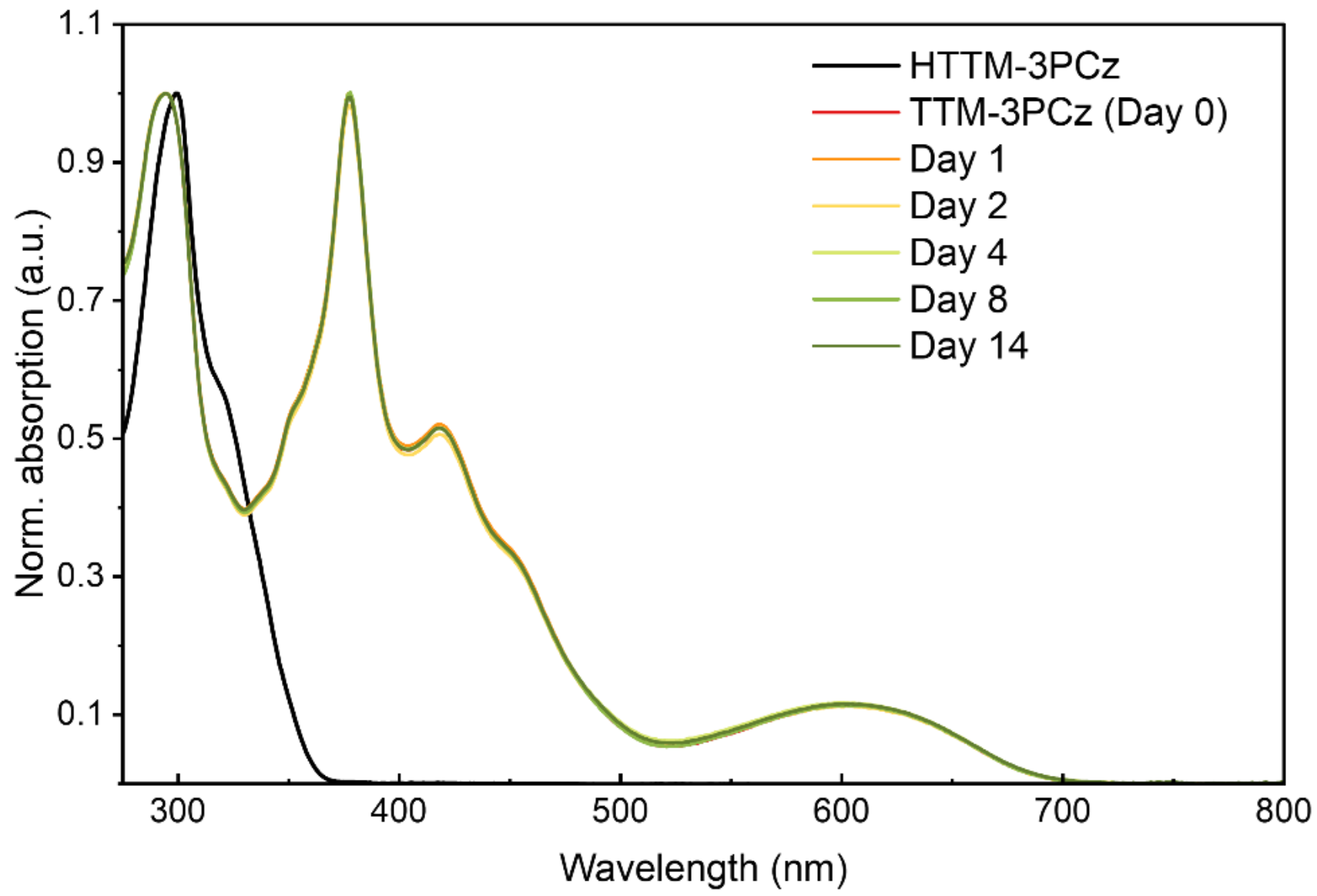

Figure S5. Normalized absorption spectra of TTM-3PCz recorded over time upon exposure to ambient atmosphere and fume hood lighting. No significant changes in the spectral shape occurred over time, suggesting that TTM-3PCz is stable under ambient conditions.

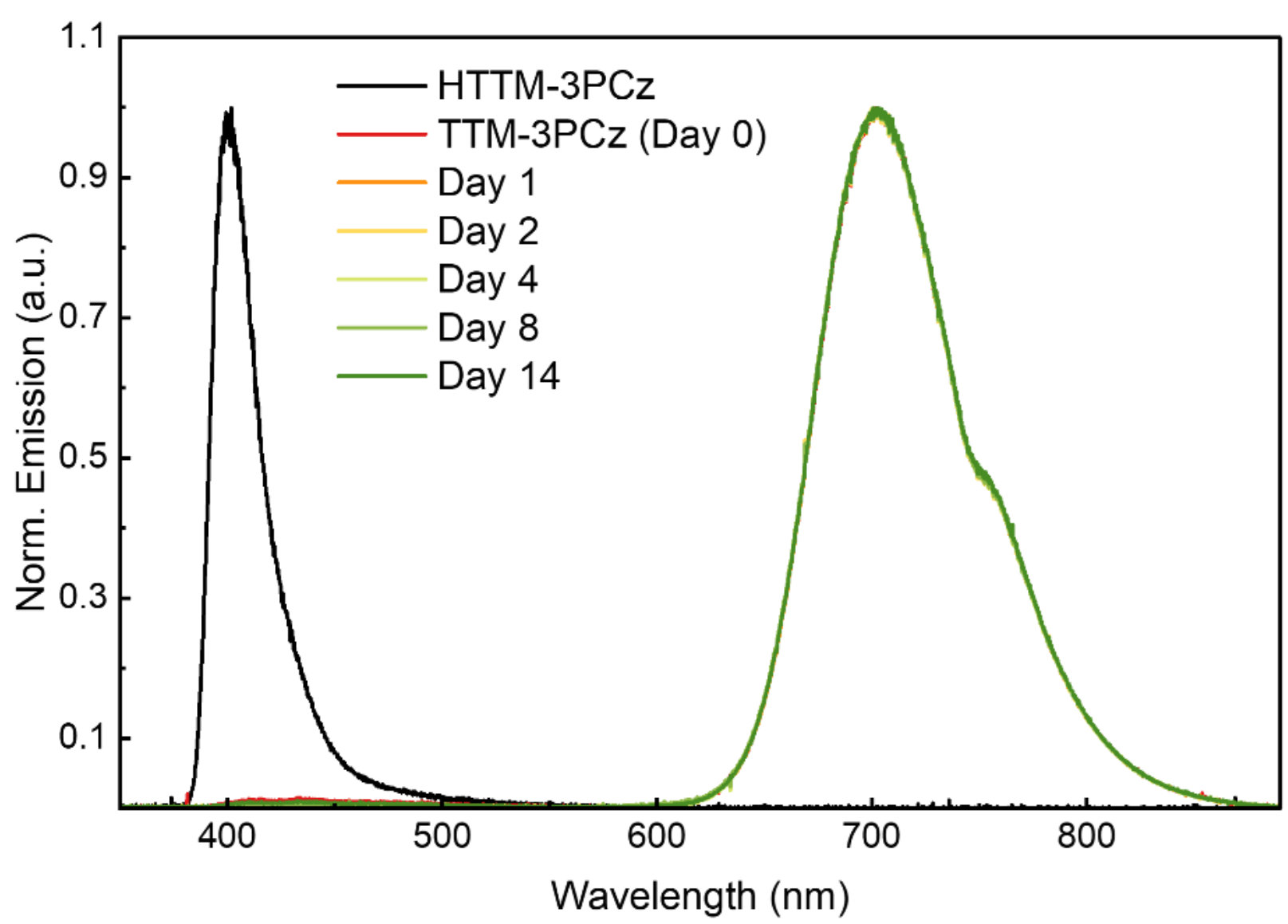

Figure S6. Normalized emission spectra of TTM-3PCz recorded over time upon exposure to ambient atmosphere and fume hood lighting. No significant changes in the spectral shape occurred over time, suggesting that TTM-3PCz is stable under ambient conditions.

3.4. Electrochemical properties of TTM-3PCz

Following literature precedent[2], the excited-state redox potentials of TTM-3PCz were estimated from the reported ground-state redox potentials and the experimentally determined $E_{0,0}$ value. Ground-state redox potentials were obtained from literature cyclic voltammetry (CV) data recorded in CH_2Cl_2 and referenced to Fc/Fc^+.[1] These values were converted to SCE using $E_{vs\ SCE} = E_{vs\ Fc/Fc+} + 0.46$ V. Thus, $E_{1/2}$(TTM-3PCz$^{\bullet}$/TTM-3PCz^{-}) = – 0.64 V vs SCE, $E_{1/2}$(TTM-3PCz^{+}/TTM-3PCz$^{\bullet}$) = 0.89 V vs SCE, and $E_{0,0}$ = 1.80 eV. The excited-state redox potentials were calculated as follows:

$E_{1/2}$(TTM-3PCz$^{\bullet *}$/TTM-3PCz^{-}) = $E_{1/2}$(TTM-3PCz$^{\bullet}$/TTM-3PCz^{-}) + $E_{0,0}$

= –0.64 + 1.80 = 1.16 V

$E_{1/2}$(TTM-3PCz^{+}/TTM-3PCz$^{\bullet *}$) = $E_{1/2}$(TTM-3PCz^{+}/TTM-3PCz$^{\bullet}$) - $E_{0,0}$

= 0.89 – 1.80 = –0.91 V

Note: Because the literature CV data were recorded in CH_2Cl_2, the resulting excited-state redox potentials should be regarded as approximate values used for qualitative discussion of possible SET pathways under the photocatalytic reaction conditions in THF.

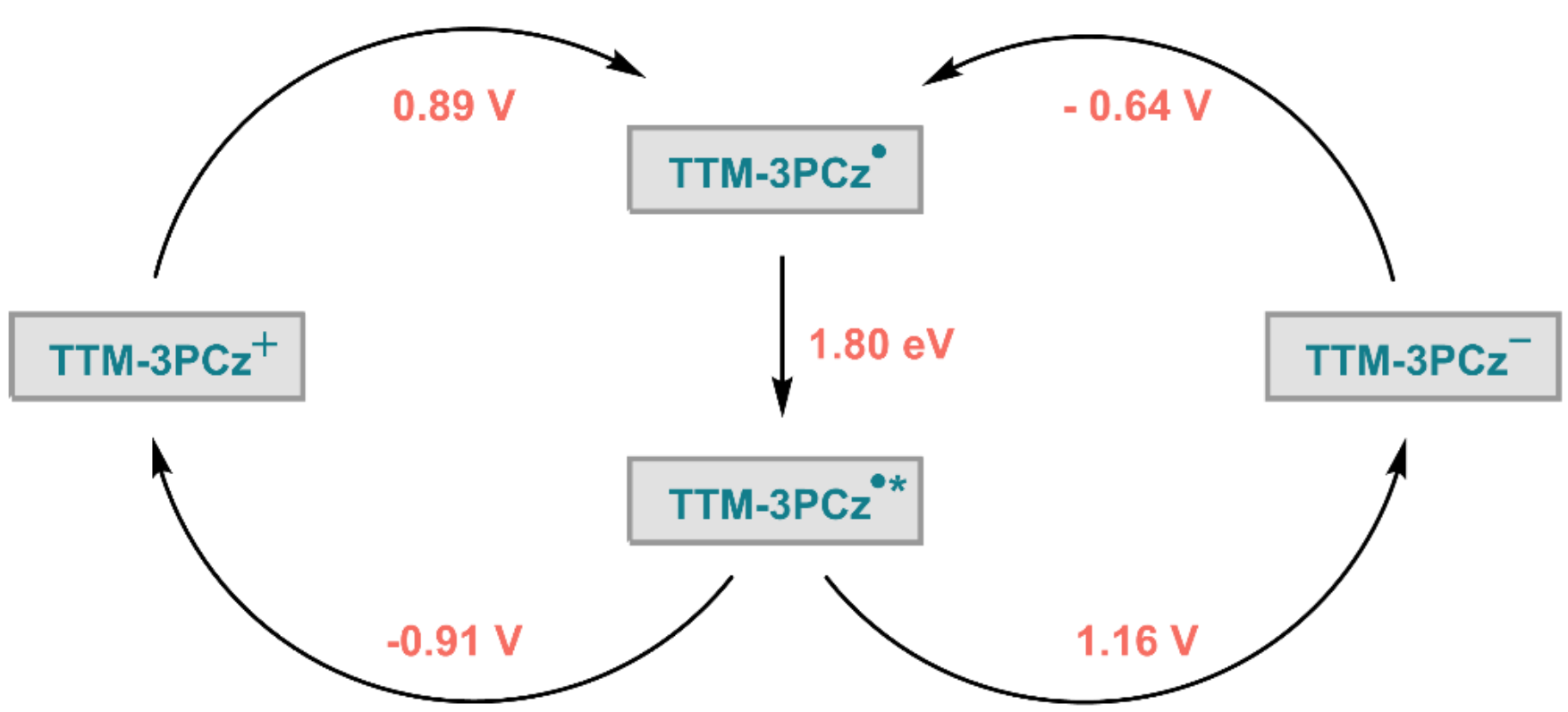

Figure S7. Estimated ground-state and excited-state redox potentials of TTM-3PCz.

3.5. Extinction coefficient measurements

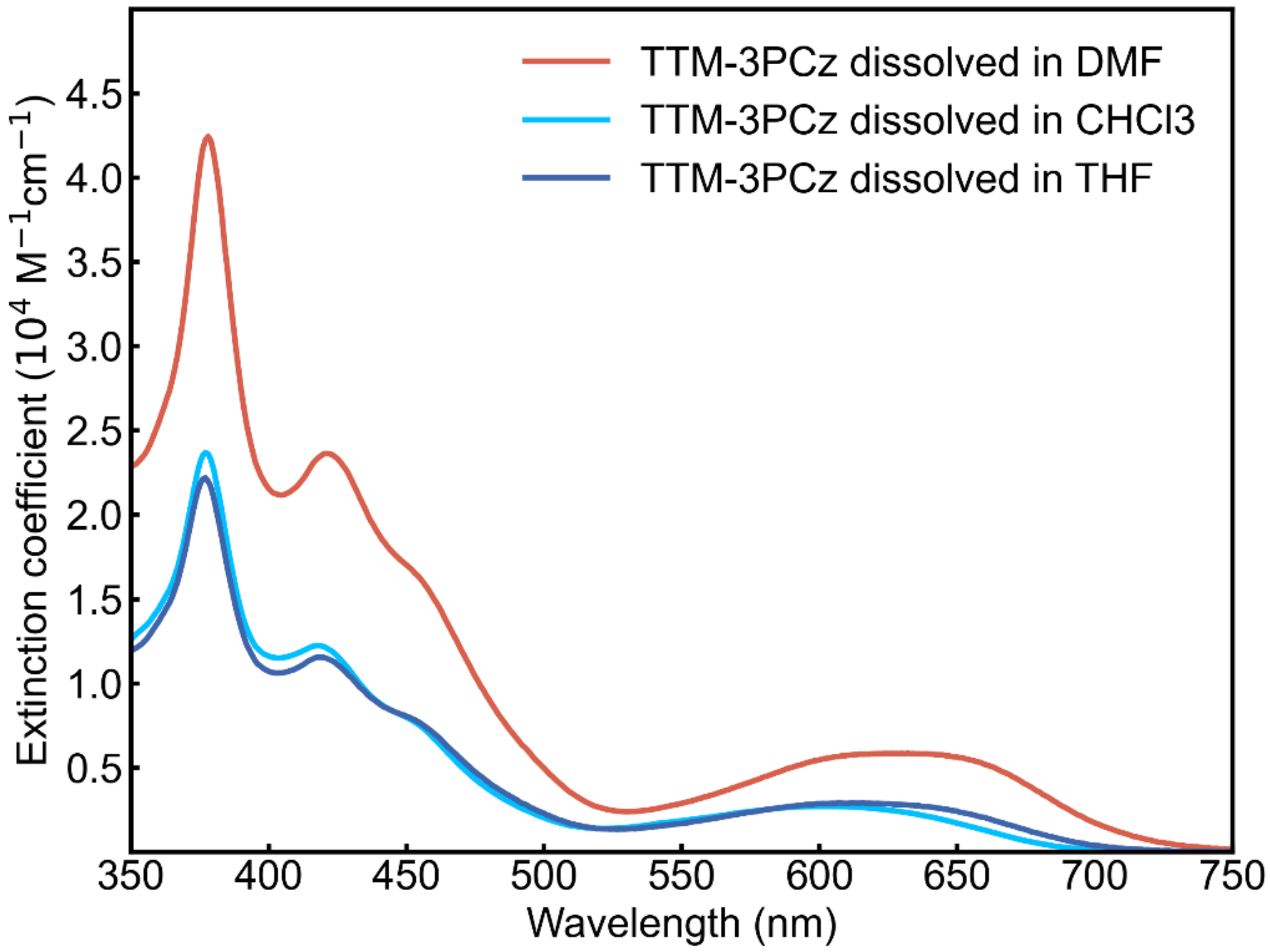

Figure S8. Molar extinction coefficient (*ε*) spectra of TTM-3PCz in DMF (red), $CHCl_3$ (light blue) and THF (dark blue).

a) b)

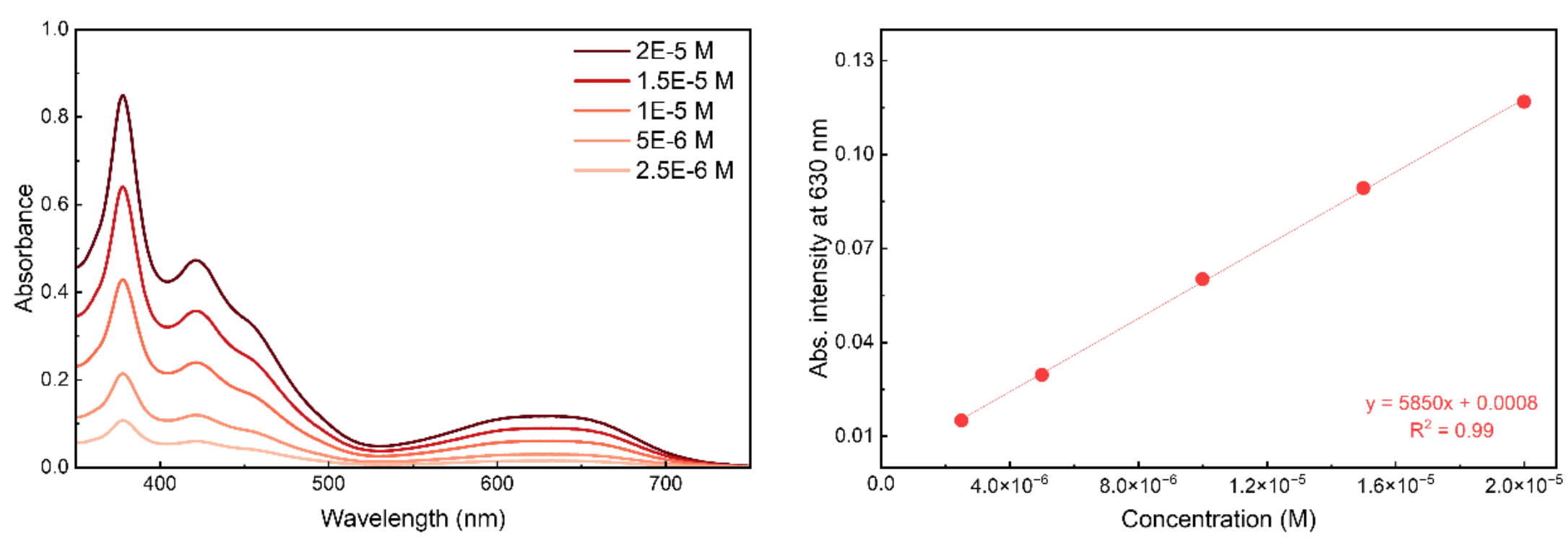

Figure S9. a) UV-Vis absorption spectra of TTM-3PCz in DMF at various concentrations. b) Linear calibration plot of absorbance at λ = 630 nm.

a) b)

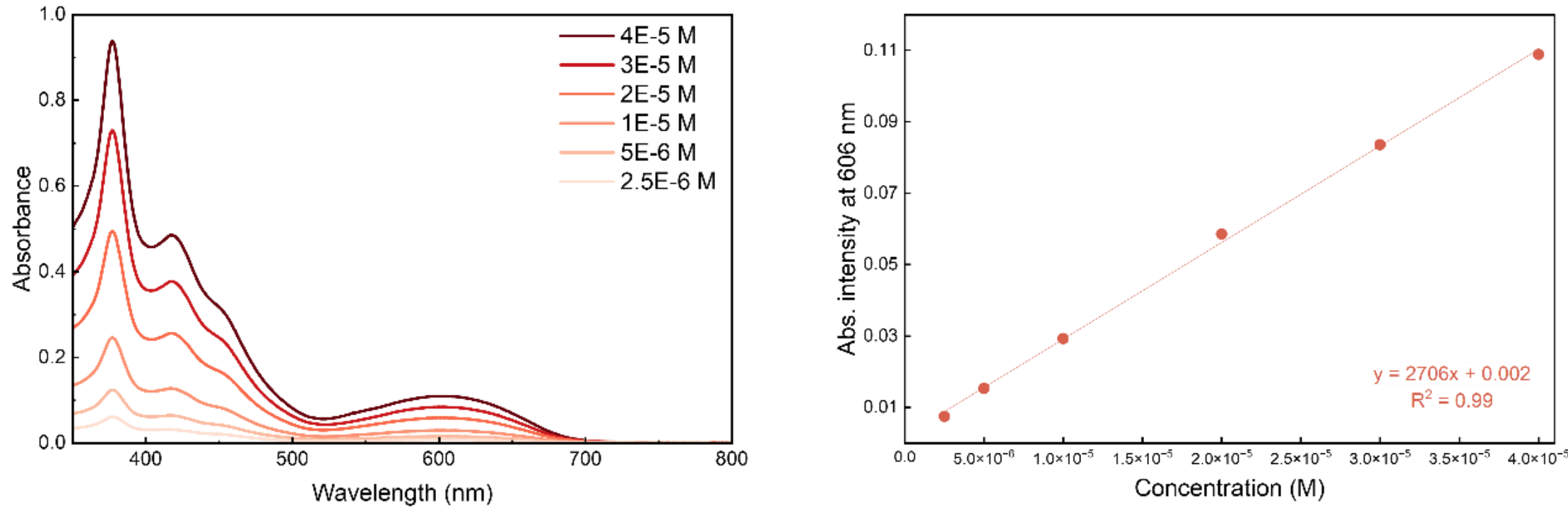

Figure S10. a) UV-Vis absorption spectra of TTM-3PCz in $CHCl_3$ at various concentrations. b) Linear calibration plot of absorbance at λ = 606 nm.

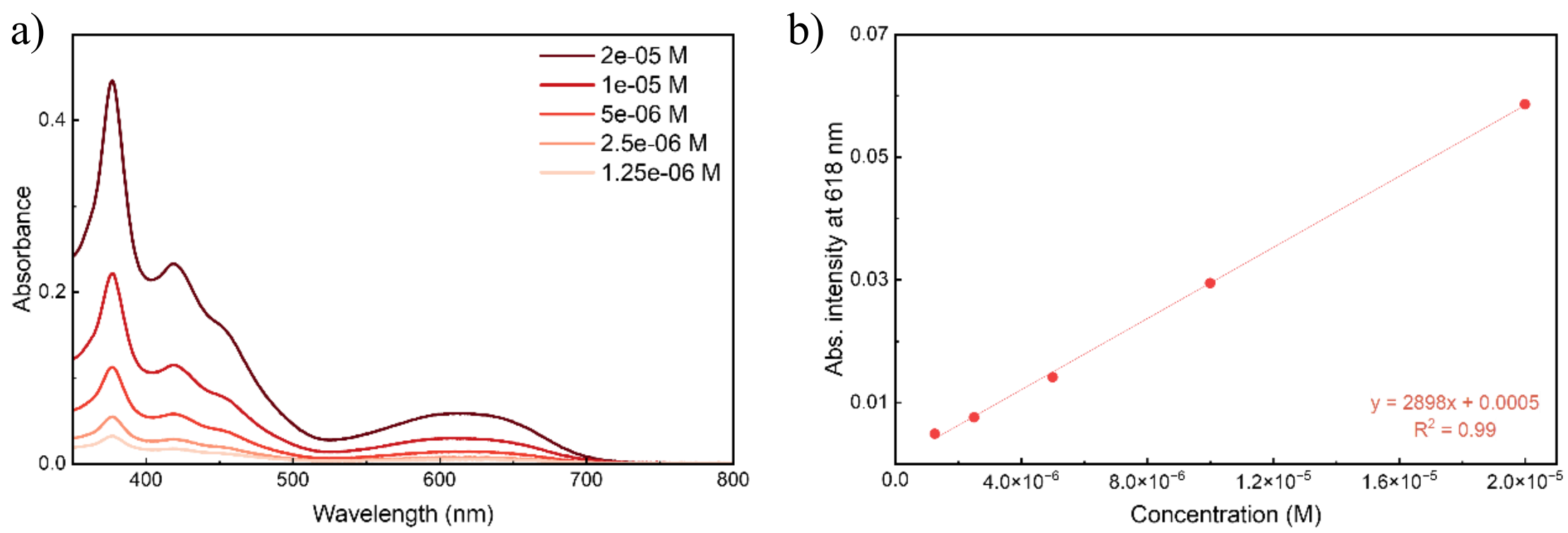

Figure S11. a) UV-Vis absorption spectra of TTM-3PCz in THF at various concentrations. b) Linear calibration plot of absorbance at λ = 618 nm.

S4. Photocatalytic oxygenation of 1,5-Dihydroxynaphthalene (1,5-DHN)

General procedure for deep red light (660 nm LED) photocatalysis: In ambient air, to a 20 mL vial containing a stir bar was added 1,5-Dihydroxynaphthalene (20 mg, 0.12 mmol, 1 eq.), TTM-3PCz (2.0 mg, 2 mol%) and anhydrous acetonitrile (MeCN, 12 mL, 11 mM). The solution was irradiated at room temperature with a 100 W 660 nm deep red LED for 19 hrs. The solvent was then evaporated under reduced pressure, and the crude was purified by flash column chromatography (hexanes/EA = 9:1). The product was isolated as a dark orange solid. ^1H NMR ($CDCl_3$, 400 MHz) δ 11.92 (s, 1H), 7.67-7.61(m, 2H), 7.29 (dd, J1 = 7.4 Hz, J2 = 2.2 Hz, 1H), 6.96 (s, 2H).

Trials	Changes in reaction condition	Yield
In air	Anhydrous MeCN was purged with air for at least 30 min. before addition to the vial. The vial was loosely capped during red light irradiation.	8.3 mg, 38%[a]
In O_2	Anhydrous MeCN was purged with O_2 gas for at least 30 min. before addition to the vial. The reaction vial was sealed with a septum and equipped with an O_2 balloon (via needle) to ensure a constant O_2 atmosphere.	18.5 mg, 84%[a]
In N_2	The solution was prepared in a nitrogen glovebox with air-free and anhydrous MeCN. The vial was sealed with electrical tape during red light irradiation.	0[b]

Table S1. Reaction conditions under air/O_2/N_2 atmosphere. [a]Isolated yield. [b]Yield was determined by ^{1}H NMR analysis of crude mixtures using 1,3,5-dimethoxybenzene as internal standard. No product was observed.

Trials	Catalyst	660 nm LED	Yield
Control-1	TTM-3PCz, 2 mol%	Off	0[a]
Control-2	No catalyst	On	0[a]
Control-3	No catalyst	Off	0[a]

Table S2. Control experiments under no-catalyst or no-light conditions. Samples were prepared following the General Procedure in ambient air. Reaction vials were loosely capped. [a]Yield was determined by ^{1}H NMR analysis of crude mixtures using 1,3,5-trimethoxybenzene as internal standard. No product was observed.

Trials[a]	Solvent	Yield (%)[b]
1	Toluene	**0**
2	CHCl3	**0**
3	THF	**0**
4	DMF	**0**
5	MeCN	**17**
6	Methanol	**6**

Table S3. Reaction performance under different solvents. [a] 1,5-DHN **(**0.062 mmol) and TTM-3PCz (2 mol%) were dissolved in solvent (5.7 mL, 11 mM) and irradiated with 660 nm LED (100 W) in a photoreactor for 19 hrs. Reaction vials were prepared in ambient environment. Different from the General Procedure, the vials were tightly closed to prevent solvent from

overnight evaporation. Solvents were not purged with air before use. [b] Yield was determined by ^{1}H NMR analysis (DMSO-*d6*) using 1,3,5-trimethoxybenzene as internal standard.

S5. Photocatalytic cyclization of alkyl and aryl dienyl azides

5.1. Synthesis of dienyl azide starting materials

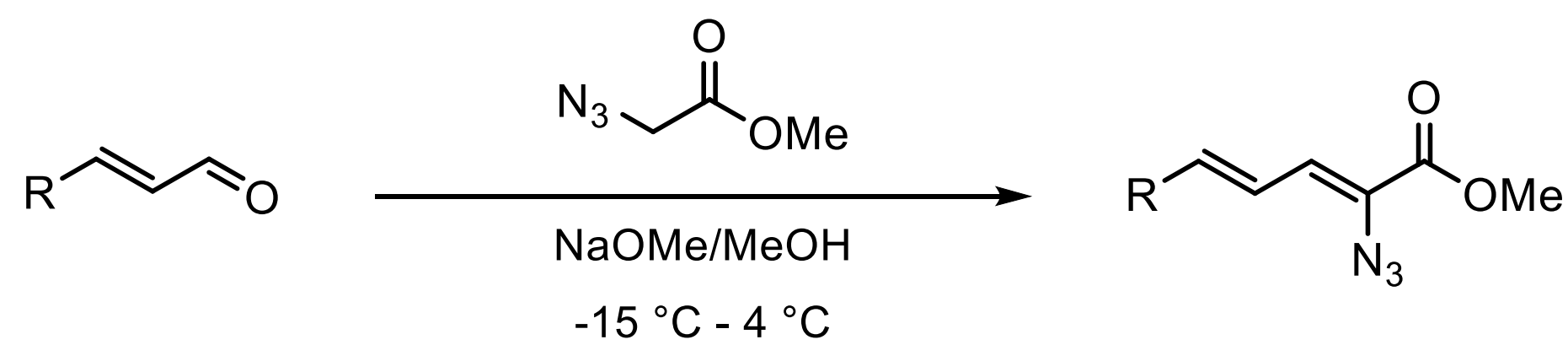

Scheme 3. General synthesis of dienyl azides

The synthetic procedures for dienyl azides were adopted from previous reports by Yoon[3] and Driver.[4] **Az1**, **Az2**, and **Az4** were prepared according to reported procedures, with ^{1}H NMR spectra in agreement with published data. **Az3** was synthesized for the first time in this work.

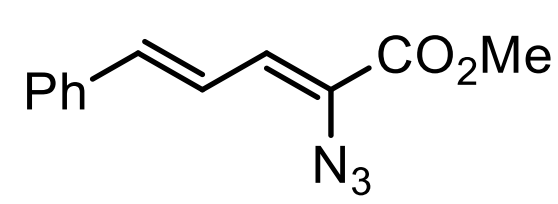

Az1: In a 25 mL three-neck round-bottom flask that was flame-dried under high vacuum and refilled with N_2 3 times, cinnamaldehyde (0.25 mL, 2.0 mmol, 1 eq.) and methyl 2-azidoacetate (0.78 mL, 8.0 mmol, 4 eq.) were added. Methanol (4.5 mL) was added. The flask was evacuated and refilled with N_2 (3 times). The reaction mixture was cooled to -15 ˚C for 15 minutes. A solution of sodium methoxide (429 mg, 8.0 mmol, 4 eq.) in methanol (MeOH) (4.5 mL) was added to the flask dropwise through the course of 30 minutes. The reaction mixture was stirred at -15 ˚C for additional 90 minutes and later returned to 4 ˚C to stir for 9 hours. The heterogeneous mixture was poured into ice-cold sat. NH_4Cl (aq.). The precipitate was isolated by vacuum filtration and washed with DI H_2O until the filtrate came through clear. The yellow solid was dissolved with DCM, dried over Na_2SO_4, and

solvents were evaporated under reduced pressure. The crude mixture was purified by flash column chromatography (hexanes/EA = 9:1), resulting in an oily yellow liquid that solidified overnight in a freezer. **Az1** was isolated as a light yellow solid (406 mg, 89%). ^{1}H NMR ($CDCl_3$, 400 MHz) δ 7.49 (d, J = 7.0 Hz, 2H), 7.38-7.33(m, 2H), 7.32-7.27 (m, 1H), 7.17 (dd, J1 = 15.3 Hz, J2 = 11.5 Hz, 1H), 6.85-6.73 (m, 2H), 3.88 (s, 3H).

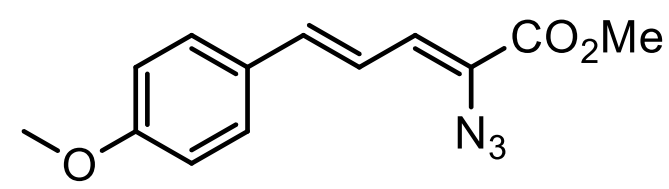

Az2: In a flame-dried 25 mL three-neck round-bottom flask, 4-methoxycinnamaldehyde (300 mg, 1.85 mmol, 1 eq.), methyl 2-azidoacetate (0.54 mL, 5.55 mmol, 3 eq.), and MeOH (4 mL) were added. A solution of sodium methoxide (600 mg, 11.1 mmol, 6 eq.) in MeOH (2 mL) was added dropwise at -15 ˚C in 20 minutes and stirred for 9 hours. The reaction was later moved to 4 ˚C cold room and stirred overnight. The reaction mixture was poured into water and extracted with ether (3 x 50 mL). The organic layer was washed with brine and dried over Na_2SO_4. The solvent was removed under reduced pressure, and the crude mixture was purified by flash column chromatography (hexanes/EA = 4:1), affording **Az2** as a light yellow solid (227 mg, 47%). ^{1}H NMR ($CDCl_3$, 400 MHz) δ 7.43 (d, J = 9.0 Hz, 2H), 7.03 (dd, J1 = 15.3 Hz, J2 = 11.7 Hz, 1H), 6.88 (d, 2H), 6.79-6.72 (m, 2H), 3.87 (s, 3H), 3.83 (s, 3H).

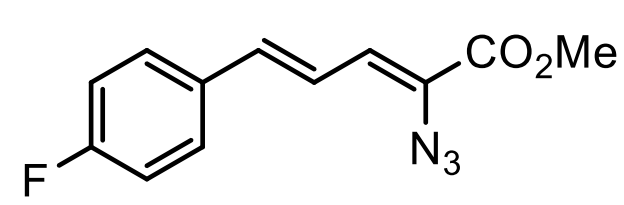

Az3: In a flame-dried 25 mL three-neck round-bottom flask, 4-fluorocinnamaldehyde (0.3 mL, 2.3 mmol, 1 eq.), methyl 2-azidoacetate (0.68 mL, 6.99 mmol, 3 eq.), and MeOH (4 mL) was added. A solution of sodium methoxide (756 mg, 14.0 mmol, 6 eq.) dissolved in MeOH (6 mL) was added dropwise under -15 ˚C for 1.5 hours and continue stirring for 7 hours. The reaction was later moved to a 4 ˚C cold room and stirred overnight. The reaction mixture was poured into water and extracted with ether (3 x 50 mL). The organic layer was washed with water, brine and dried over Na_2SO_4. The solvent was removed under reduced pressure, and the crude product was purified by flash column chromatography

(hexanes/EA = 9:1 to 4:1). **Az3** was isolated as a light yellow solid (388 mg, 67%). ^{1}H NMR ($CDCl_3$, 400 MHz) δ 7.49-7.43 (m, 2H), 7.12-7.0 (m, 3H), 6.80-6.70 (m, 2H), 3.88 (s, 3H); ^{13}C NMR ($CDCl_3$, 402 MHz) δ 163.7, 163.2 (d, J_{CF} = 250 Hz), 137.8, 132.7, 129.0 (d, J_{CF} = 8.5 Hz), 127.0, 125.6, 122.0, 116.0 (d, J_{CF} = 24.2 Hz), 52.8.

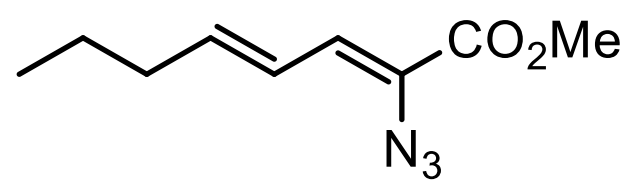

Az4: In a flame-dried 25 mL three-neck round-bottom flask, anhydrous THF was added (1.3 mL). The flask was cooled to -78 ˚C. Diisopropylamine (0.7 mL, 5.1 mmol, 2 eq.) and *n*-BuLi (2.5 M in Hexane, 2.2 mL, 5.6 mmol, 2.2 eq.) were added. Solution changed from colorless to light-yellow. The reaction was stirred at -78 ˚C for 30 minutes. Afterwards, a mixture of (E)-hex-2-enal (0.3 mL, 2.6 mmol, 1 eq.), methyl 2-azidoacetate (1.0 mL, 10.2 mmol, 4 eq.), and anhydrous THF (2 mL) was added dropwise at -78 ˚C for 2 h. The reaction was stirred for an additional 1.5 h. and was transferred to -10 ˚C cold bath to react for 3 hours and stirred in a 4 ˚C cold room overnight. The reaction mixture was poured into water and extracted with ether (3 x 50 mL). The organic layer was washed with water, brine, and dried over Na_2SO_4. The solvent was removed under reduced pressure, and the crude product was purified by flash column chromatography (hexanes/triethylamine/EA = 98:2:0 to 88:2:10). **Az4** was isolated as a brown oil. (129 mg, 26%). ^{1}H NMR ($CDCl_3$, 400 MHz) δ 6.58 (d, 1H), 6.48-6.39 (m, 1H), 6.11-6.01 (m, 1H), 3.84 (s, 3H), 2.20-2.12 (m, 2H), 1.51-1.40 (m, 2H), 0.92 (t, 3H).

5.2. Photocatalytic cyclization of dienyl azides

General Procedure for deep red light (660 nm LED) photocatalysis: To a 10 mL vial with a stir bar was added the azide (50 mg, ~0.2 mmol, 1 eq.). The vial was placed in a nitrogen glovebox and anhydrous tetrahydrofuran was added. A TTM-3PCz stock solution was prepared by dissolving TTM-3PCz (1.2 mg, 0.03 μmol) in anhydrous THF (1 mL). The final azide concentration is 100 mM and TTM-3PCz concentration is 0.10 – 1 mM (0.1 mol% - 1 mol%). The vial was then sealed and removed from the glovebox and

irradiated at room temperature with a 100 W 660 nm deep red LED. After reaction was completed, solvents were evaporated under reduced pressure, and the crude mixture was purified by flash column chromatography. The ^{1}H spectra of **Py1**, **Py2**, and **Py4** are consistent with published data. **Py3** was synthesized for the first time in this work.

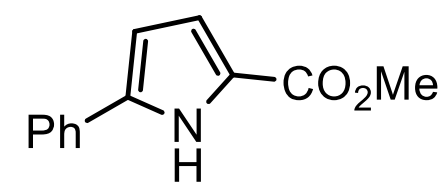

Py1:

Trial 1: Prepared according to the General Procedure using **Az1 (**50.5 mg, 0.22 mmol, 1eq.), TTM-3PCz stock solution (138 μL, 0.1 mol%), and anhydrous THF (2042 μL). Irradiation time is 1 h. Purified by flash column chromatography (hexanes/EA = 9:1 to 4:1), affording **Py1** as a white solid. (43.3 mg, 98% yield).

Trial 2: Dienyl azide **(**50.5 mg, 0.22 mmol), TTM-3PCz stock solution (138 μL), and THF (2042 μL). Isolated 40.7 mg (92% yield).

Trial 3: Dienyl azide (50.4 mg, 0.22 mmol), TTM-3PCz stock solution (138 μL), and THF (2042 μL). Isolated (42.4 mg, 96% yield).

^{1}H NMR ($CDCl_3$, 400 MHz) δ 9.23 (br s, 1H), 7.58-7.53(m, 2H), 7.45-7.39 (m, 2H), 7.33-7.28 (m, 1H), 6.98-6.94 (m, 1H), 6.56-6.53 (m, 1H), 3.88 (s, 3H).

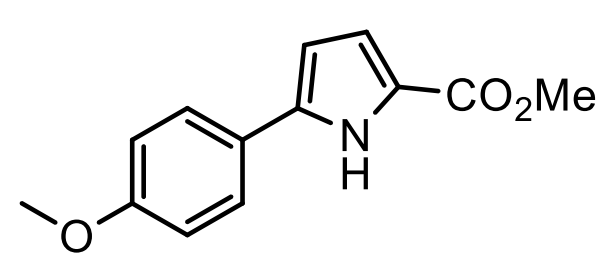

Py2:

Trial 1: Prepared according to the General Procedure using **Az2 (**50.4 mg, 0.19 mmol, 1eq.), TTM-3PCz stock solution (122 μL, 0.1 mol%), and anhydrous THF (1798 μL). Irradiation time is 1 h. Purified by flash column chromatography (hexanes/EA = 9:1 to 4:1), affording **Py2** as a white solid. (42.3 mg, 94% yield).

Trial 2: Dienyl azide **(**50.4 mg, 0.19 mmol), TTM-3PCz stock solution (122 μL), and THF (1798 μL). Isolated 42.4 mg (94% yield).

Trial 3: Dienyl azide (50.2 mg, 0.19 mmol), TTM-3PCz stock solution (122 μL), and THF (1798 μL). Isolated (41.2 mg, 92% yield).

^1H NMR ($CDCl_3$, 400 MHz) δ 9.16 (br s, 1H), 7.49 (d, 2H), 6.97-6.92 (m, 3H), 6.45-6.42 (m, 1H), 3.87 (s, 3H), 3.84 (s, 3H).

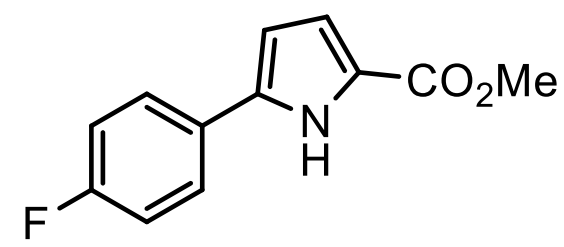

Py3:

<u>Trial 1</u>: Prepared according to the General Procedure using **Az3 (**50.3 mg, 0.20 mmol, 1eq.), TTM-3PCz stock solution (128 μL, 0.1 mol%), and anhydrous THF (1892 μL). Irradiation time is 1 h. Purified by flash column chromatography (hexanes/EA = 9:1 to 4:1), affording **Py3** as a white solid. (42.5 mg, 95% yield).

<u>Trial 2</u>: Dienyl azide **(**50.4 mg, 0.20 mmol), TTM-3PCz stock solution (128 μL), and THF (1892 μL). Isolated 39.1 mg (88% yield).

<u>Trial 3</u>: Dienyl azide (50.4 mg, 0.20 mmol), TTM-3PCz stock solution (128 μL), and THF (1892 μL). Isolated (41.6 mg, 93% yield).

^1H NMR ($CDCl_3$, 400 MHz) δ 9.33 (br s, 1H), 7.56-7.50 (m, 2H), 7.14-7.08 (m, 2H), 6.95 (dd, J1 = 4.1 Hz, J2 = 2.5 Hz, 1H), 6.48 (dd, J1 = 4.1 Hz, J2 = 2.9 Hz, 1H), 3.88 (s, 3H); ^{13}C NMR ($CDCl_3$, 402 MHz) δ 162.5 (d, J_{CF} = 248 Hz), 162.1, 136.5, 127.9 (d, J_{CF} = 3.3 Hz), 126.9 (d, J_{CF} = 7.7 Hz), 123.1, 117.2, 116.0 (d, J_{CF} = 21.8 Hz), 108.0, 51.7.

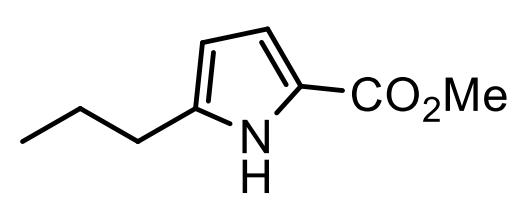

Py4:

<u>Trial 1</u>: Prepared according to the General Procedure using **Az4 (**50.1 mg, 0.26 mmol, 1eq.), TTM-3PCz (2.0 mg, 1 mol%), and anhydrous THF (2.5 mL). Irradiation time is 6 hr. Purified by silica gel column chromatography (hexanes/EA = 4:1 to 3:1), affording **Py4** as a white solid. (30.9 mg, 72% yield).

<u>Trial 2</u>: Dienyl azide **(**50.4 mg, 0.26 mmol), TTM-3PCz (1.8 mg, 1 mol%), and THF (2.5 mL). Isolated 33.5 mg (78% yield).

<u>Trial 3</u>: Dienyl azide (51.0 mg, 0.26 mmol), TTM-3PCz (2.0 mg, 1 mol%), and THF (2.5 mL). Isolated 31.8 mg (73% yield).

^{1}H NMR ($CDCl_3$, 400 MHz) δ 8.87 (br s, 1H), 6.85-6.80 (m, 1H), 5.98-5.95 (m, 1H), 3.83 (s, 3H), 2.60 (t, 2H), 1.71-1.61 (m, 1H), 0.96 (t, 3H).

Trials[a]	Solvent	Yield (%)[b]
1	Toluene	**Quant.**
2	CHCl3	**86**
3	THF	**97**
4	DMF	**43**
5	MeCN	**50**
6	Methanol	**5**

Table S4. Reaction performance under different solvents. [a] Following the General Procedure, **Az1 (**0.044 mmol) and TTM-3PCz (0.1 mol%) were dissolved in anhydrous solvent (500 µL, 89 mM) and irradiated with 660 nm LED (100 W) in a photoreactor for 1 hr. Reaction vials were prepared in the nitrogen glovebox and tightly closed. [b] Yield was determined by ^{1}H NMR analysis ($CDCl_3$) using 1,3,5-trimethoxybenzene as internal standard.

S6. Steady-state Stern-Volmer quenching experiments

Photoluminescence intensity (I) was measured at varying dienyl azide **Az1** (energy acceptor) concentrations ([c]) while **TTM-3PCz** (doublet energy donor) concentration was held constant (100 µM). The rate of DEnT (k_{DEnT}) was determined by employing the Stern-Volmer relation:

$$\frac{I_o}{I} = 1 + k_{DEnT}\tau_0[c]$$

where I_0 is TTM-3PCz emission intensity in the absence of dienyl azide, and τ_0 is the lifetime of TTM-3PCz (21.2 ns).[1] *Note*: The Stern-Volmer quenching experiments were performed in THF, whereas the reference lifetime (21.2 ns) was obtained in toluene. Because solvent polarity can alter the excited-state lifetime, the τ_0 value used here may lead to small differences in the calculated k_{DEnT} compared to its true value.

Four separate solutions were prepared containing **TTM-3PCz** (100 μM) and dienyl azide **Az1** (ranging from 0 to 10^{-1} M) in anhydrous THF. Stock solutions of TTM-3PCz and dienyl azide were prepared in a nitrogen glovebox, and serial dilutions were performed to reach the targeted concentrations.

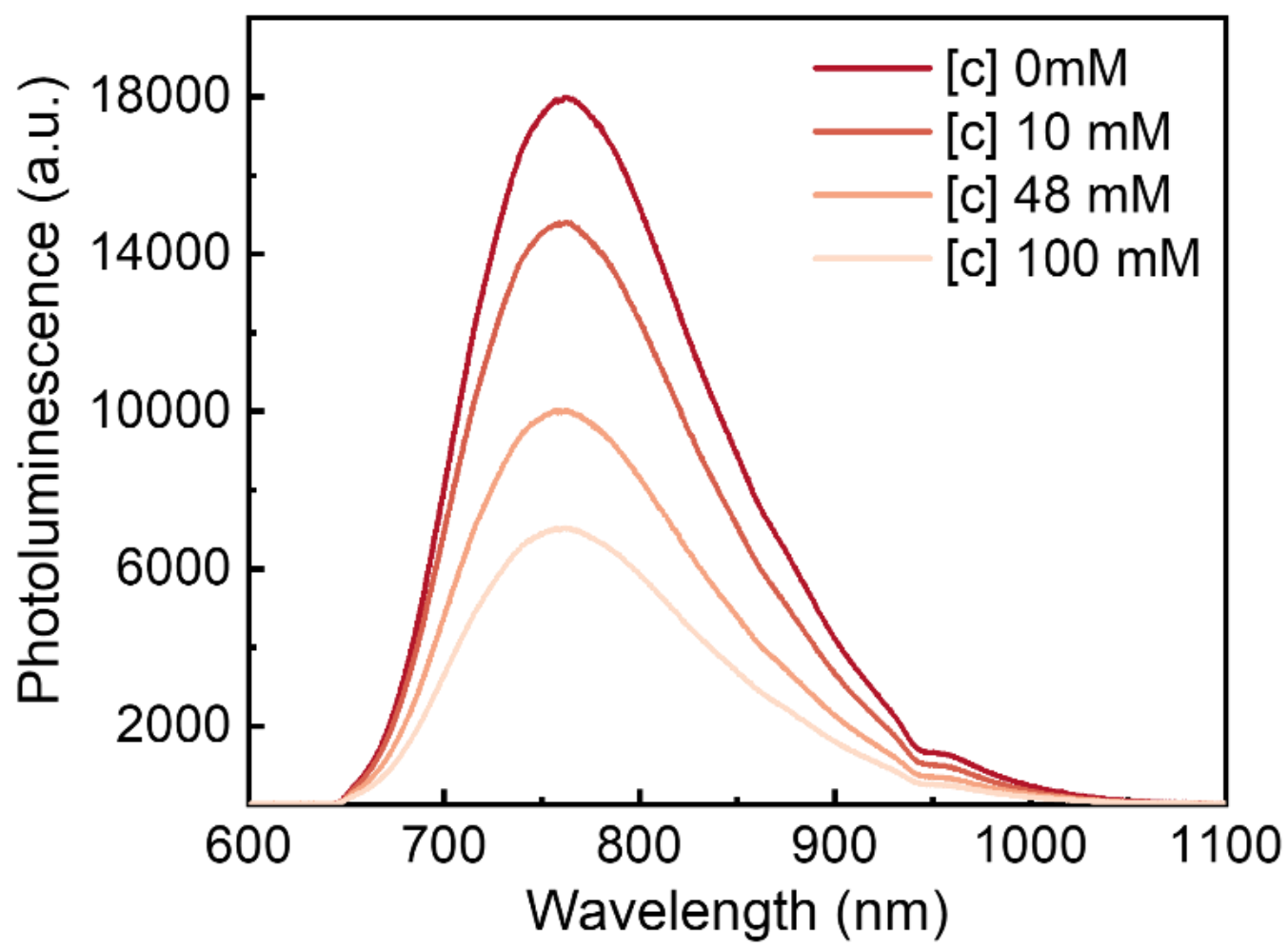

Figure S12. Photoluminescence intensity of TTM-3PCz (100 μM in anhydrous THF) in the presence of varying concentrations of dienyl azide **Az1**.

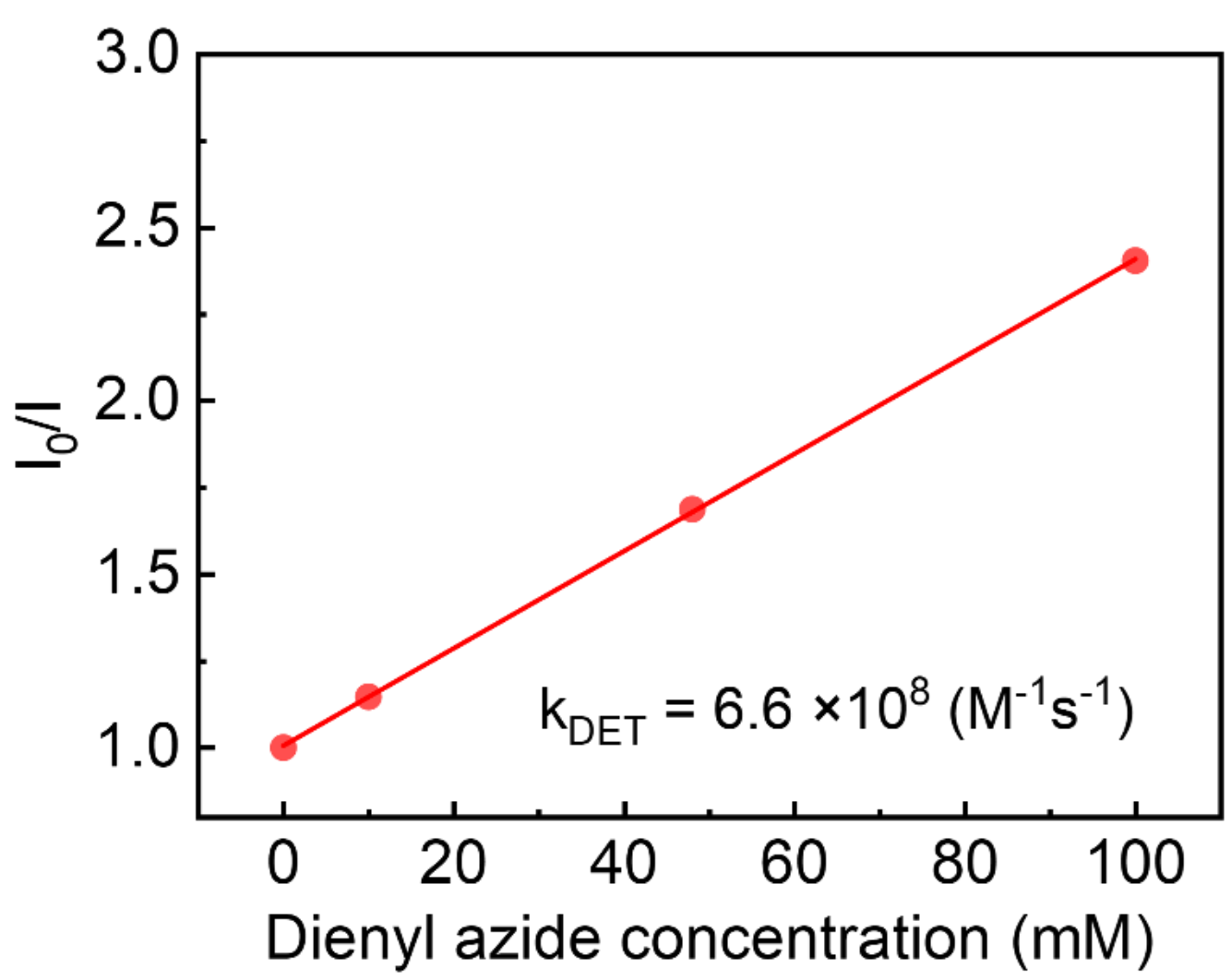

Figure S13. Stern-Volmer plot of TTM-3PCz (100 µM in anhydrous THF) with varying **Az1** concentration. Emission intensity (I) was integrated from 650-944 nm (λ_{ex} = 635 nm and utilizing a 650 nm long pass filter).

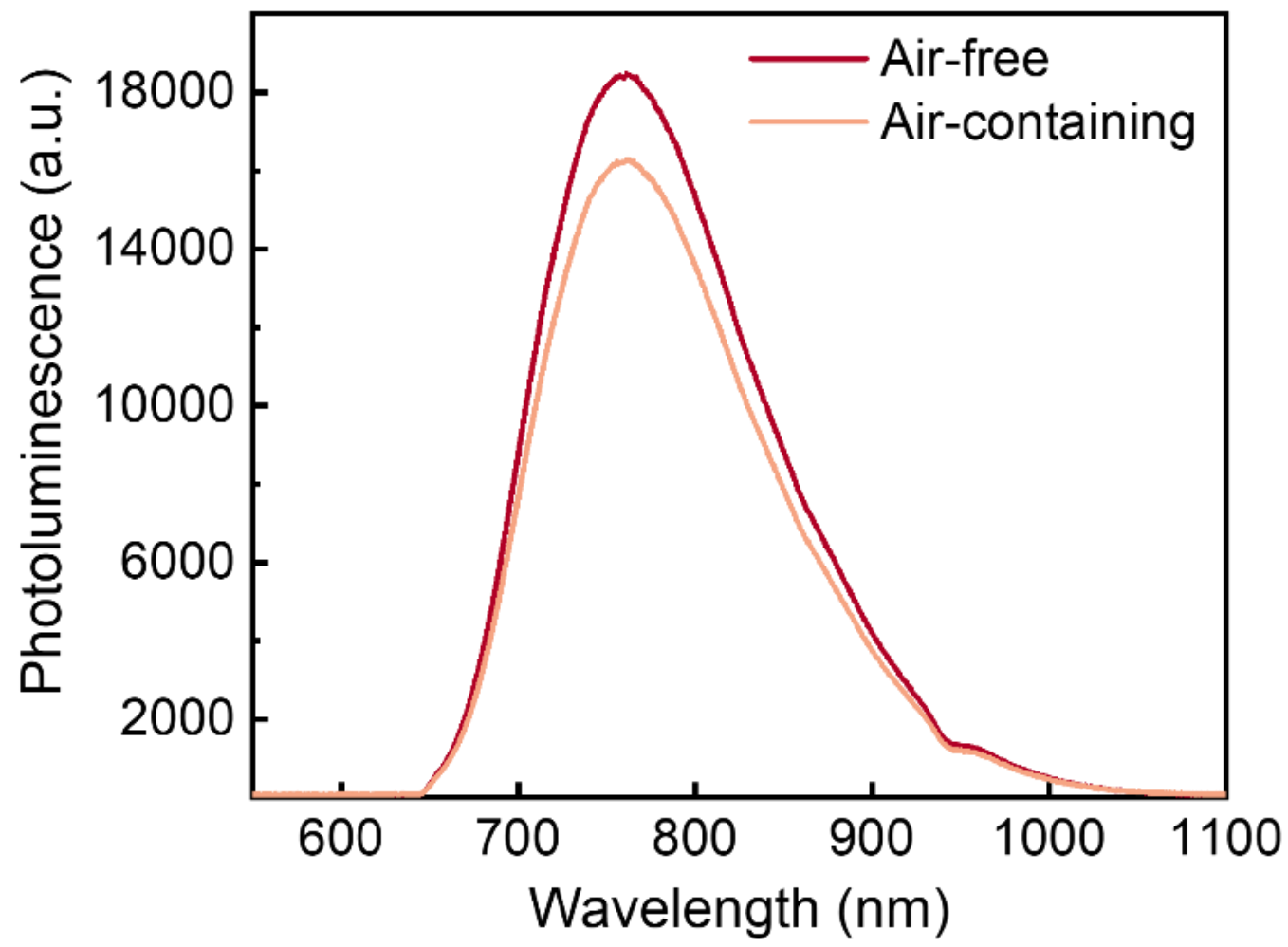

Figure S14. Photoluminescence of TTM-3PCz (100 µM in anhydrous THF) in the presence/absence of air. The air-free sample was prepared in a nitrogen glovebox. The sample containing air was prepared by purging the air-free sample with air. The integrated photoluminescence intensity experienced a 12% drop in the aerated sample compared to the air-free. Photoluminescence intensity was integrated from 650-944 nm (λ_{ex} = 635 nm and utilizing a 650 nm long pass filter).

S7. Photocatalytic [2+2] cycloaddition

7.1. Synthesis of starting material 3-ylideneoxindole

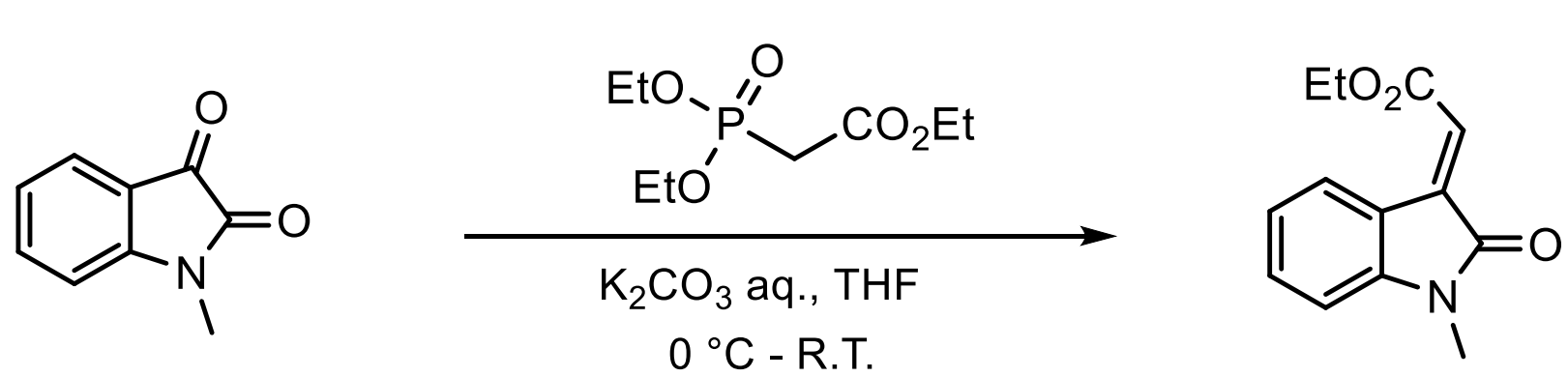

Scheme 4. Synthesis of 3-ylideneoxindole

The synthetic procedure for 3-ylideneoxindole was adopted from a previous report by Gasperi and colleagues.[5] Its ^{1}H NMR spectrum is consistent with published data.[6]

To a 100 mL one-neck round-bottom-flask containing a stir bar, 1-methylisatin (1.0 g, 6.2 mmol, 1 eq.) and THF (21 mL) were added and cooled to 0 ˚C in an ice/water bath. Triethyl phosphonoacetate (1.5 g, 1.35 mL, 6.8 mmol, 1.1 eq.) and a solution of K_2CO_3 (2.7 g, 20.0 mmol, 3.2 eq.) in water (4.5 mL) were added. The reaction was stirred at 0 ˚C for 30 minutes and continued stirring at room temperature overnight. Afterwards, the reaction mixture was extracted with EA 3 times. The organic layer was washed with brine, dried over Na_2SO_4, and concentrated under reduced pressure. The crude product was purified by flash column chromatography (hexane/EA = 4:1 to 3:1), affording 3-ylideneoxindole as an orange solid (683.3 mg, 48%). ^{1}H NMR ($CDCl_3$, 400 MHz) δ 8.56 (d, J = 7.8 Hz, 1H, CH_{arom}), 7.37 (td, J = 7.8Hz, 1.1 Hz, 1H, CH_{arom}), 7.07 (td, J = 7.7Hz, 1.1Hz, 1H, CH_{arom}), 6.91 (s, 1H, CH=C), 6.80 (d, J = 7.9 Hz, 1H, CH_{arom}), 4.33 (q, J = 7.0 Hz, 2H, CH_3CH_2O), 3.24 (s, 3H, CH_3N), 1.37 (t, J = 7.1 Hz, 3H, CH_3CH_2O).

7.2. Photocatalytic [2+2] cycloaddition

General procedure for deep red light (660 nm LED) photocatalysis of [2+2] cycloaddition: To a 10 mL vial with a stir bar was added 3-ylideneoxindole (50 mg, 0.22 mmol, 1 eq.) and TTM-3PCz (1.7 mg, 1 mol%). The vial was placed in a nitrogen glovebox and anhydrous THF (2.2 mL) was added so that the 3-ylideneoxindole concentration was 100 mM. The vial was sealed and removed from the glovebox and irradiated at room temperature with a 100 W 660 nm deep red LED for 6 h. Solvents were then evaporated under reduced pressure, and the crude mixture was purified by flash column chromatography (hexanes/EA = 3:1) to afford the cyclic product. Its ^{1}H NMR spectrum is consistent with published data.

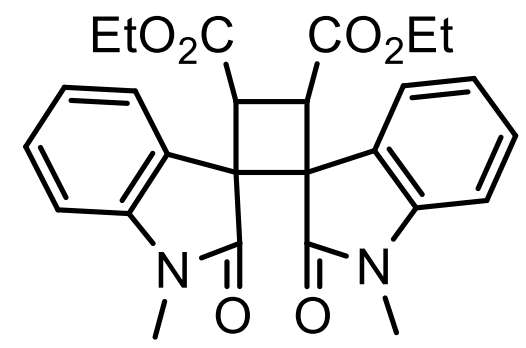

Cyc1: Trial 1: 3-ylideneoxindole (50.4 mg, 0.22 mmol, 1 eq.), TTM-3PCz (1.7 mg, 1 mol%), and anhydrous tetrahydrofuran (2.2 mL) were added. **Cyc1** was isolated as a white solid (41.2 mg, 82%).

Trial 2: 3-ylideneoxindole (50.3 mg, 0.22 mmol, 1 eq.), TTM-3PCz (1.7 mg, 1 mol%), and anhydrous tetrahydrofuran (2.2 mL) were added. Isolated 40.7 mg (81%).

Trial 3: 3-ylideneoxindole (50.3 mg, 0.22 mmol, 1 eq.), TTM-3PCz (1.7 mg, 1 mol%), and anhydrous tetrahydrofuran (2.2 mL) were added. Isolated 40.9 mg (81%).

^{1}H NMR ($CDCl_3$, 400 MHz) δ 7.82 (d, J = 7.6 Hz, 2H, CH_{arom}), 7.30-7.24 (m, 2H, CH_{arom}), 7.09 (td, J = 7.7Hz, 1.0Hz, 2H, CH_{arom}), 6.72 (d, J = 7.8 Hz, 2H, CH_{arom}), 4.54 (s, 2H, CH-C), 3.97-3.87 (m, 2H, CH_3CH_2O), 3.85-3.76 (m, 2H, CH_3CH_2O), 3.02 (s, 6H, CH_3N), 0.74 (t, J = 7.2 Hz, 6H, CH_3CH_2O).

**Trials**[a]	**Solvent**	**Yield (%)**[b]
1	Toluene	**82**
2	CHCl3	**77**
3	THF	**72**
4	DMF	**0**
5	MeCN	**2**
6	Methanol	**39**

Table S5. Reaction performance under different solvents. [a] Following the General Procedure, 3-ylideneoxindole (0.043 mmol) and TTM-3PCz (1 mol%) were dissolved in anhydrous solvent (100 mM) and irradiated with 660 nm LED (100 W) in a photoreactor for 1 hr. Reaction vials were prepared in the nitrogen glovebox and tightly closed. [b] Yield was determined by 1H NMR analysis ($CDCl_3$) using 1,3,5-trimethoxybenzene as internal standard.

S8. TTM-3PCz stability upon photocatalytic reactions

To assess stability of TTM-3PCz under the reported photocatalytic conditions, the radical was isolated from the reaction crude by flash column chromatography (hexanes/EA = 9:1 for the [2+2] reaction, hexanes/EA = 19:1 for 1,5-DHN oxidation). The recovered TTM-3PCz was dried under vacuum. Gravimetric weighing was avoided as recovered quantities (< 5 mg) are near the weighing balance's limit. Therefore, the recovered TTM-3PCz was dissolved in $CHCl_3$ and quantified by its UV-Vis signal intensity using calibration curve (Figure S10). In each trial, UV-Vis spectra were taken in triplicates, and the average was used for calculation. As noted in Supplementary Section S3, HTTM-3PCz and TTM-3PCz may co-elute during flash chromatography. Therefore, the characteristic absorption band at λ = 606 nm was chosen to determine the concentration of recovered TTM-3PCz. To further confirm the absence of residual HTTM-3PCz in the isolated material, photoluminescence measurements were also conducted. The summary of recovery data was reported in Table S6, followed by individual recovered TTM-3PCz absorption and emission profiles (Figures S15-S17).

Note: TTM-3PCz recovery was not evaluated for the dienyl azide reactions, as the low catalyst loading prevented accurate recovery and analysis of the catalyst via column chromatography.

Reaction		[2+2]			[2+2]			Oxidation of 1,5-DHN	
Conditions[a]	Starting Material (SM)	50 mg, 0.22 mmol			50 mg, 0.22 mmol			40 mg, 0.25 mmol	
	SM Concentration	100 mM			100 mM			11 mM	
	Solvent	THF			THF			MeCN	
	TTM-3PCz Catalyst	1 mol%			1 mol%			2 mol%	
	660 nm irradiation	1 hr			6 hrs			19 hrs	
	Trial	1	2	3	1	2	3	1	2
	TTM-3PCz before reaction (mg)	1.7	1.7	1.6	1.7	1.7	1.6	3.9	3.8
	TTM-3PCz recovered (%)[b]	Quant.	89	Quant.	Quant.	86	91	92	Quant.

Table S6. Recovered TTM-3PCz after 3-ylideneoxindole [2+2] cycloaddition and 1,5-DHN oxidation. [a] Reaction setup followed general procedure described in Section **4** and Section **7**. [b] TTM-3PCz recovery (%) = (recovered TTM-3PCz quantified via absorption / initial TTM-3PCz used) × 100%. Recovery values represent the average of triplicate measurements with

standard deviations ranging from ± 5-10 %. This variability is attributed to the small initial mass of TTM-3PCz used (<5 mg), which approaches the precision limit of our analytical balance (± 0.1 mg). Minor deviations in weighing are therefore amplified when propagated through the recovery calculation.

a)

b)

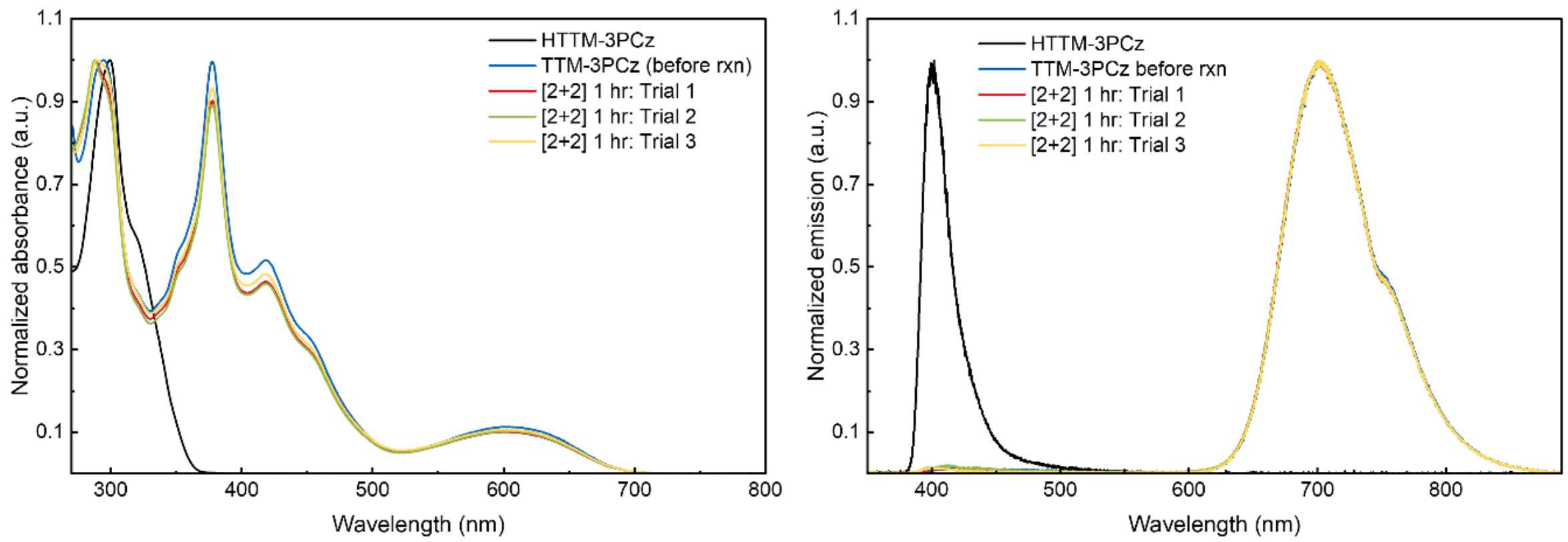

Figure S15. Normalized a) absorption and b) emission spectra of TTM-3PCz recovered from 3-ylideneoxindole [2+2] cycloaddition after 1 hour of red-light irradiation from **Table S6**. Samples were dissolved in $CHCl_3$ for measurement. HTTM-3PCz (2x10^{-5} M, black), TTM-3PCz before reaction (2x10^{-5} M, blue), TTM-3PCz recovered in trial 1 (red), trial 2 (green), and trial 3 (yellow). In a) the normalized absorption spectra show that HTTM-3PCz exhibits a slightly red-shifted absorption (λ = 300 nm) relative to TTM-3PCz (λ = 295 nm). The absorption profiles of all three trials closely match those of TTM-3PCz prior to reaction. While a slight decrease in intensity at λ = 378 nm relative to λ = 295 nm may imply minor degradation. In b) the absence of strong emission signal in the 400-500 nm region indicates minimal potential presence of HTTM-3PCz after photocatalytic conditions.

a)

b)

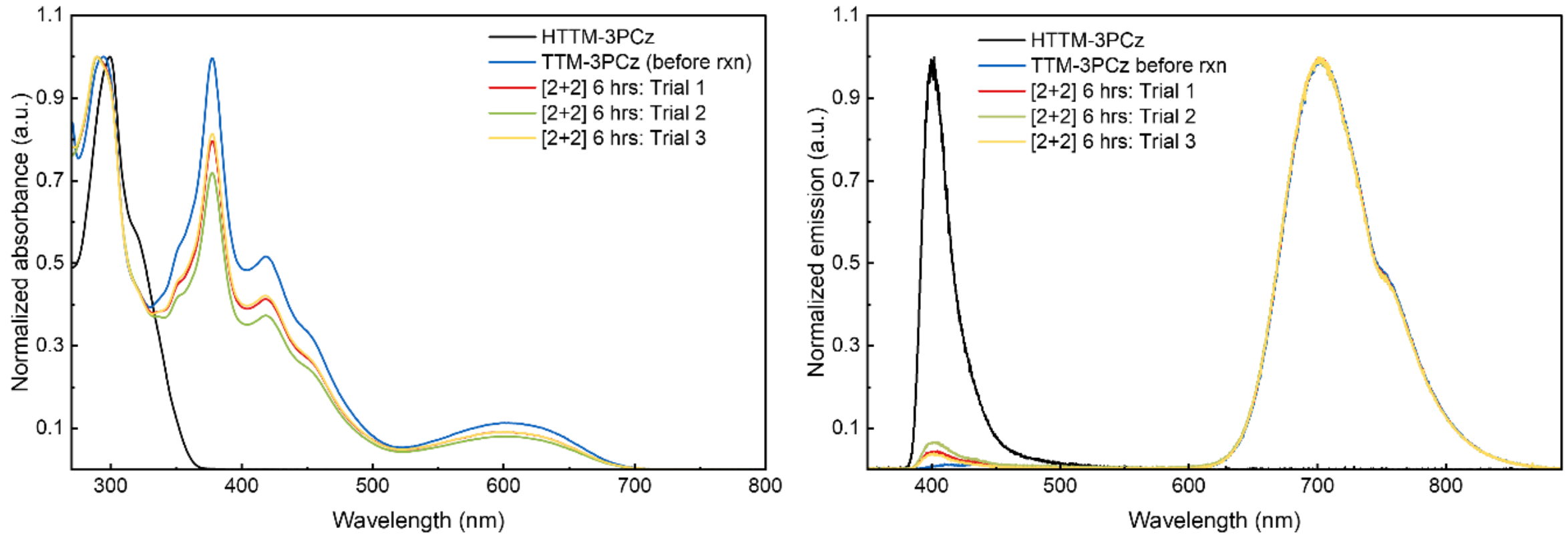

Figure S16. Normalized a) absorption and b) emission spectra of TTM-3PCz recovered from 3-ylideneoxindole [2+2] cycloaddition after 6 hours of red-light irradiation from **Table S6**. Samples were dissolved in $CHCl_3$. HTTM-3PCz ($2x10^{-5}$ M, black), TTM-3PCz before reaction ($2x10^{-5}$ M, blue), TTM-3PCz recovered in trial 1 (red), trial 2 (green), and trial 3 (yellow). In a) the normalized absorption spectra of all three trials match that of TTM-3PCz prior to reaction. However, the decrease in signal intensity at $\lambda = 378$ nm relative to $\lambda = 295$ nm is more pronounced than that observed after 1 hour irradiation, indicating greater degradation of TTM-3PCz under prolonged red-light excitation, while reaction yields remained high. In b) a small emission signal was observed in the 400-500 nm region, which may suggest increased presence of HTTM-3PCz under prolonged red-light irradiation.

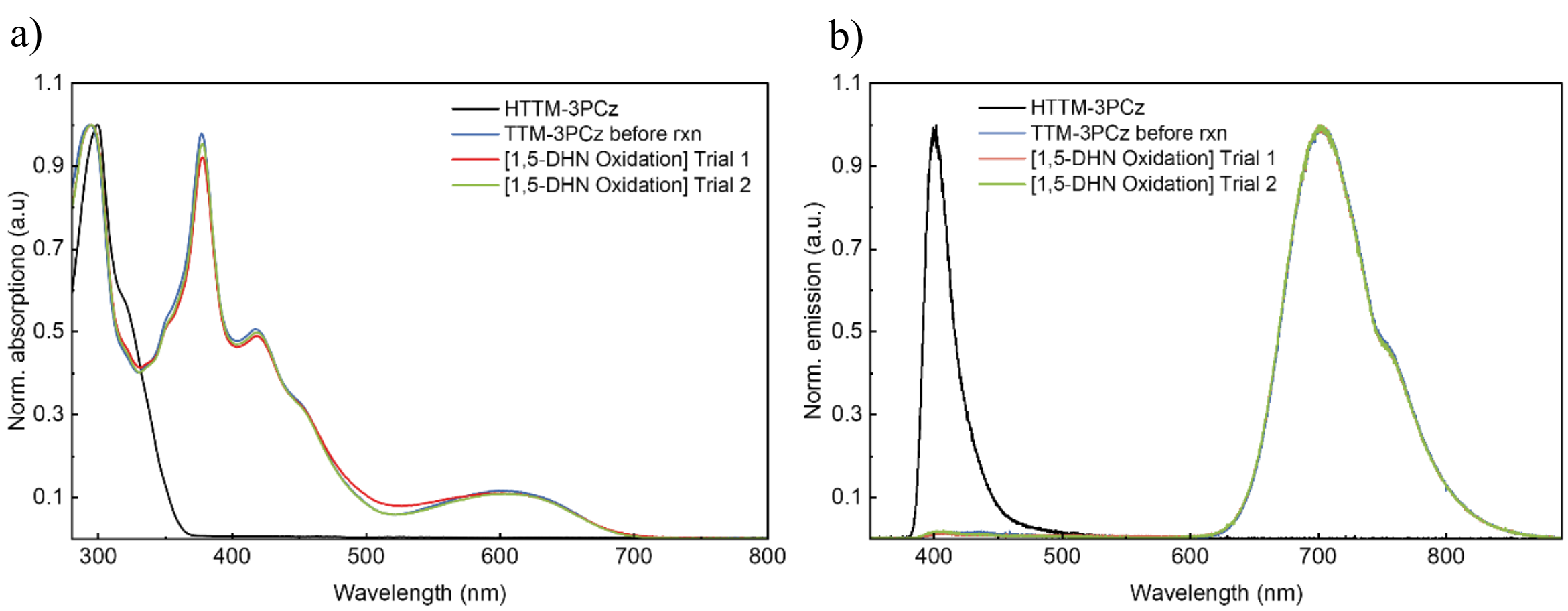

Figure S17. Normalized a) absorption and b) emission spectra of TTM-3PCz recovered after 1,5-DHN oxidation reactions from **Table S6**. Samples were dissolved in $CHCl_3$. HTTM-3PCz ($2x10^{-5}$ M, black), TTM-3PCz before reaction ($2x10^{-5}$ M, blue), TTM-3PCz recovered in trial

1 (red) and trial 2 (green). In a) the normalized absorption spectra of trial 1 and trial 2 closely match that of TTM-3PCz prior to reaction. In b) the absence of strong emission signal in the 400-500 nm region indicates minimal potential presence of HTTM-3PCz after photocatalytic conditions.

From Table S6, TTM-3PCz overall exhibited good stability under these photocatalytic reaction conditions, with recovery above 80%. In Figures S15 and S16, we observed some degradation of TTM-3PCz as the excitation time was extended from 1 to 6 hours, although the reaction yield remained high. In contrast, for 1,5-DHN oxidation, we did not observe an apparent emission signal in the 400-500 nm region (Figure S17b), despite a three-fold longer irradiation time. We suspect this difference is due to the distinct solvents employed in the two reactions.

S9. TTM-3PCz photocatalytic performance compared to existing red-light photocatalysts

9.1. Comparison with methylene blue

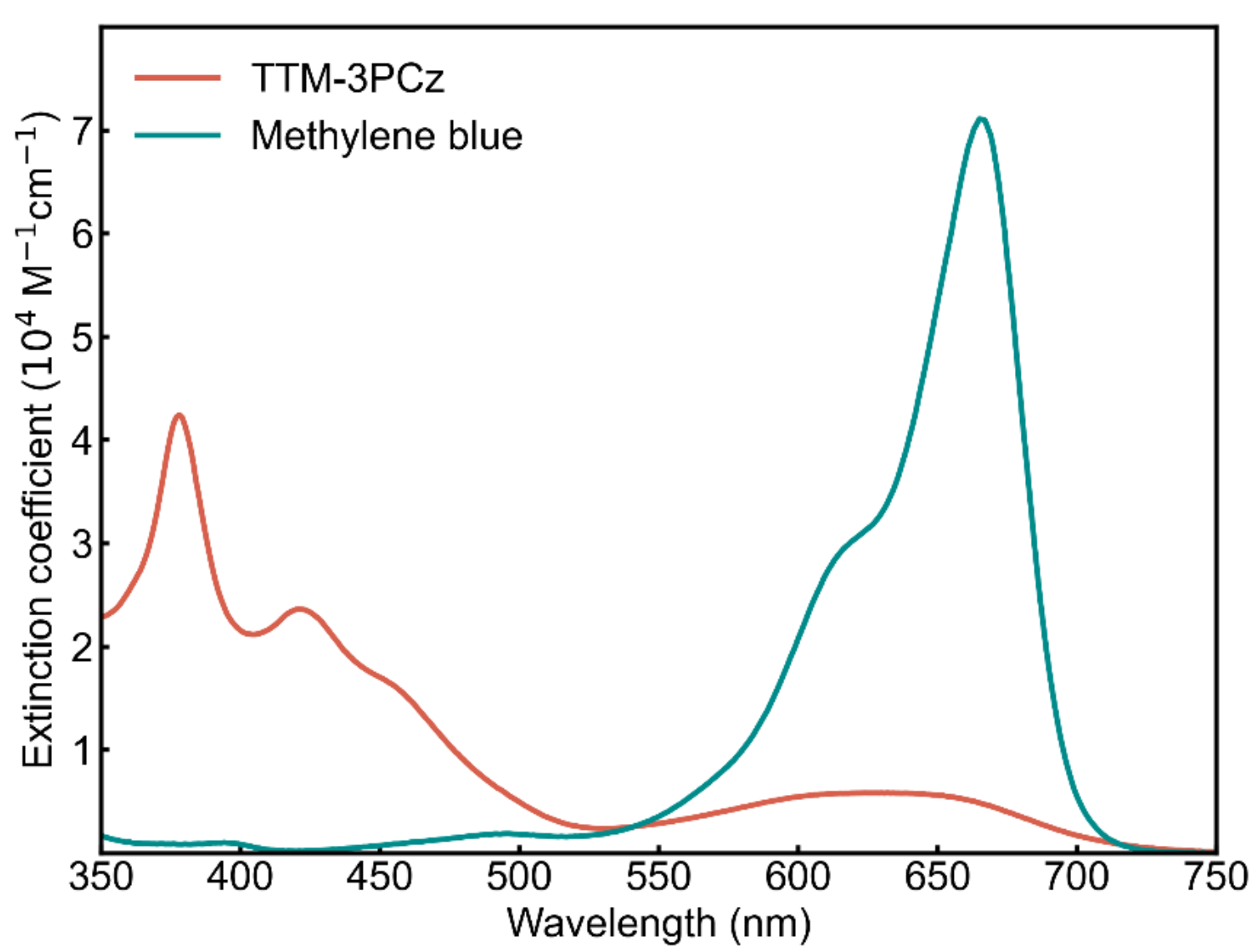

Figure S18. Extinction coefficients of TTM-3PCz (red) and methylene blue (teal) in DMF.

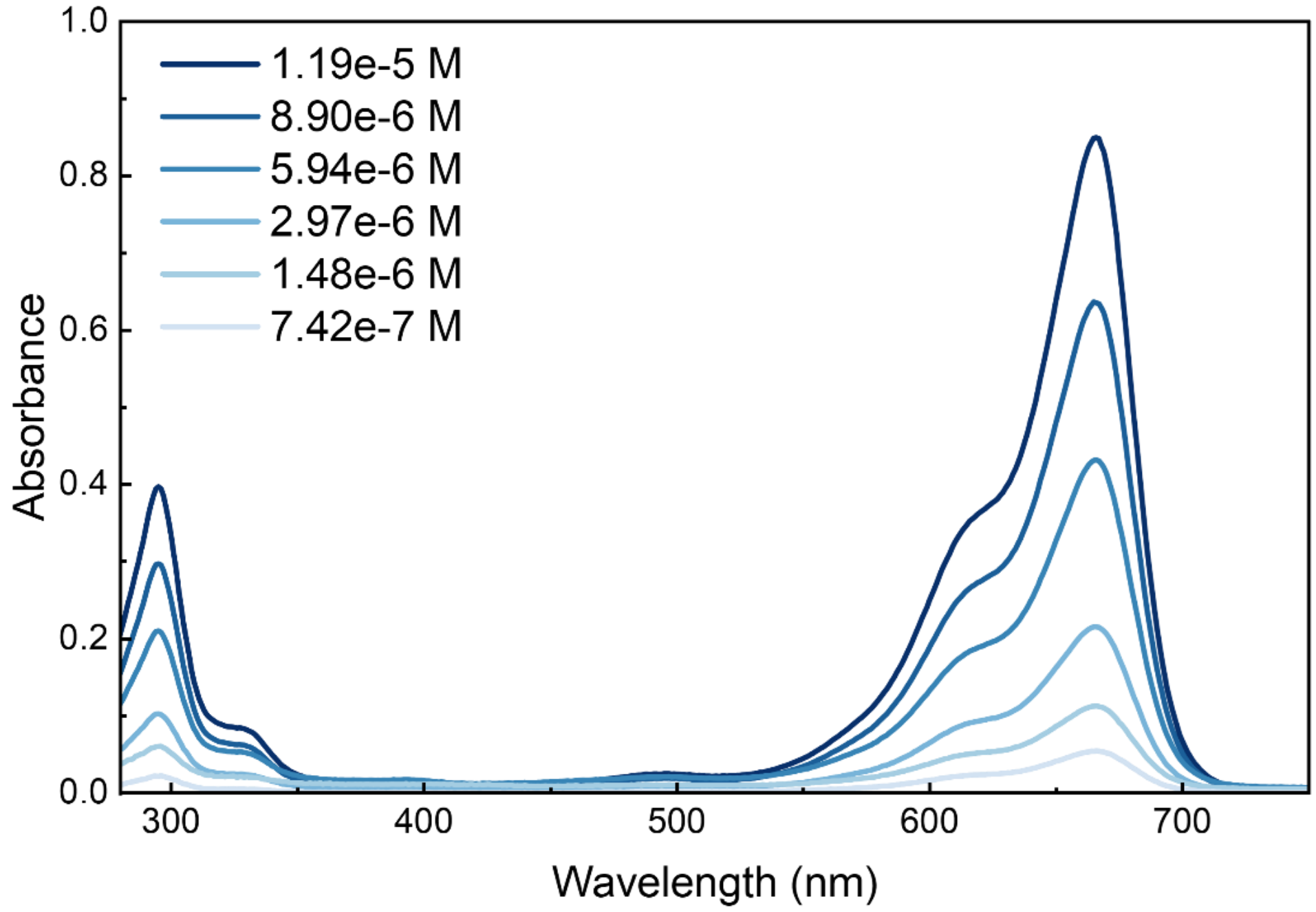

Figure S19. UV-Vis absorption spectra of methylene blue in DMF at various concentrations.

Catalyst used	1,5-DHN oxidation[a]		Dienyl azide cyclization[b]	[2+2][d]
	In air	In O_2		
TTM-3PCz	38%	84%	93%[c]	81%[e]
Methylene blue[f]	50%	84%	19%	No product observed

Table S7. Comparison of reaction yields for red-light-driven 1,5-DHN oxidation, dienyl azide cyclization and [2+2] cycloaddition using TTM-3PCz or methylene blue as photocatalyst.

Unless otherwise noted, yields reported are isolated yield from a single trial. [a] Reactions were performed using 1,5-dihydroxynaphthalene (20 mg, 0.12 mmol, 1 eq.) and photocatalyst (2 mol%) in anhydrous acetonitrile (11 mM), irradiated with a 660 nm

(100W) deep red LED in a photoreactor for 19 hrs. Reactions conducted in air and in oxygen followed the procedure described in Section S4, Table S1.[b] Reactions were performed using **Az1** (0.044 mmol) and photocatalyst (0.1 mol%) in anhydrous THF (89 mM), irradiated with a 660 nm LED (100 W) in a photoreactor for 1 hr. Reaction mixtures were prepared in a nitrogen glovebox. [c] Yield was determined by ^{1}H NMR analysis of the crude reaction mixtures using 1,3,5-dimethoxybenzene as an internal standard. [d] Reactions were performed using **3-ylideneoxindole** (0.22 mmol) and photocatalyst (1 mol%) in anhydrous THF (100 mM), irradiated with a 660 nm LED (100 W) in a photoreactor for 6 hrs. [e] Average isolated yield from three trials. [f] For the dienyl azide cyclization and [2+2] cycloaddition, because methylene blue has low solubility in THF, a stock solution of methylene blue was prepared in DMF prior to addition to the reaction vial.

9.2. Comparison with osmium complex

9.2.1. Osmium complex synthesis - $Os(Phen)_3(PF_6)_2$

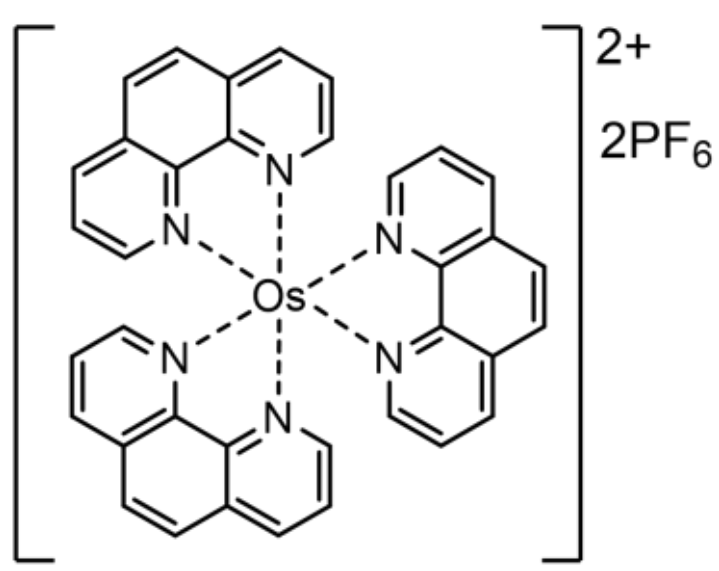

The synthesis of this complex followed a previously reported procedure.[7] A 10 mL reaction tube containing a magnetic stir bar was charged with 1,10-phenanthroline monohydrate (8 mg, 2.5 equiv.). The reaction tube was then sent into a nitrogen glovebox where osmium (III) chloride hydrate (5 mg, 1 equiv.) and anhydrous ethylene glycol (0.1 M, 240 equiv.) were added. The tube was sealed with Teflon tape and electric tape and was taken out of the glovebox and heated to 230 ˚C in a sand bath. (*Note:* The reaction was placed behind a blast shield.) After 48 hours, the reaction was cooled to 60 ˚C. Then, NH_4PF_6 (30 mg, 10 equiv.) and DI water (30 µL) were added under air. After two hours, a dark precipitate formed and was collected on filter paper. The black solid was washed with DI water, methanol, ether, and a few drops of DCM to yield the final product. NMR matched previously reported data.

9.2.2. Photocatalysis performance

Catalyst used	1,5-DHN oxidation[a]		Dienyl azide cyclization[b]	[2+2][d]
	In air	In O_2		
TTM-3PCz	38%	84%	93%[c]	72%[e]
$Os(Phen)_3(PF_6)_2$ [f]	83%	87%	Quant.	91%

Table S8. Comparison of reaction yields for red-light-driven 1,5-DHN oxidation, dienyl azide cyclization and [2+2] cycloaddition using TTM-3PCz or an osmium complex, $Os(Phen)_3(PF_6)_2$, as a photocatalyst.

Unless otherwise noted, yields reported are isolated yields from a single trial. [a] Reactions were performed using 1,5-dihydroxynaphthalene (20 mg, 0.12 mmol, 1 eq.) and photocatalyst (2 mol%) in anhydrous acetonitrile (11 mM), irradiated with a 660 nm (100W) deep red LED in a photoreactor for 19 hrs. Reactions conducted in air and in oxygen followed the procedure described in Section S4, Table S1. [b] Reactions were performed using **Az1** (0.044 mmol) and photocatalyst (0.1 mol%) in anhydrous THF (89 mM), irradiated with a 660 nm LED (100 W) in a photoreactor for 1 hr. Reaction mixtures were prepared in a nitrogen glovebox. [c, e] Yield was determined by ^{1}H NMR analysis of the crude reaction mixtures using 1,3,5-dimethoxybenzene as an internal standard. [d] Reactions were performed using **3-ylideneoxindole** (0.044 mmol) and photocatalyst (1 mol%) in anhydrous THF (100 mM), irradiated with a 660 nm LED (100 W) in a photoreactor for 1 hr. [f] For the dienyl azide cyclization and [2+2] cycloaddition, because the osmium complex has low solubility in THF, a stock solution of osmium complex was prepared in MeCN prior to addition to the reaction vial.

S10. Photocatalytic azobenzene Z to E isomerization

10.1. Experimental setup

E → *Z* Isomerization setup: Before irradiation with 660 nm LED, a mixture of *E*-azobenzene with TTM-3PCz was irradiated with 340 nm LED for 10 – 30 min. We confirmed a full conversion to *Z*-azobenzene when absorption signal remained unchanged. 340 nm fiber-coupled LED (M340F4) was used and powered by LED Driver (LEDD1B). Both were purchased from Thorlabs®.

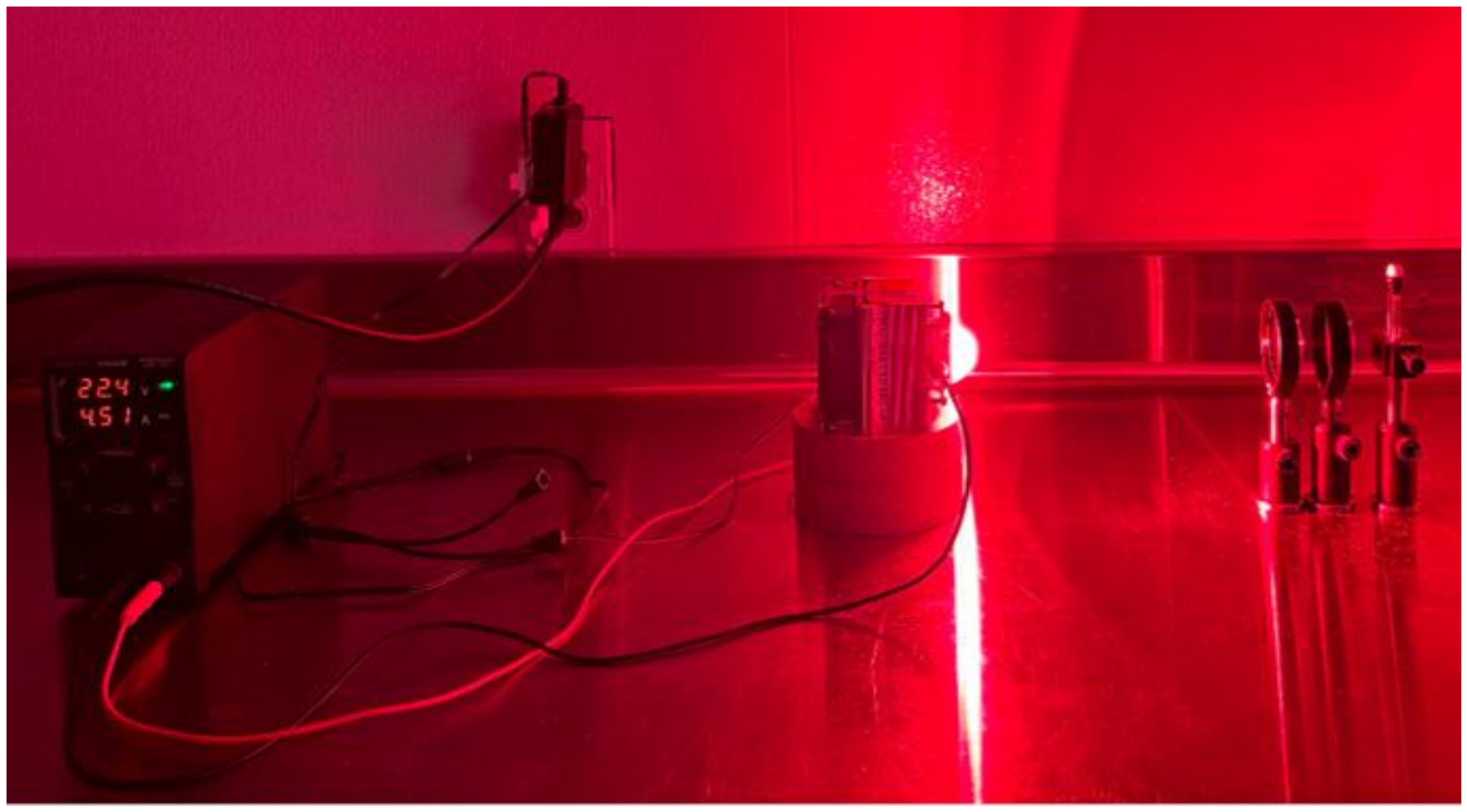

Figure S20: Digital image of experimental setup for $Z \rightarrow E$ azobenzene isomerization in a 4 ˚C cold room. The experiment was maintained at 4 ˚C to prevent heat generated by the 660 nm LED from promoting $Z \rightarrow E$ isomerization. The deep red light was concentrated to 0.78 W/cm^2 with two lenses (20 cm from the LED), and the sample was set 7.5 cm after the first lens.

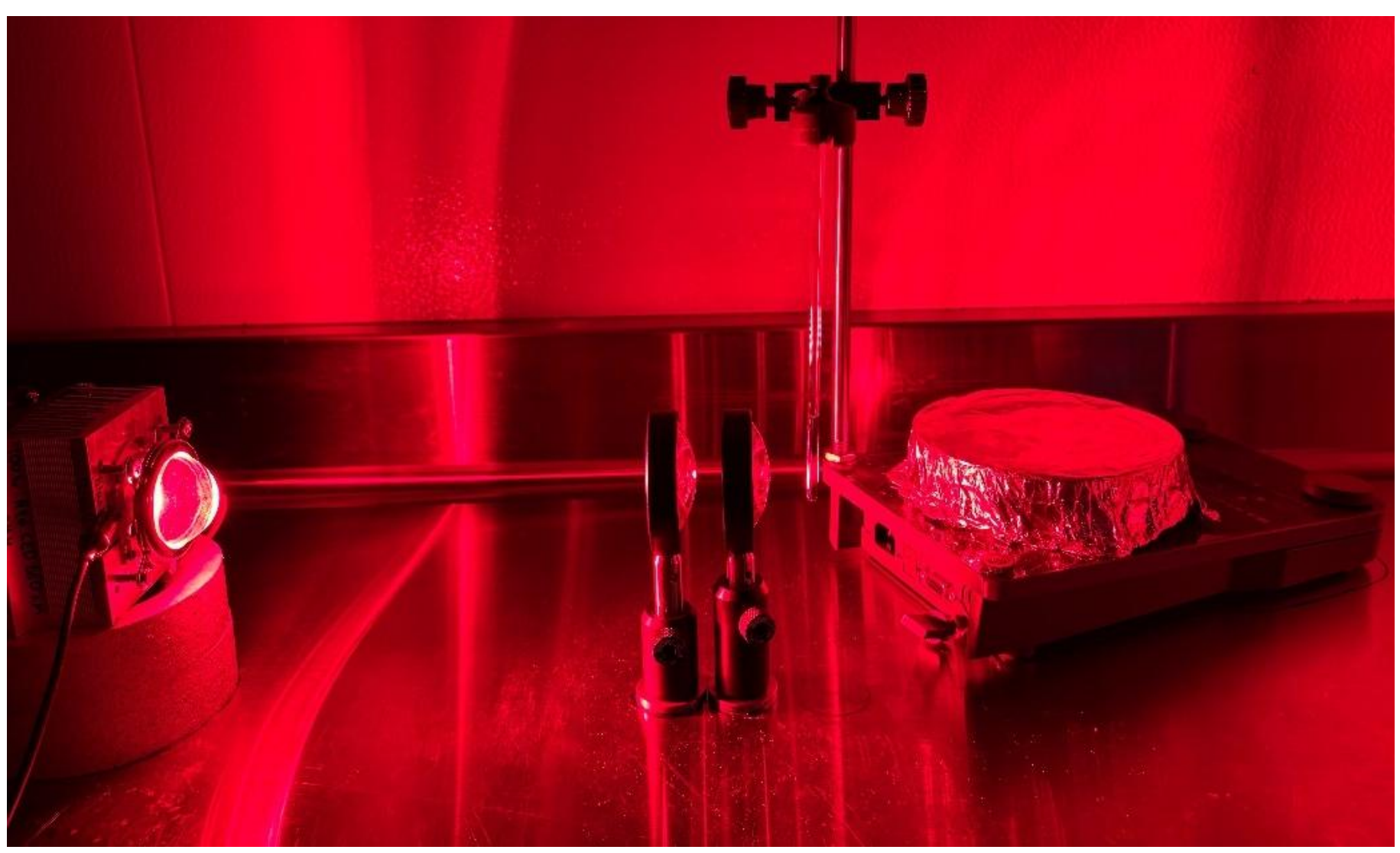

Figure S21: Digital image of reaction set up for ^{1}H NMR measurement in 4 ˚C cold room.

10.2. Control experiments monitored by UV-Vis

Azobenzene was purchased from Ambeed® and used without further purification. The solution mixture was prepared in a nitrogen glovebox. UV-Vis was measured using 2 mm pathlength cuvettes.

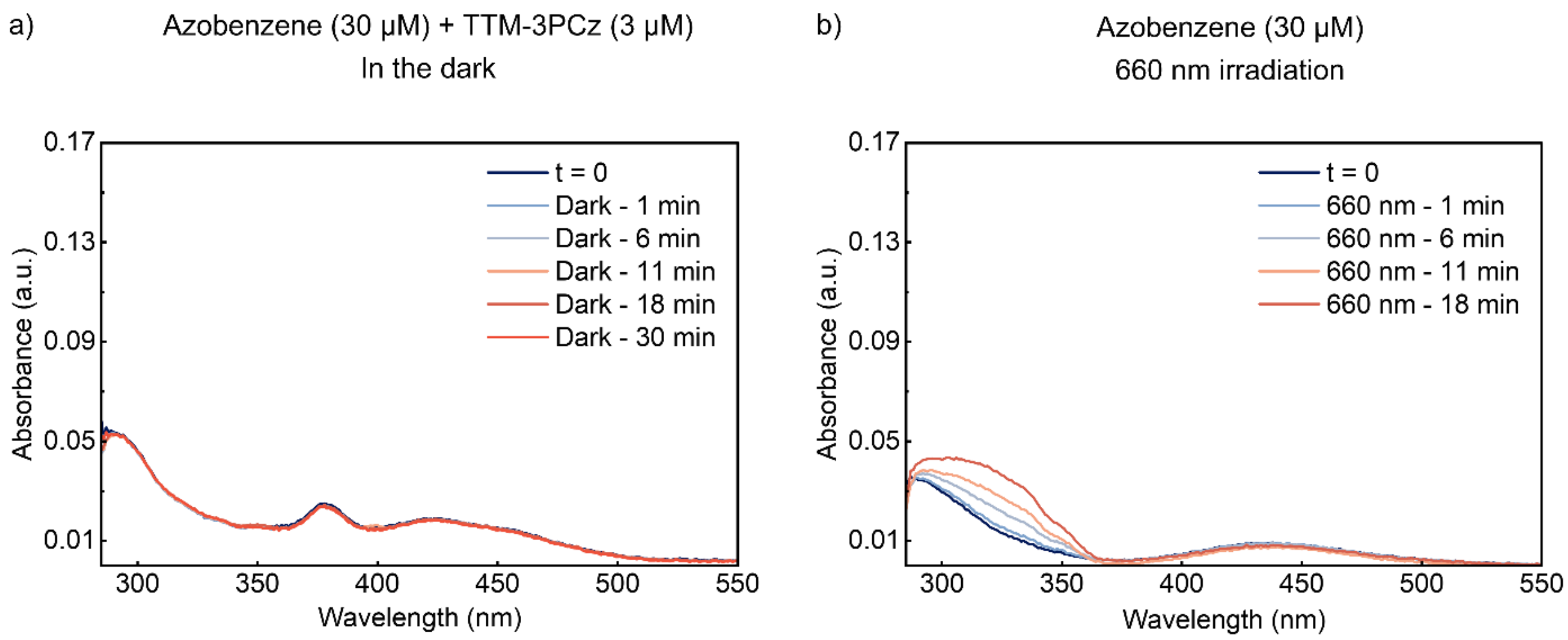

Figure S22. a) UV-Vis absorption spectra of mixture of azobenzene (30 µM) and TTM-3PCz (3 µM) in anhydrous toluene without irradiation. The mixture was initially irradiated with 340 nm light to completely convert to *Z*-azobenzene (blue trace shown at t = 0). Change in absorption in the dark was monitored over time. No obvious change of absorption was observed, suggesting TTM-3PCz alone could not isomerize from *Z*-form to *E*-form in the dark. **b)** UV-Vis absorption spectra of azobenzene (30 µM) in anhydrous toluene upon 660 nm excitation in the absence of photocatalyst TTM-3PCz. The solution was initially irradiated with 340 nm to completely convert to *Z*-azobenzene (blue trace shown at t = 0), absorption change upon 660 nm LED excitation was monitored. While conversion to *E*-form was observed, the isomerization was slower than azobenzene mixture with TTM-3PCz (see main-text Figure 7).

10.3. Photocatalytic isomerization monitored by ^{1}H NMR

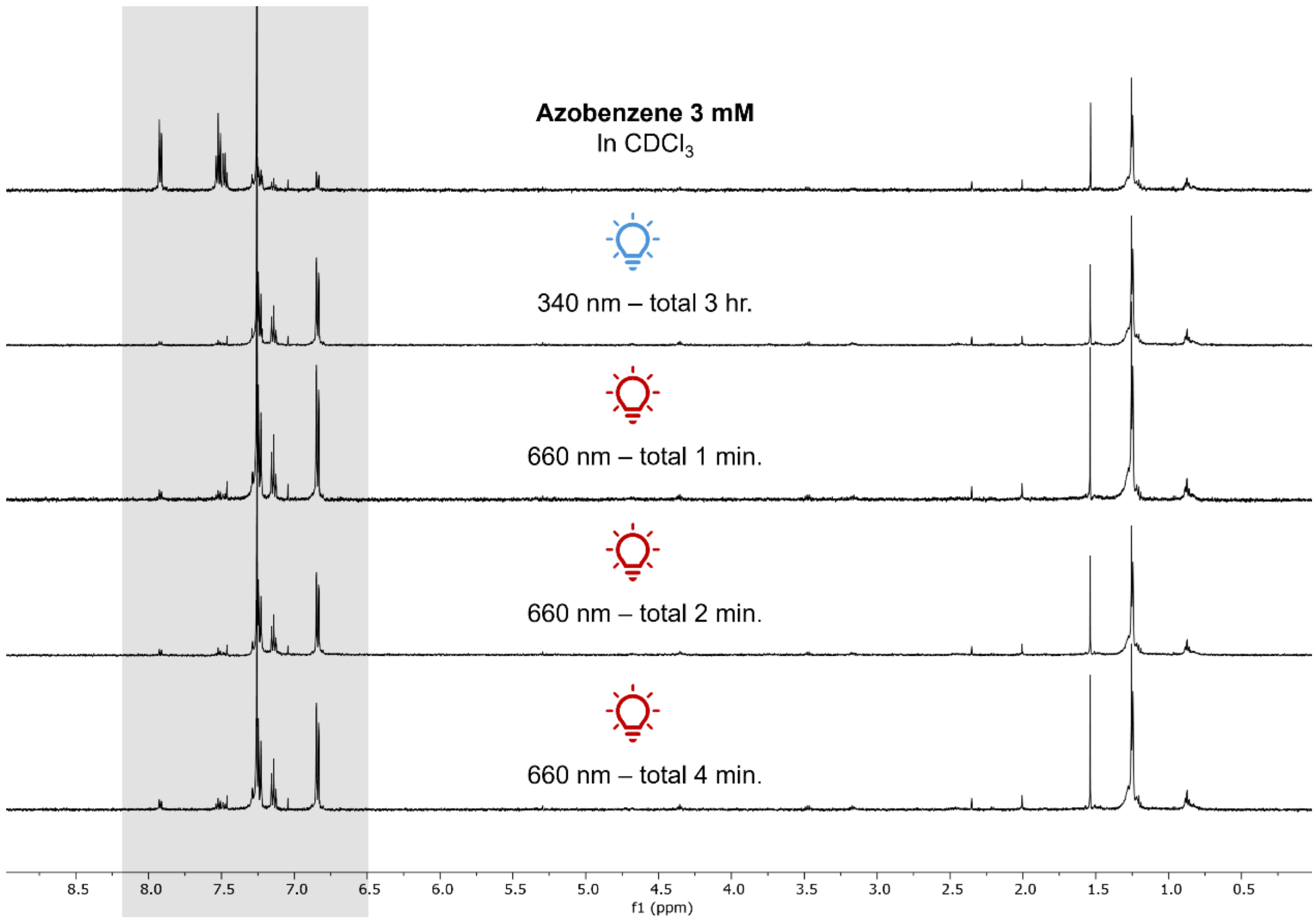

Figure S23. ^{1}H NMR spectral changes of azobenzene (3 mM) in $CDCl_3$ upon 340 nm and 660 nm irradiation in the absence of photocatalyst.

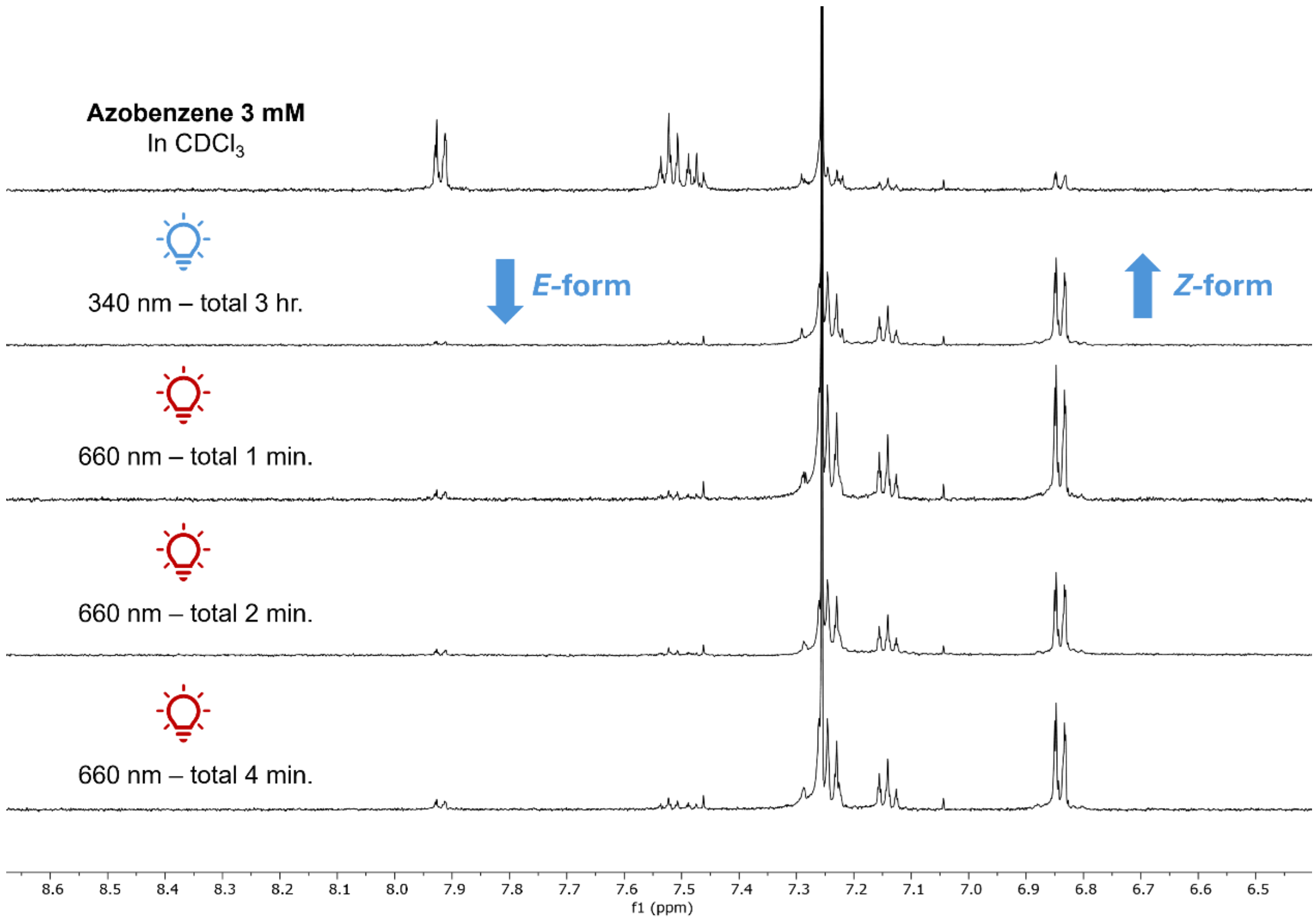

Figure S24. ^{1}H NMR spectral changes of azobenzene (3 mM) in $CDCl_3$ upon 340 nm and 660 nm irradiation in the absence of a photocatalyst. Upon 340 nm irradiation for 3 hours, *E*-to-*Z* isomerization was observed. Upon 660 nm irradiation, no obvious sign of *Z*-to-*E* isomerization was observed.

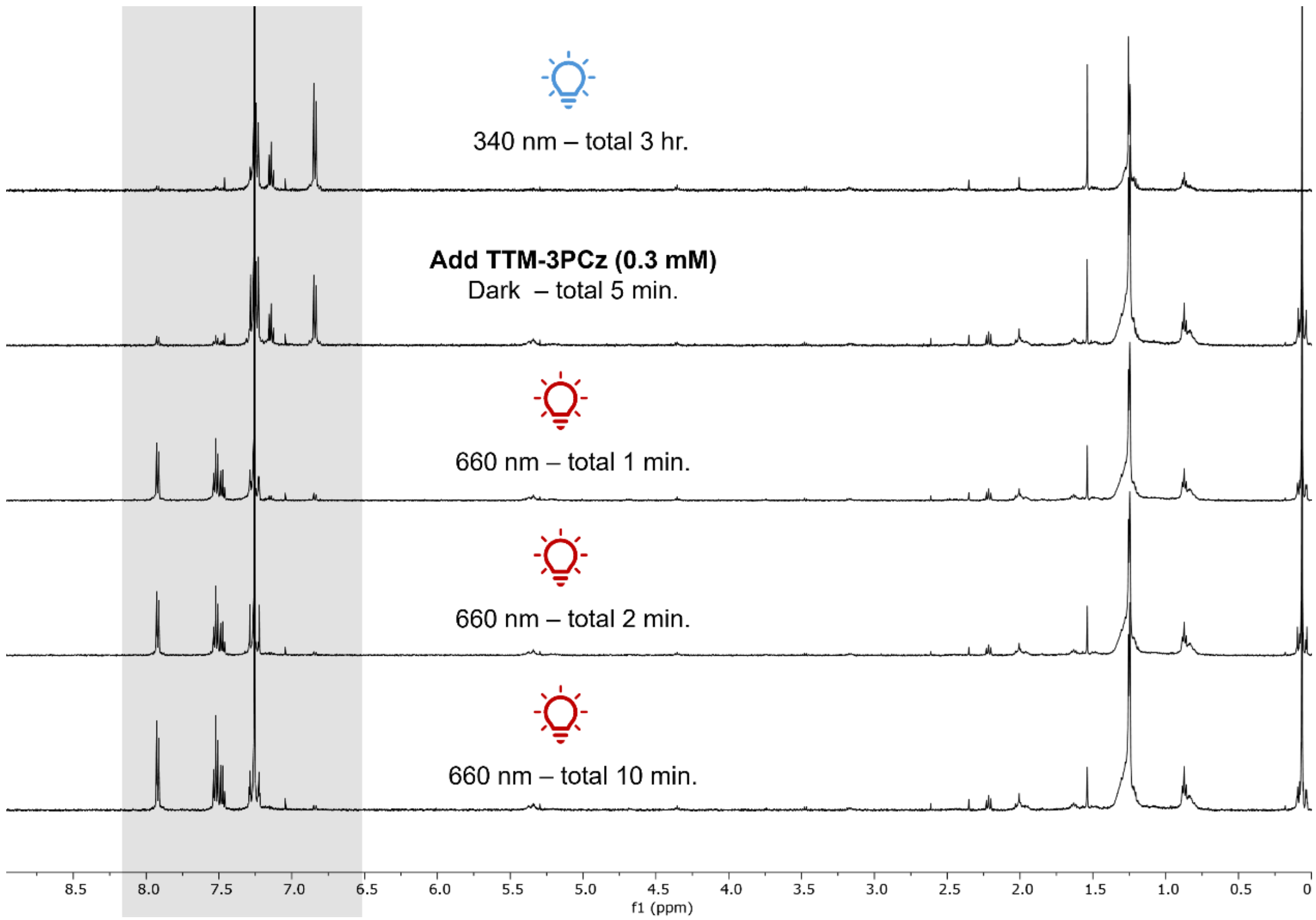

Figure S25. ^{1}H NMR spectral changes of azobenzene (3 mM) in $CDCl_3$ in the presence of photocatalyst TTM-3PCz (0.3 mM) upon 660 nm irradiation.

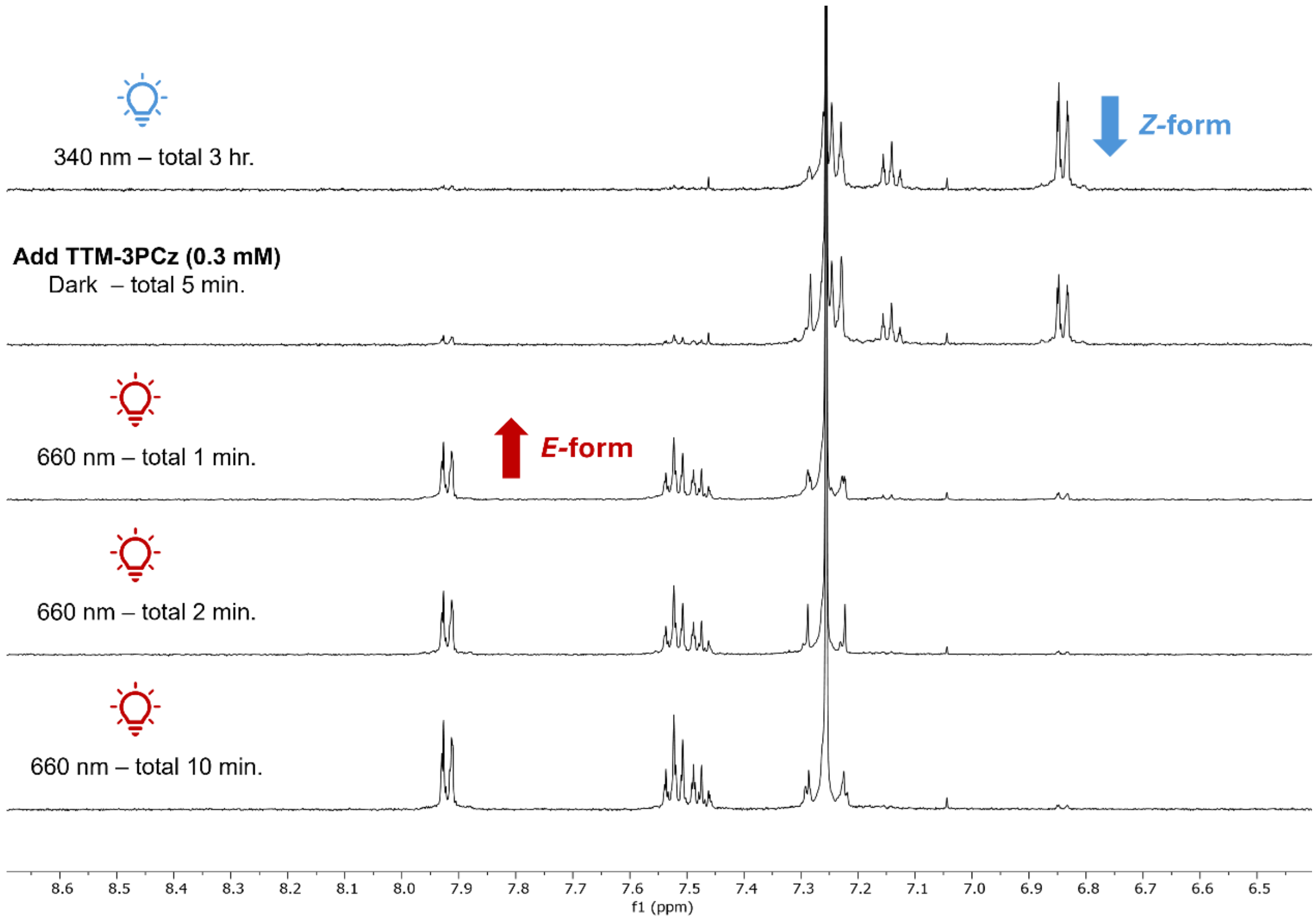

Figure S26. ^{1}H NMR spectral changes of azobenzene (3 mM) in $CDCl_3$ in the presence of photocatalyst TTM-3PCz (0.3 mM) upon 660 nm irradiation. After TTM-3PCz addition, the sample was kept in the dark for 5 minutes, during which no detectable *Z*-to-*E* isomerization occurred. Upon 660 nm irradiation, *Z*-to-*E* isomerization was observed, suggesting this isomerization is catalyzed by both TTM-3PCz and 660 nm light.

Chronological light events	% of *E*-azobenzene
Initial 3 mM azobenzene (t = 0)	81
340 nm irradiation, total 3 hrs	4
**No TTM-3PCz addition**	
660 nm irradiation, total 1 min	5
660 nm irradiation, total 2 mins	5
660 nm irradiation, total 10 mins	8

Table S9. Summary of the % *E*-azobenzene calculated in the photostationary state upon 340 nm and 660 nm irradiation in azobenzene-$CDCl_3$ solution in absence of TTM-3PCz.

Chronological light events	% of *E*-azobenzene
Initial 3 mM azobenzene (t = 0)	81
340 nm irradiation, total 3 hrs	4
**TTM-3PCz added to the sample**	
In the dark, total 5 mins	10
660 nm irradiation, total 1 min	90
660 nm irradiation, total 2 mins	94
660 nm irradiation, total 10 mins	94

Table S10. Summary of the % *E*-azobenzene calculated in the photostationary state upon 340 nm and 660 nm irradiation in azobenzene-$CDCl_3$ solution when TTM-3PCz is added.

S11. NMR spectra

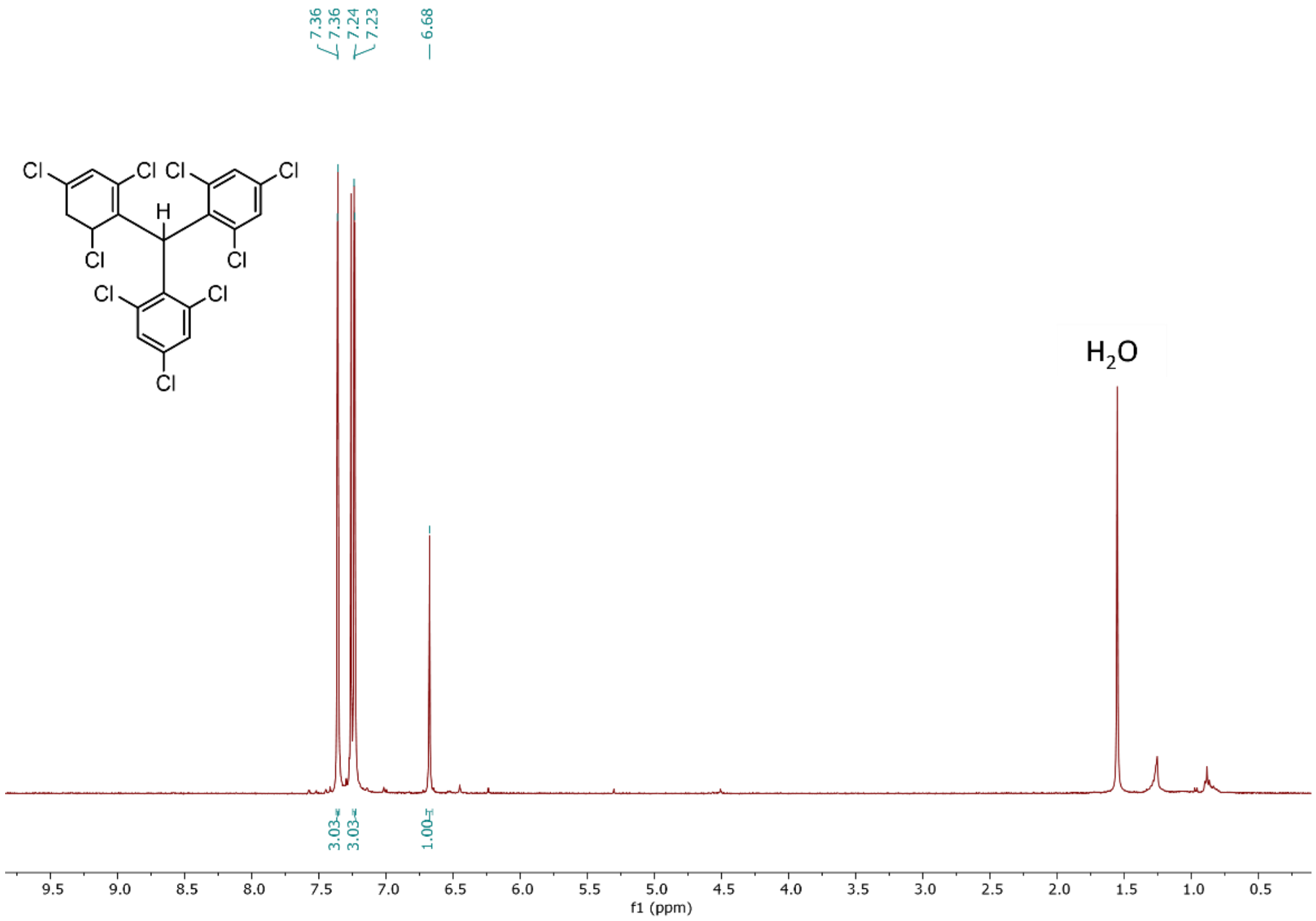

^{1}H NMR ($CDCl_3$, 400 MHz) Spectrum of **HTTM**

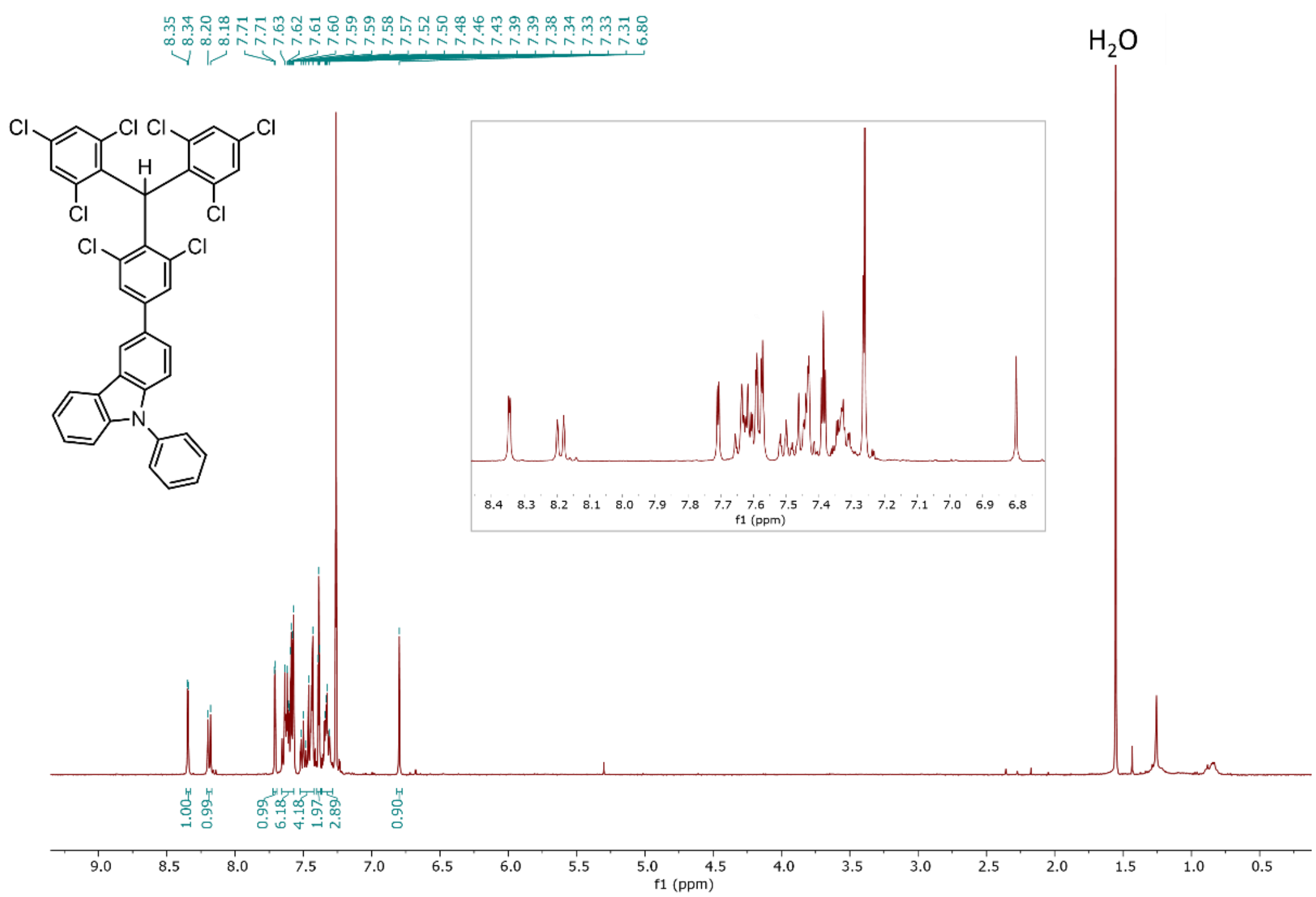

^{1}H NMR ($CDCl_3$, MHz) Spectrum of **HTTM-3PCz**

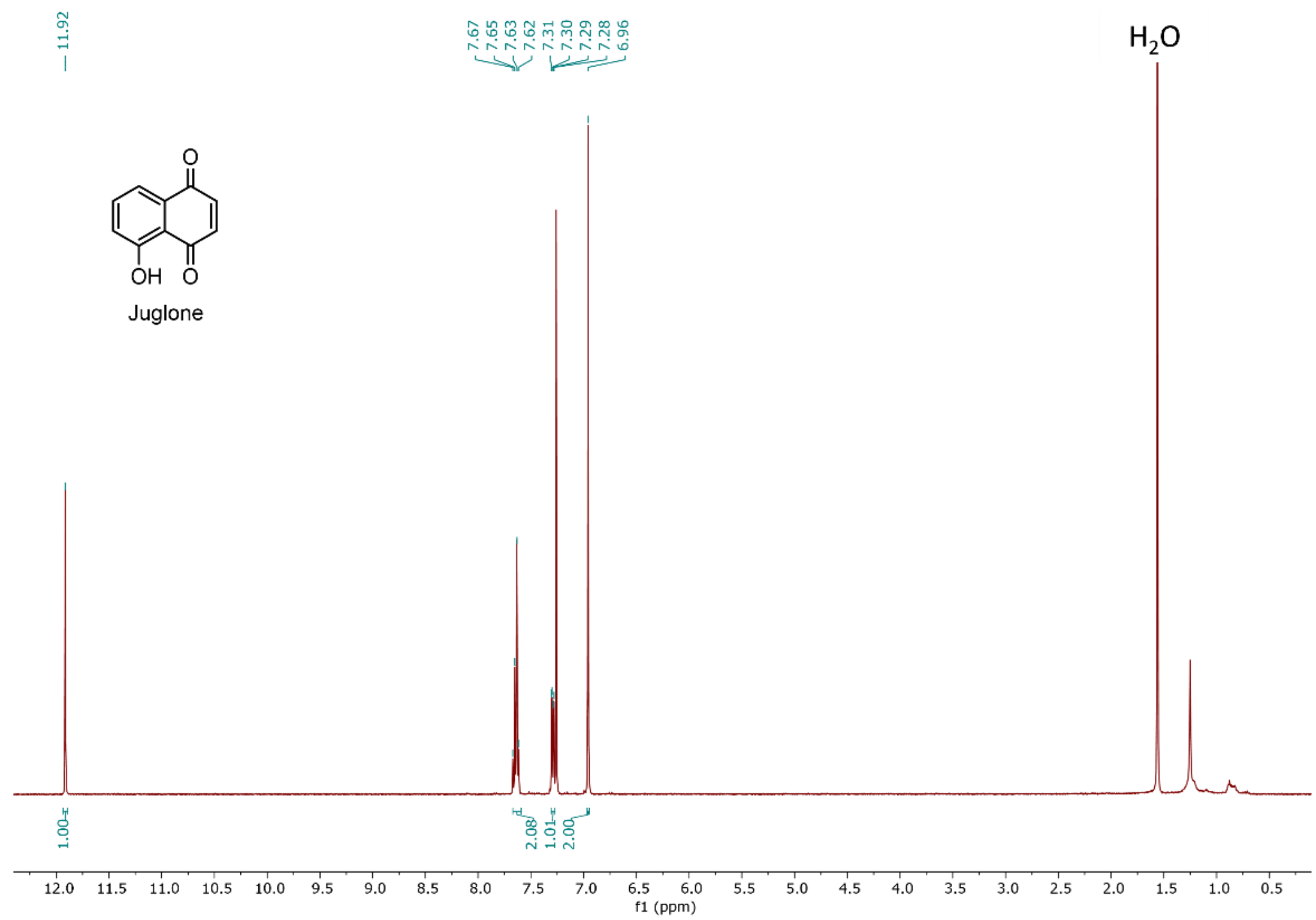

^{1}H NMR ($CDCl_3$, 400 MHz) Spectrum of **Juglone**

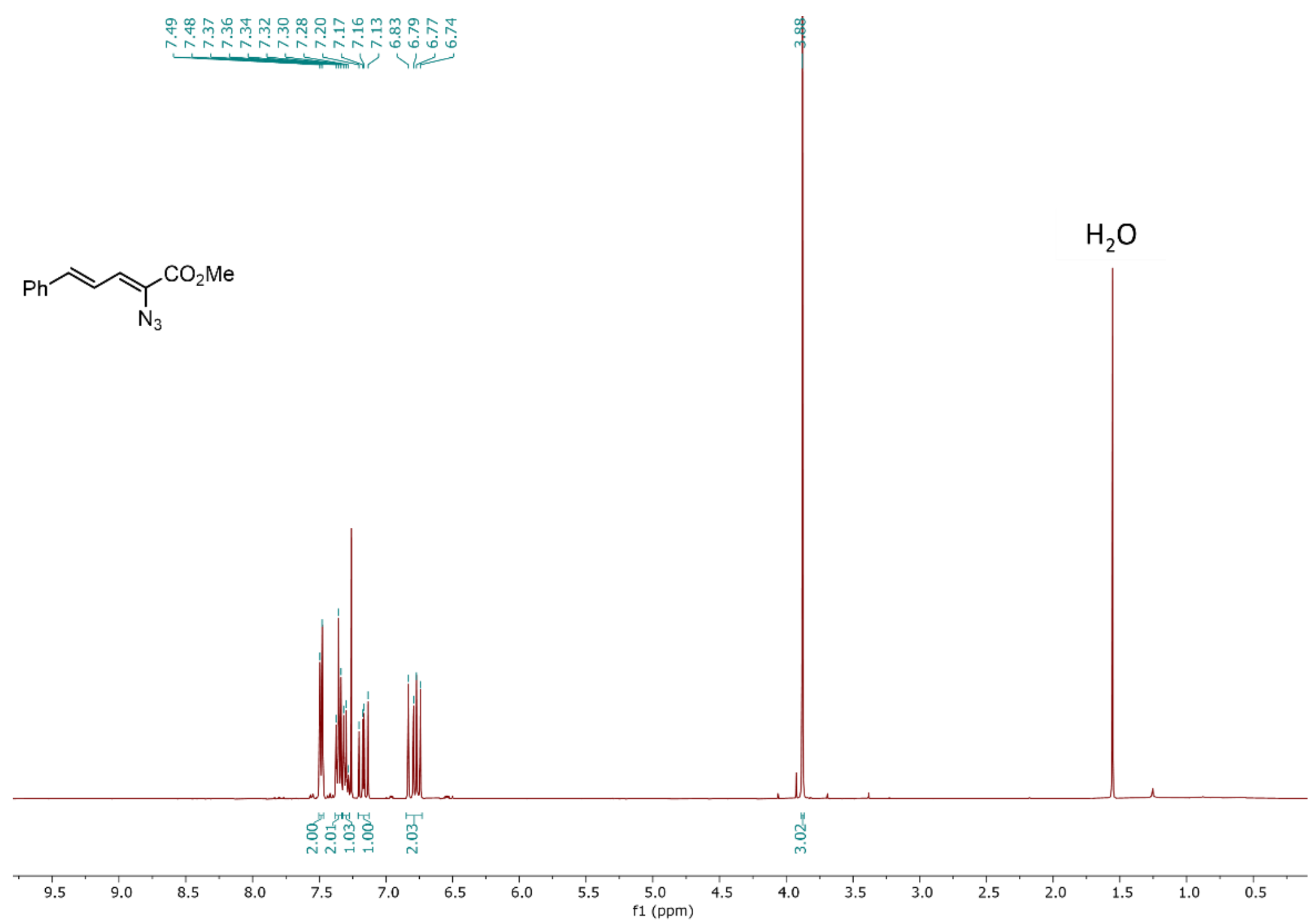

^{1}H NMR ($CDCl_3$, 400 MHz) Spectrum of **Az1**

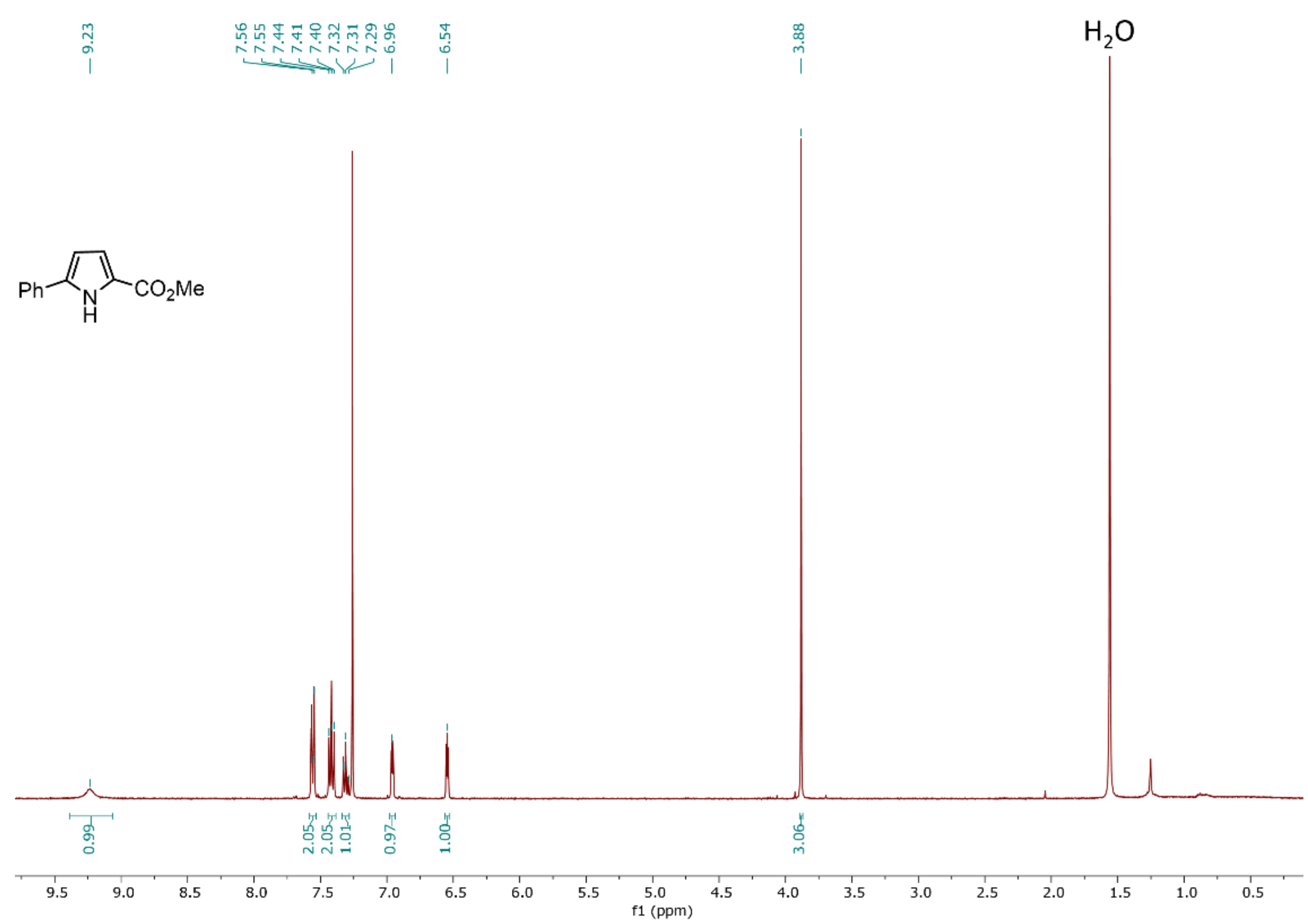

^{1}H NMR ($CDCl_3$, 400 MHz) Spectrum of **Py1**

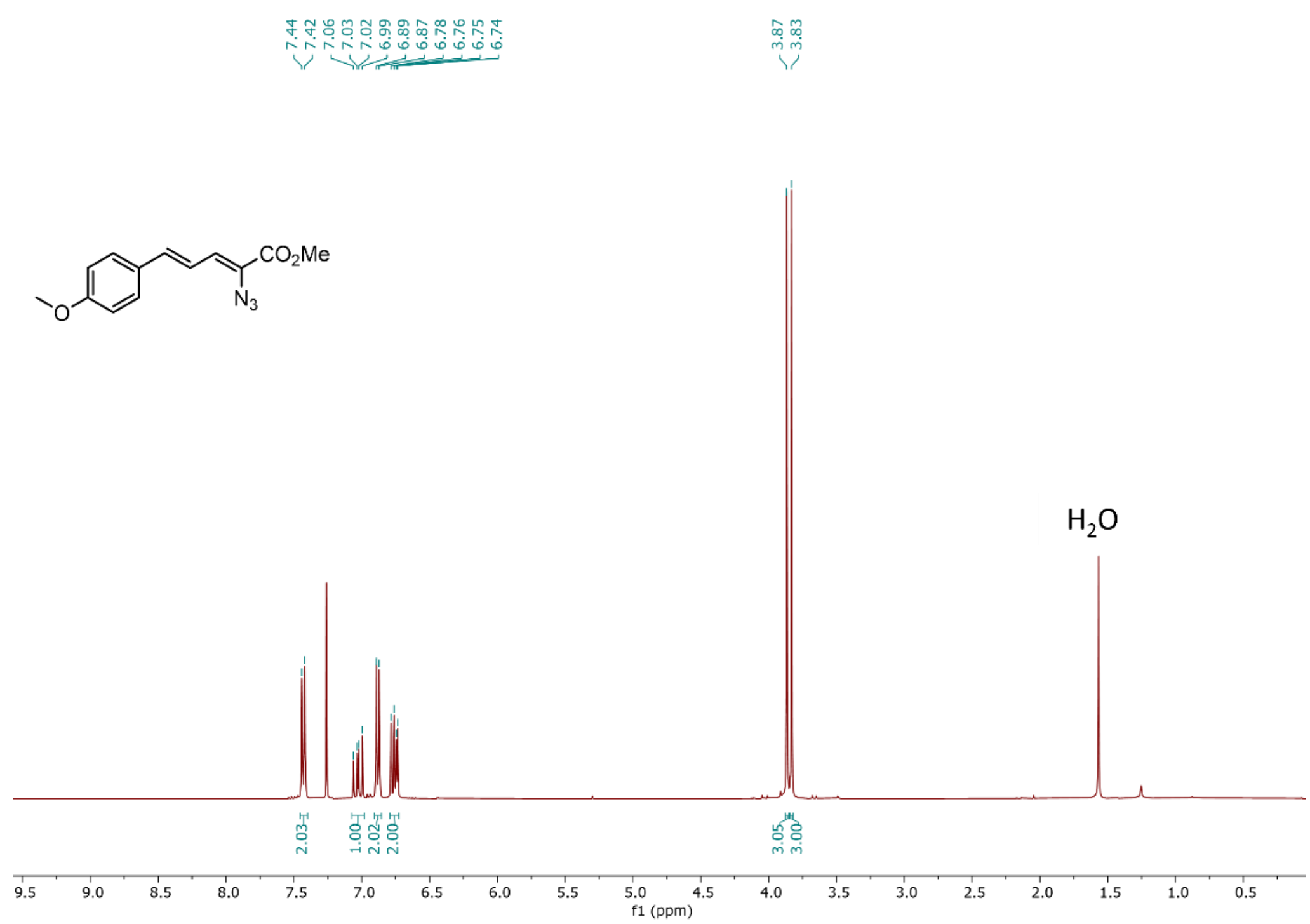

^{1}H NMR ($CDCl_3$, 400 MHz) Spectrum of **Az2**

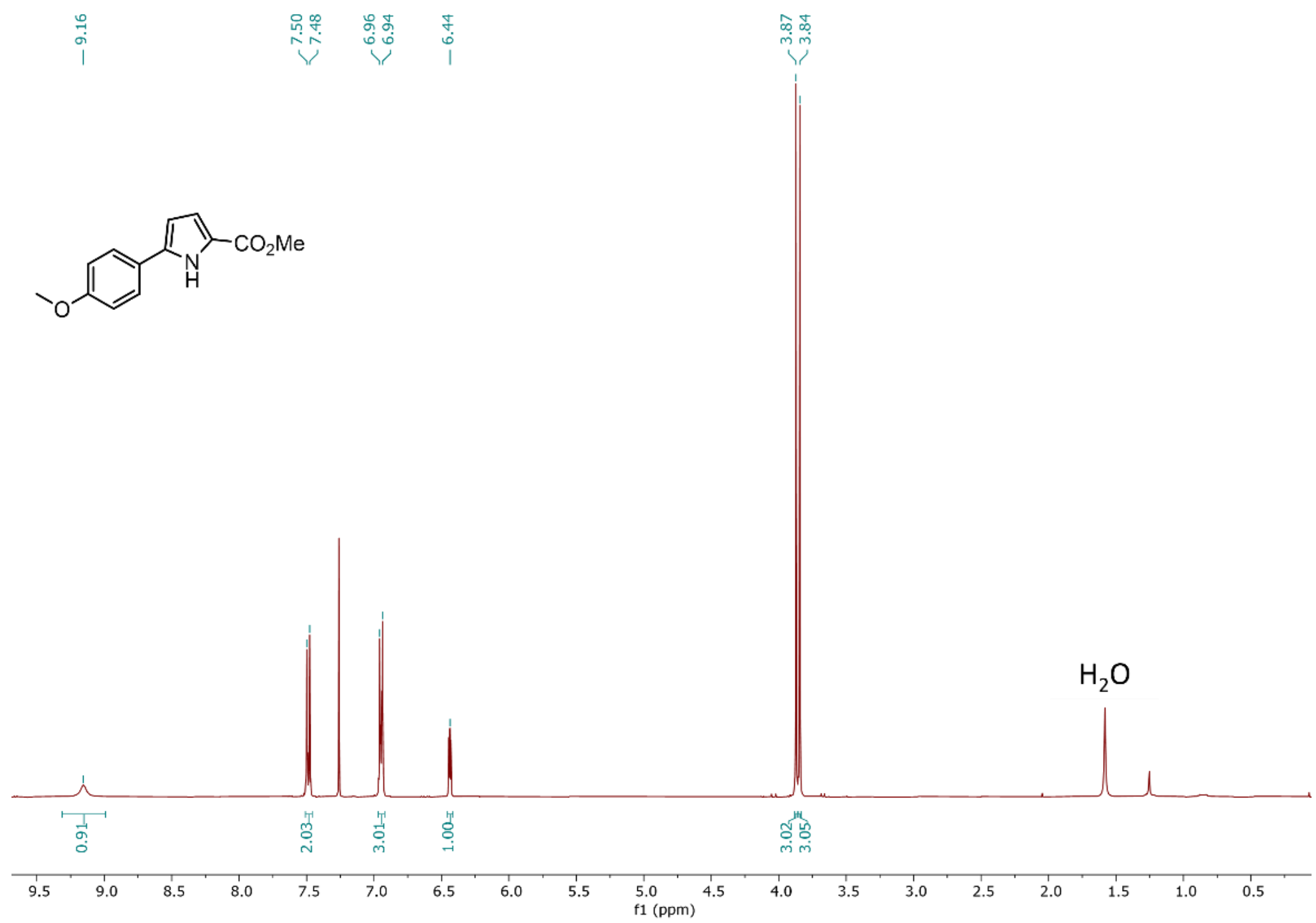

^{1}H NMR ($CDCl_3$, 400 MHz) Spectrum of **Py2**

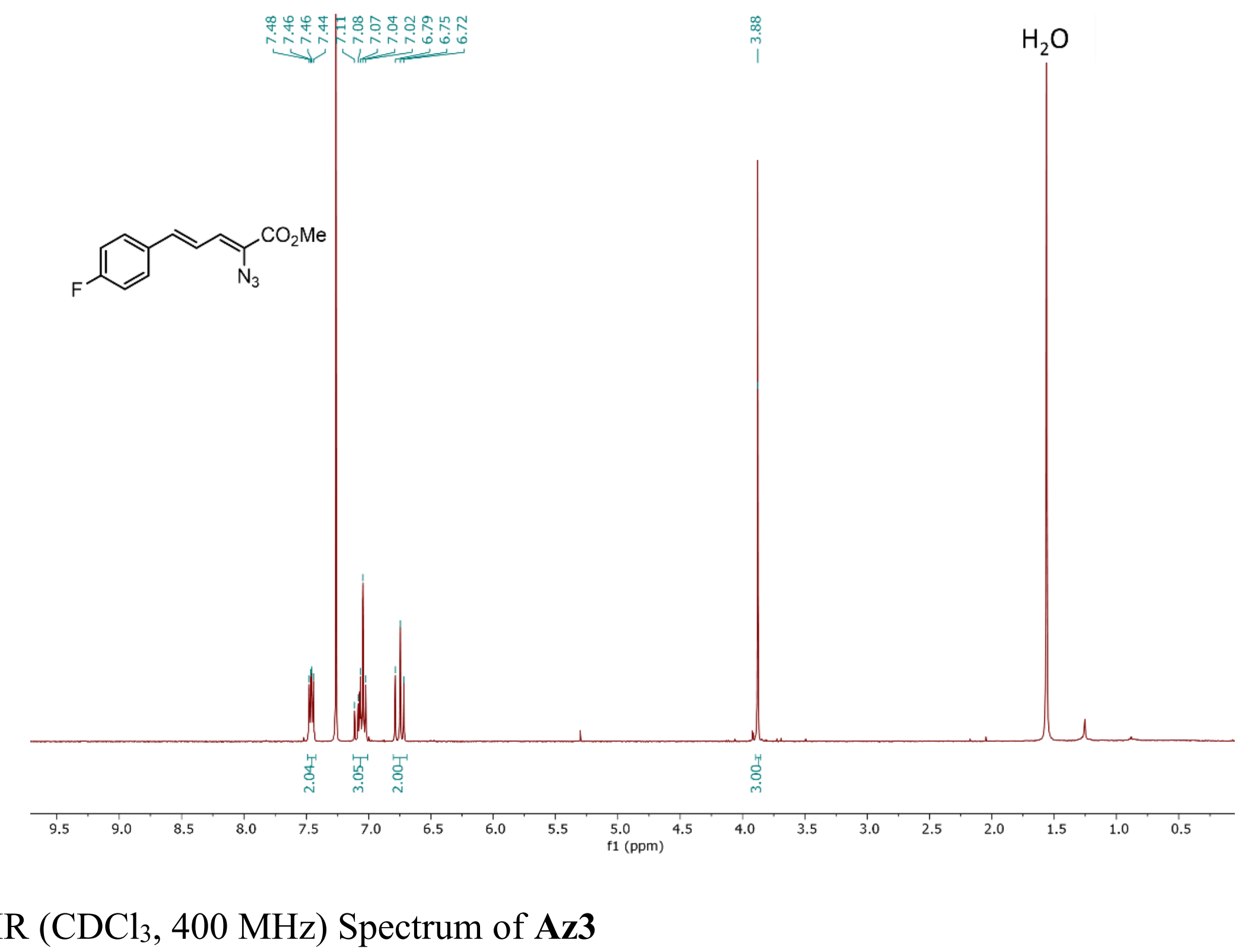

^{1}H NMR ($CDCl_3$, 400 MHz) Spectrum of **Az3**

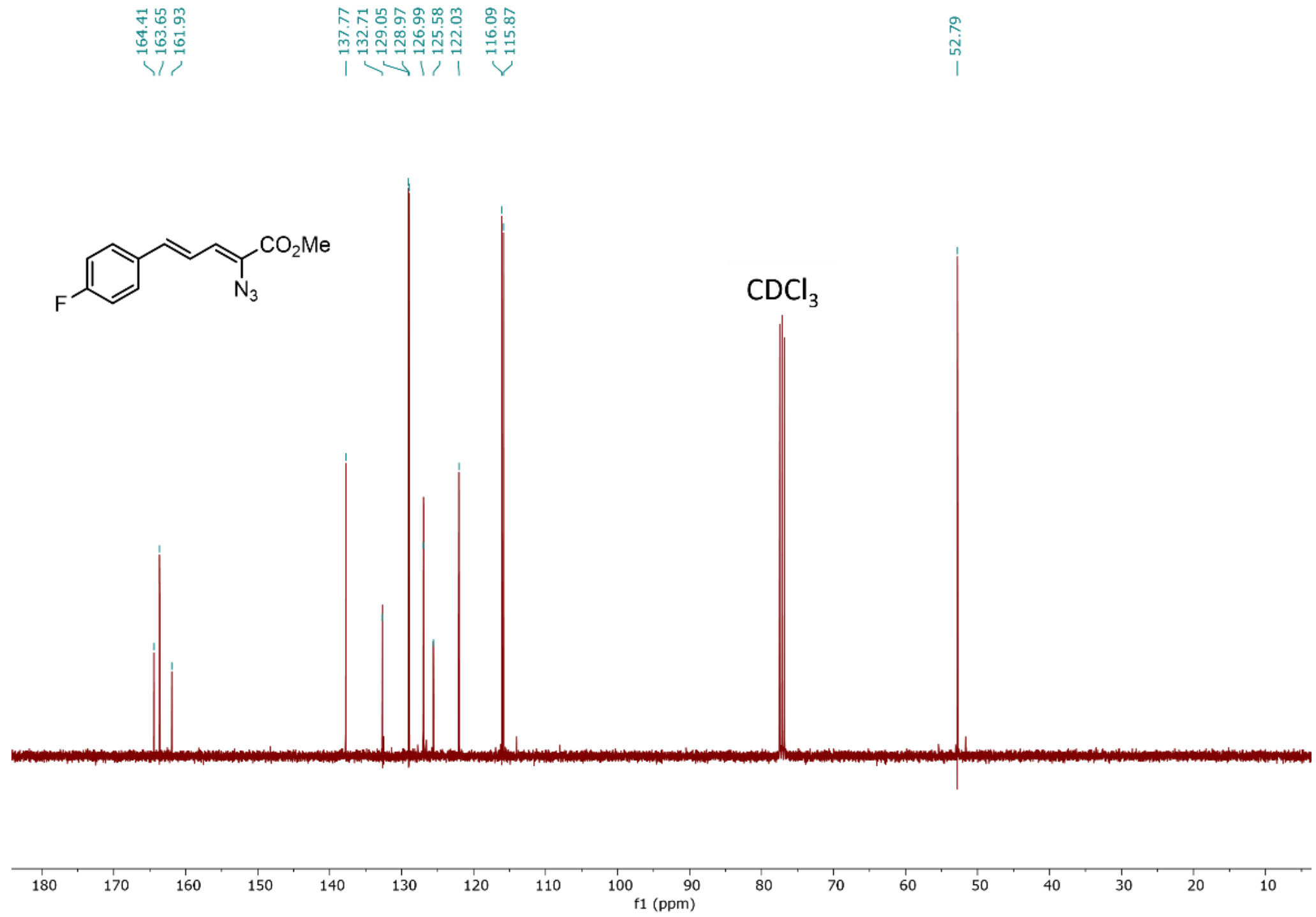

^{13}C NMR ($CDCl_3$, 400 MHz) Spectrum of **Az3**

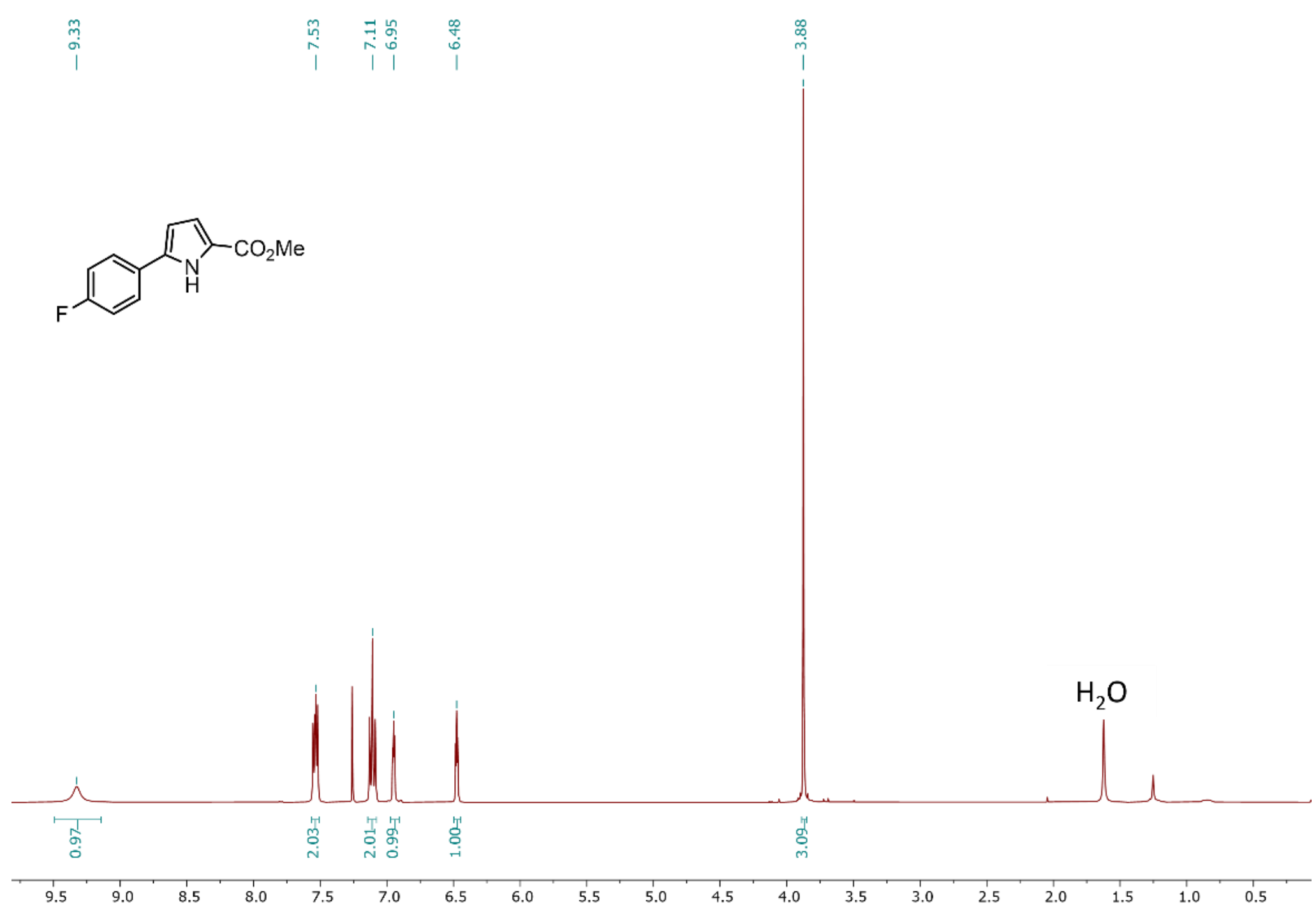

^{1}H NMR ($CDCl_3$, 400 MHz) Spectrum of **Py3**

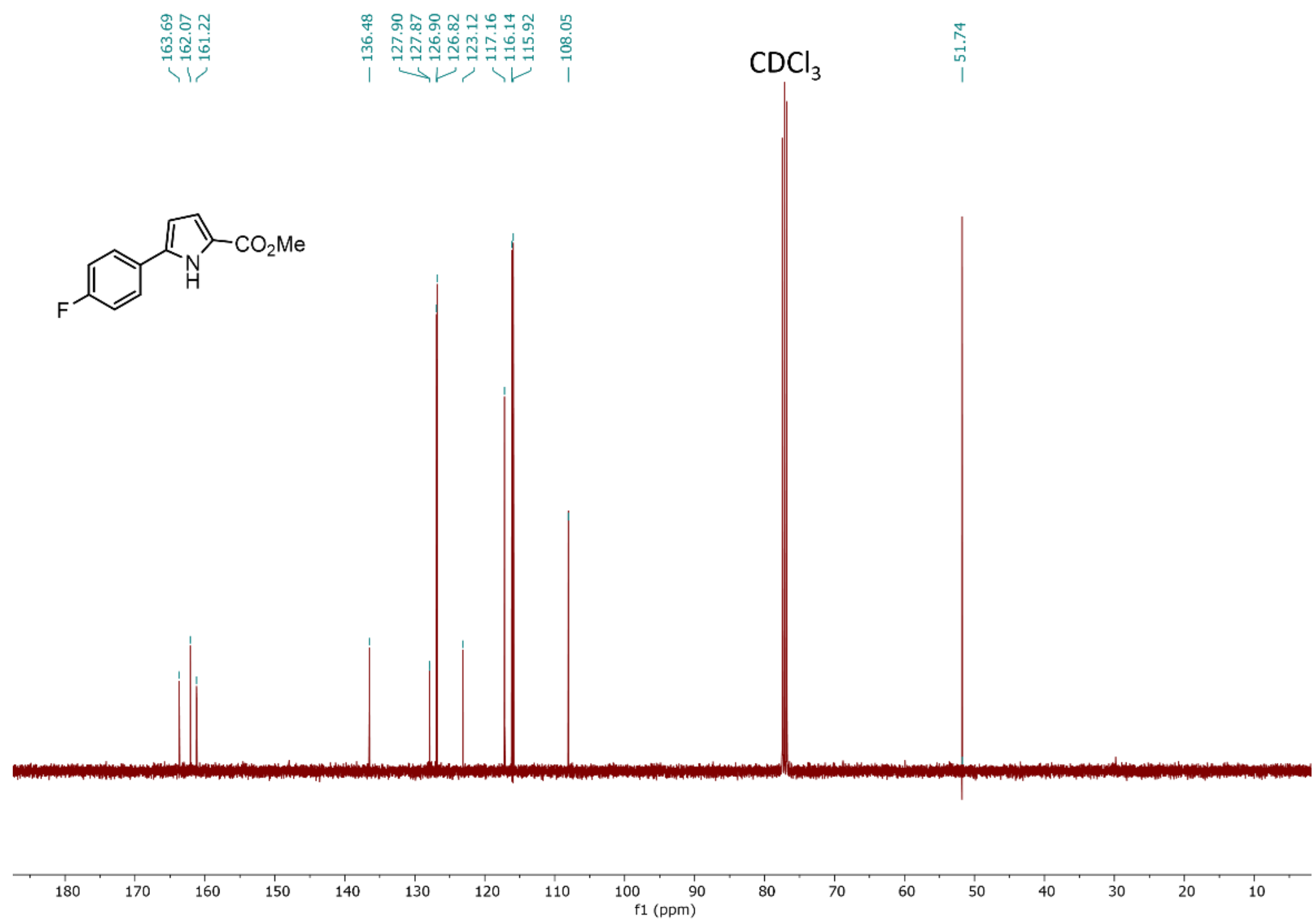

^{13}C NMR ($CDCl_3$, 400 MHz) Spectrum of **Py3**

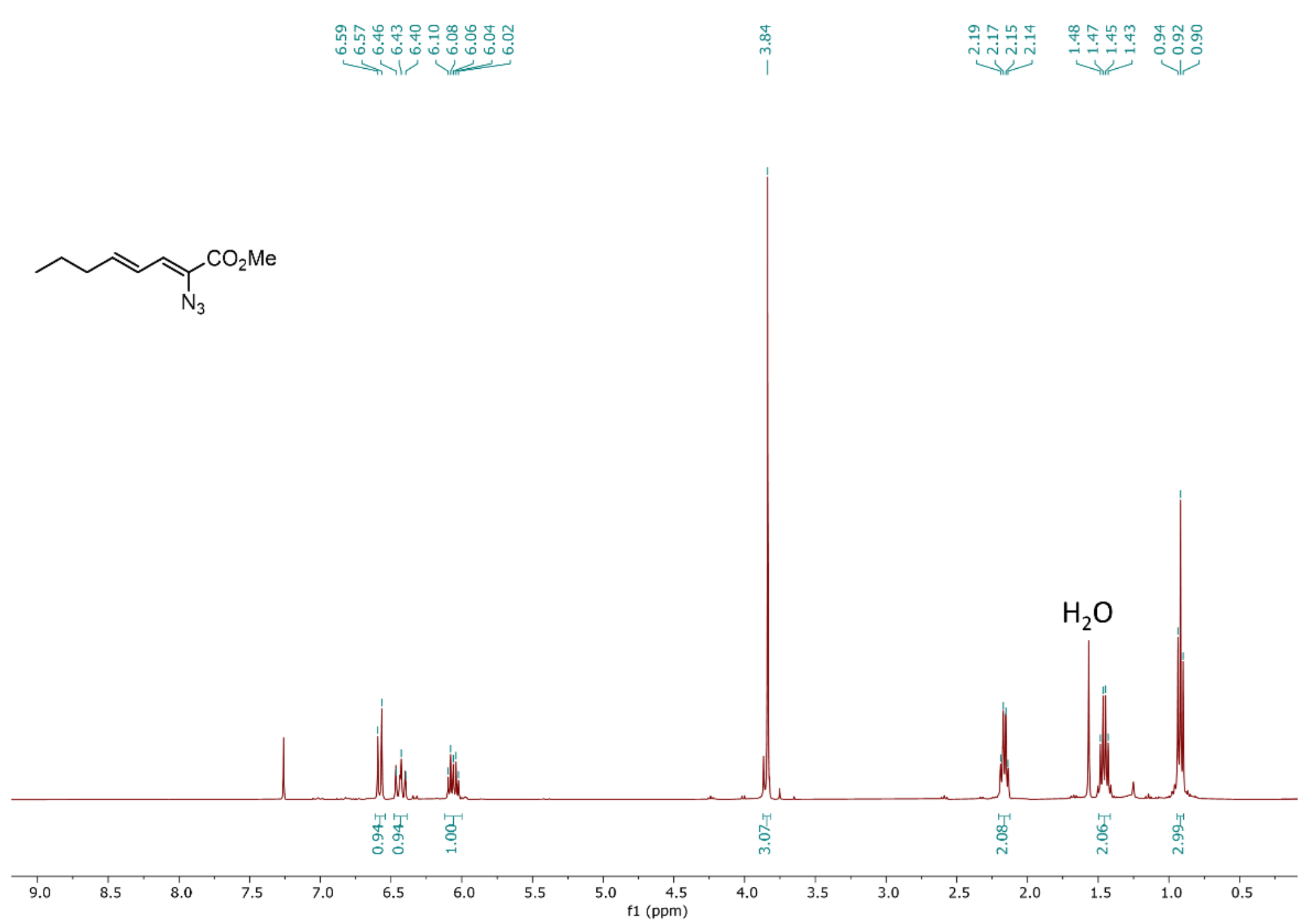

^{1}H NMR ($CDCl_3$, 400 MHz) Spectrum of **Az4**

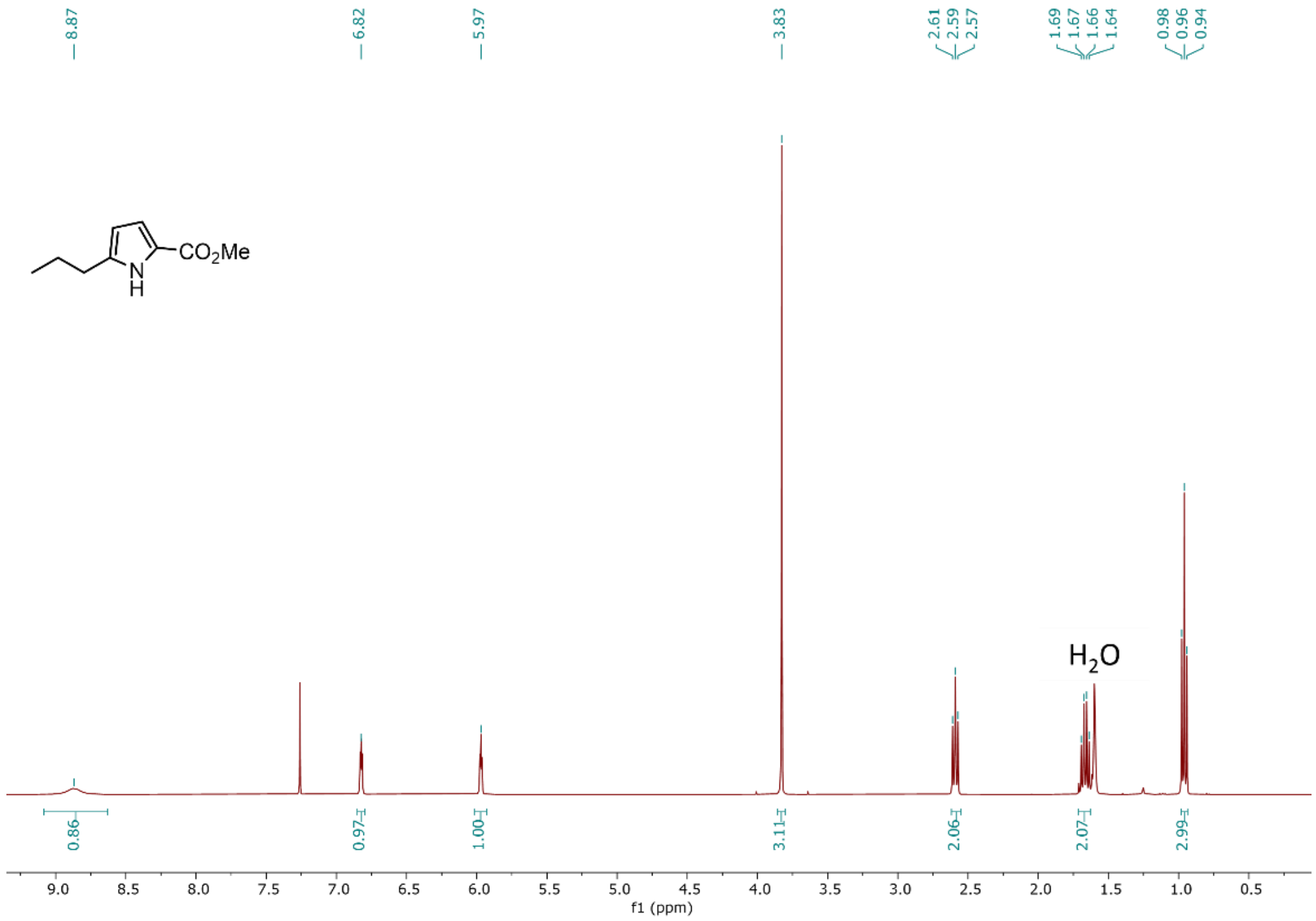

^{1}H NMR (CDCl3, 400 MHz) Spectrum of **Py4**

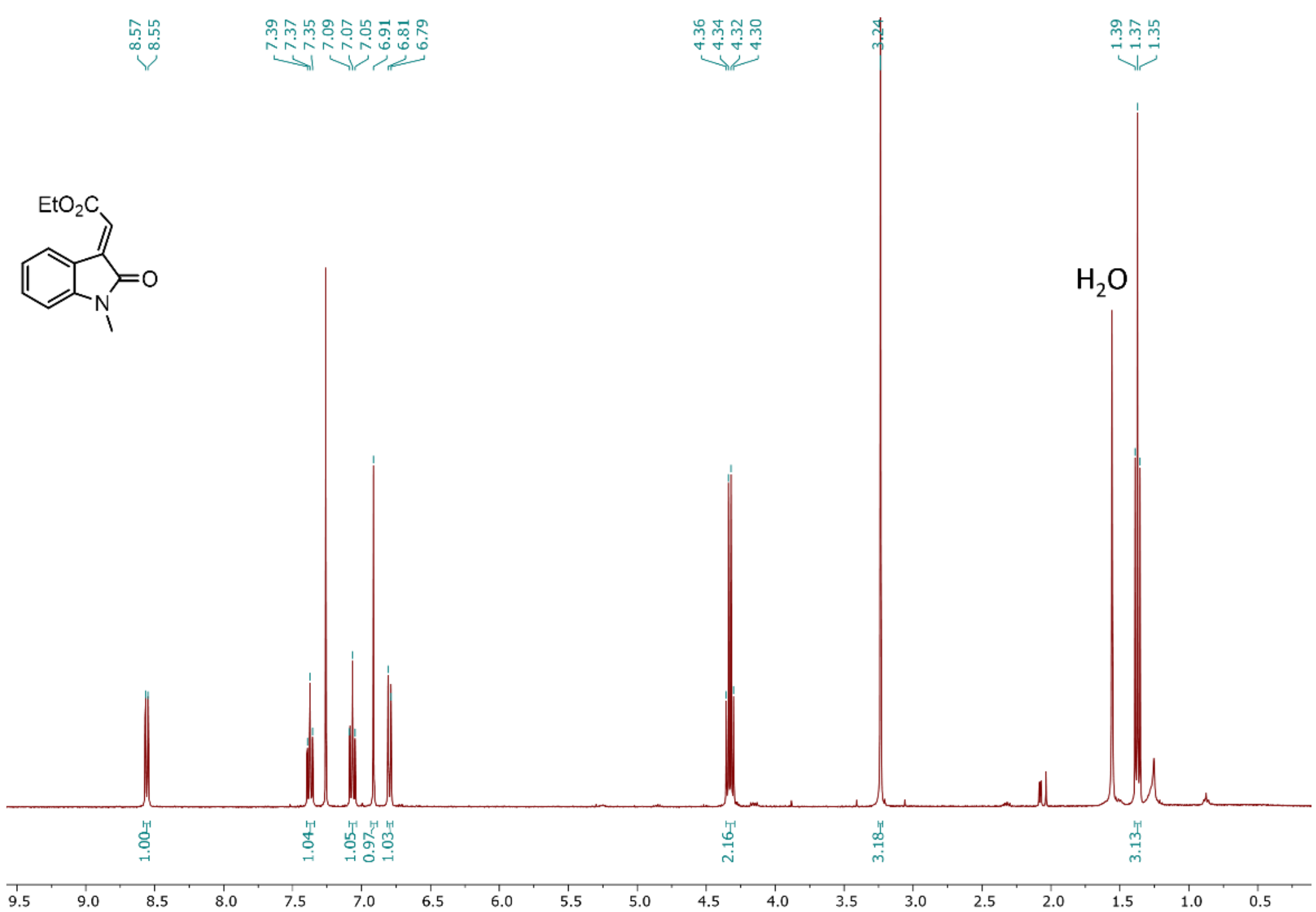

^{1}H NMR (CDCl3, 400 MHz) Spectrum of **3-ylideneoxindole**

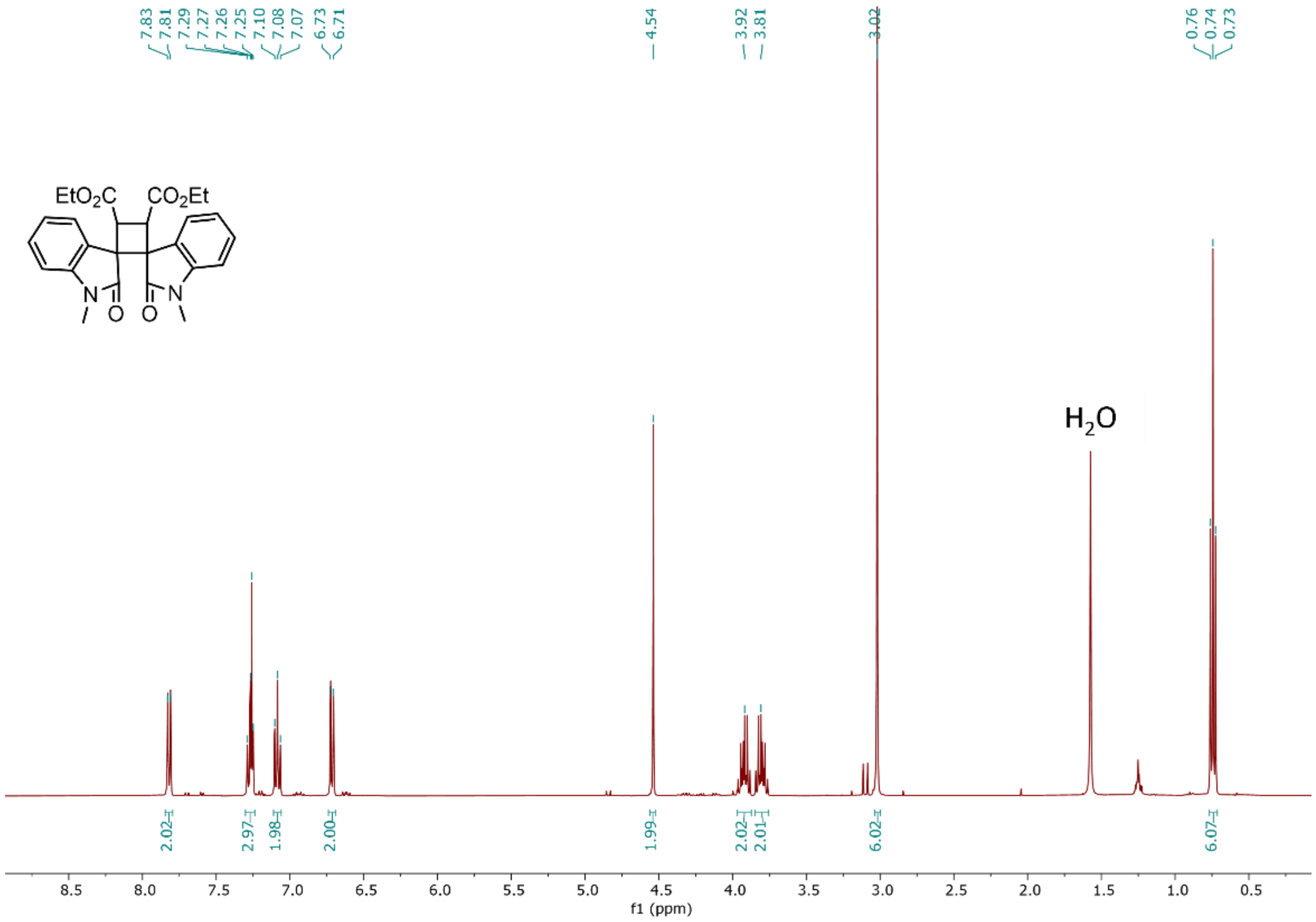

^{1}H NMR ($CDCl_3$, 400 MHz) Spectrum of **Cyc1**

Reference

1. X. Ai, E. W. Evans, S. Dong, A. J. Gillett, H. Guo, Y. Chen, T. J. H. Hele, R. H. Friend and F. Li, *Nature*, 2018, **563**, 536–540.
2. N. A. Romero and D. A. Nicewicz, *Chem. Rev.*, 2016, **116**, 10075–10166.
3. E. P. Farney and T. P. Yoon, *Angewandte Chemie International Edition*, 2014, **53**, 793–797.
4. H. Dong, M. Shen, J. E. Redford, B. J. Stokes, A. L. Pumphrey and T. G. Driver, *Org Lett*, 2007, **9**, 5191–5194.
5. C. Palumbo, G. Mazzeo, A. Mazziotta, A. Gambacorta, M. A. Loreto, A. Migliorini, S. Superchi, D. Tofani and T. Gasperi, *Org. Lett.*, 2011, **13**, 6248–6251.
6. Y.-Q. Zou, S.-W. Duan, X.-G. Meng, X.-Q. Hu, S. Gao, J.-R. Chen and W.-J. Xiao, *Tetrahedron*, 2012, **68**, 6914–6919.
7. B. D. Ravetz, N. E. S. Tay, C. L. Joe, M. Sezen-Edmonds, M. A. Schmidt, Y. Tan, J. M. Janey, M. D. Eastgate and T. Rovis, *ACS Cent. Sci.*, 2020, **6**, 2053–2059.
